# Amplifying Chinese physicians’ emphasis on patients’ psychological states beyond urologic diagnoses with ChatGPT – a multicenter cross-sectional study

**DOI:** 10.1097/JS9.0000000000001775

**Published:** 2024-07-02

**Authors:** Lei Peng, Rui Liang, Anguo Zhao, Ruonan Sun, Fulin Yi, Jianye Zhong, Rongkang Li, Shimao Zhu, Shaohua Zhang, Song Wu

**Affiliations:** a Department of Urology, Lanzhou University Second Hospital, Lanzhou, Gansu; b Department of Urology, South China Hospital, Shenzhen University, Shenzhen, Guangdong; c Department of Urology, The First Affiliated Hospital of Soochow University; d Department of Urology, Dushu Lake Hospital Affiliated to Soochow University, Medical Center of Soochow University, Suzhou Dushu Lake Hospital, Suzhou, Jiangsu; e West China School of Medicine, Sichuan University, Chengdu; f North Sichuan Medical College (University), Nanchong, Sichuan, People’s Republic of China

**Keywords:** artificial intelligence, chat generative pretrained transformer, large-scale language model, urology

## Abstract

**Background:**

Artificial intelligence (AI) technologies, particularly large language models (LLMs), have been widely employed by the medical community. In addressing the intricacies of urology, ChatGPT offers a novel possibility to aid in clinical decision-making. This study aimed to investigate the decision-making ability of LLMs in solving complex urology-related problems and assess their effectiveness in providing psychological support to patients with urological disorders.

**Materials and methods:**

This study evaluated the clinical and psychological support capabilities of ChatGPT 3.5 and 4.0 in the field of urology. A total of 69 clinical and 30 psychological questions were posed to the AI models, and both urologists and psychologists evaluated their response. As a control, clinicians from Chinese medical institutions responded to closed-book conditions. Statistical analyses were conducted separately for each subgroup.

**Results:**

In multiple-choice tests covering diverse urological topics, ChatGPT 4.0 was performed comparably to the physician group, with no significant overall score difference. Subgroup analyses revealed variable performance based on disease type and physician experience, with ChatGPT 4.0 generally outperforming ChatGPT 3.5 and exhibiting competitive results against physicians. When assessing the psychological support capabilities of AI, it is evident that ChatGPT 4.0 outperforms ChatGPT 3.5 across all urology-related psychological problems.

**Conclusions:**

The performance of LLMs in dealing with standardized clinical problems and providing psychological support has certain advantages over clinicians. AI stands out as a promising tool for potential clinical aid.

## Introduction

Highlights

- This study aimed to assess the clinical and psychological support capabilities of OpenAI’s ChatGPT versions 3.5 and 4.0 against a control group of healthcare professionals, particularly in the field of urology.
- The focus of the study was to assess their proficiency in diagnosing urological conditions and providing psychological guidance.
- The study reveals the potential of artificial intelligence (AI) in assisting clinical decision-making and psychological support while also highlighting that large language model (LLMs) should be used as aids rather than independent decision-makers in complex clinical scenarios.
- The findings highlight the importance of combining the capabilities of AI with the expertise, intuition, and experience of physicians.

In recent years, AI, particularly LLMs such as ChatGPT, has demonstrated formidable capabilities across diverse domains, garnering significant attention within the medical community^1–5^. Despite the impressive performance of LLMs in various fields, their application in medicine, particularly in urology, remains controversial^6–12^. Some scholars have delved into the potential of LLMs in urology. In a prior study, Kung *et al*.^13^ demonstrated that ChatGPT could successfully pass the United States Medical Licensing Examination (USMLE) without specialized training or user reinforcement. Davis *et al*.’s^14^ study demonstrates that ChatGPT can output more consistently and clearly readable answers to urology-related questions; however, there remain certain knowledge blind spots to be improved. In contrast, Huynh *et al*.^15^ reported that ChatGPT showed less than 30% accuracy on the Urologic Self-Assessment Research Program (USARP) and noted that responses to open-ended questions appeared ambiguous. Cocci and colleagues used real clinical reasoning for data entry into ChatGPT and then counseled on diagnostic and therapeutic measures, which showed that ChatGPT is indeed an interactive tool for providing medical information online, offering the possibility of improving healthcare outcomes and patient satisfaction. However, the appropriateness of urology case responses was inadequate and of low quality. This emphasizes the importance of comprehensively evaluating and using outputs generated by natural language processing when addressing health-related issues^16^. Cil and Dogan evaluated the performance of ChatGPT in the diagnosis and treatment of urological disorders, especially kidney stones, as well as the learning and adaptive capacity of ChatGPT through a number of structured questions. It is easy to see that although the authors of this study are in favor of the inclusion of AI in healthcare, they also express the concern that the completeness of LLMs’ answers to complex questions is still lacking and that the current application of LLMs is not yet subject to effective professional supervision^17^. While numerous studies have explored the potential of LLMs in clinical diagnosis and treatment, there is a dearth of exploration around clinical psychological assistance^18^.

In our observations, practitioners tend to prioritize treating the disease over offering adequate psychological support to patients despite the longstanding advocacy for fostering a compassionate patient–physician relationship. While the concept of ‘comforting the patient’ has been emphasized, psychological factors remain an under-addressed aspect of various medical treatments. ChatGPT, initially conceptualized as a ‘chatbot,’ prompted us to explore the potential of LLMs as a complementary tool in addressing psychosocial scenarios, particularly within urological contexts (e.g. anxiety related to urinary incontinence, erectile dysfunction, and presurgical patients)^1,19,20^. While previous studies have focused on evaluating the rationality and feasibility of LLMs for decision-making in medicine, this marks the first instance, to our knowledge, where an AI has been appraised for its capacity to provide emotionally supportive responses to medical situations.

## Materials and methods

### Protocol

This study was written and reported according to the STROCSS 2019 Guideline (Supplemental Digital Content 1, http://links.lww.com/JS9/C993: Strengthening the reporting of cohort studies in surgery)^21^.

### Study design

**Figure 1:**
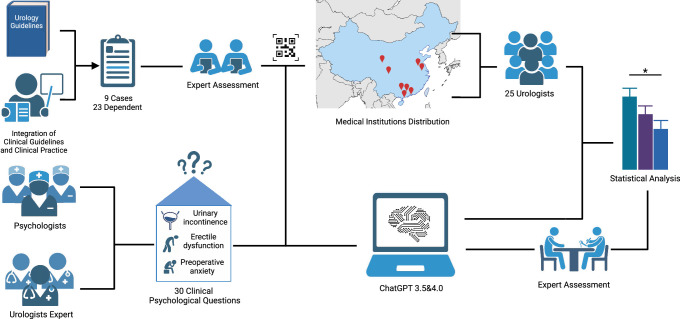
Research overview.

This study aimed to evaluate the clinical expertise and psychological support capabilities of OpenAI’s ChatGPT, versions 3.5 and 4.0 (August 2023 version). The research design involved a comparative analysis of these generative LLMs against a control group comprising medical professionals. To maintain objectivity and minimize bias, no custom commands were utilized to enhance the medical knowledge of the models. Each question was independently presented utilizing the ‘New Chat’ feature and repeated thrice to ensure consistency in the results. The overall study design and workflow are illustrated in Figure 1. The construction and description of ‘selection of LLMs’ and ‘population’ is presented in Supplementary File 1, Supplemental Digital Content 2, http://links.lww.com/JS9/C994.

### Clinical issues

To evaluate the proficiency of LLMs in diagnosing and treating urological diseases, we formulated 69 complex structured clinical issues. These issues, derived from the ‘European Association Urology Guidelines’^22^ and informed by expert clinical experience, encompassed multiple types of diseases affecting the urinary system. Each issue underwent review by a senior urologist to ensure clinical relevance and a sufficient level of difficulty. Supplementary File 1, Supplemental Digital Content 2, http://links.lww.com/JS9/C994 provides specific details on the development of clinical issues, and Supplementary File 2, Supplemental Digital Content 3, http://links.lww.com/JS9/C995 provides detailed information about the issue papers.

### Psychological issues

The study also assessed the LLMs’ ability to provide clinical psychological guidance and support. We formulated 30 psychological support issues, specifically tailored to urological scenarios like urinary incontinence and sexual dysfunction in collaboration with urologists and clinical psychologists. These issues were submitted to distinct versions of ChatGPT for Q&A (Supplementary File 3, Supplemental Digital Content 4, http://links.lww.com/JS9/C996). Two senior clinical psychologists independently devised three scales based on the Quick Inventory of Depressive Symptomatology Depression Rapid Scale^23^ and the Hospital Anxiety and Depression Scale^24^, aligning them with the practical clinical experience of urologists (Supplementary Table 1, Supplemental Digital Content 5, http://links.lww.com/JS9/C997). Subsequently, two additional psychologists utilized these scales as a reference to blindly assess the plausibility and feasibility of the ChatGPT responses. The total score across all three scales was capped at 100, and responses rated at 80 or higher were considered valid suggestions.

### Outcomes

The outcome indicators consisted of two categories. Primary outcomes were obtained through comparison of differences between ChatGPT and urologists’ overall performance in clinical issues and a comparison of differences in psychologists’ ratings of the two AI models according to specified scales. Secondary outcomes were obtained through a comparison of differences in subgroup classification according to disease type or topic area. Percentage correct, percentage scored, and specific responses of the two AI models to psychological issues.

### Statistical analysis

In this study, Prism GraphPad 9.0 software was used for statistical analysis and data visualization. The performance of AI LLMs (ChatGPT versions 3.5 and 4.0) was compared with that of clinicians in the Urology Clinical Knowledge Test using the Wilcoxon signed-rank test, catering to potentially nonnormal data distribution. The test’s effect size was based on the groups’ mean scores, and group rate comparisons were made using the *χ* ^2^ test at a significance level of *P*=0.05. To further explore differences between LLM versions and physician ranks (resident vs. attending), subgroup analyses were conducted with the Kruskal–Wallis test. Scores are reported as mean (range). Thorough data preparation and coding were undertaken before analysis to ensure accuracy and reliability.

## Results

### Overview of study implementation and completion

A total set of 69 multiple-select questions focused on clinical expertise in urology was developed for this study. It comprised nine case study issues (with a total of 46 subissues) and 23 stand-alone issues, encompassing the major areas of urology. To thoroughly assess LLMs’ performance, we conducted a meticulous testing and scoring record of these issues utilizing ChatGPT versions 3.5 and 4.0 (https://chat.openai.com/).

From August to September 2023, this study recruited 25 urology specialists, comprising 11 residents and 14 attending physicians, representing eight prominent medical institutions across China. All participants underwent the test in closed-book conditions utilizing a specially designed ‘QR code’ mobile application. The total score for the entire paper was 325.

### Primary outcomes in clinical issues

#### Overall assessment

**Figure 2:**
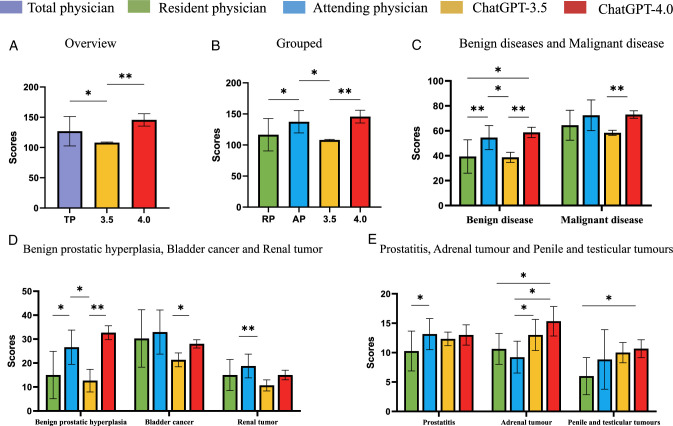
Assessment of artificial intelligence model performance versus clinician comparison group (A. Overall, B. Grouped, C. Benign and Malignant diseases, D. Benign prostatic hyperplasia, bladder cancer, and renal tumor, E. Prostatitis, adrenal tumor, and penile, and testicular tumors).

Based on the assessment results, the ChatGPT 4.0 subgroup outperformed the ChatGPT 3.5 subgroup, and this difference was statistically significant (*P*=0.0031). However, no statistically significant difference was observed when comparing the ChatGPT 4.0 subgroup to the physician group (Fig. 2A).

Moreover, the AI group scored [126.80 (107–153)] points. The ChatGPT 4.0 subgroup averaged 145.67 points, and the ChatGPT 3.5 subgroup averaged 108 points. When comparing AI and physicians, there was no significant difference in scores (Supplementary Fig. 1F, Supplemental Digital Content 6, http://links.lww.com/JS9/C998).

### Secondary outcomes in clinical issues

#### Grouping based on model version and physician title

With the resident group achieving a score of [116.50 (74–161)] points and the attending group averaging [137.40 (112–179)] points, statistical analysis showed that the scores and performance of the attending physician group surpassed those of the resident group, and the difference was statistically significant (*P*=0.0321). The attending group outperformed ChatGPT 3.5, and the difference between the two groups was statistically significant (*P*=0.0168) (Fig. 2B). The groups’ scores on each specific topic are shown in Supplementary Fig 1A–E, Supplemental Digital Content 6, http://links.lww.com/JS9/C998.

#### Grouping based on the urological knowledge section

The clinical questions were categorized according to the benign and malignant nature of the disease, and the results of the scores and statistical figures for each group are shown in Figure 2C (Supplementary File 3, Supplemental Digital Content 4, http://links.lww.com/JS9/C996 and Supplementary Fig 2, Supplemental Digital Content 7, http://links.lww.com/JS9/C999).

Subsequently, we categorized the clinical issues into 12 groups according to urological sections, including benign prostatic hyperplasia, prostatitis, bladder cancer (BC), renal tumor, adrenal tumor, penile and testicular tumors, urinary system tuberculosis, hydronephrosis, anatomy of the urinary system, prostate cancer, kidney transplant, and tumor general. In benign prostatic hyperplasia-related issues, statistical analysis indicated that ChatGPT 4.0 scored significantly higher compared with ChatGPT 3.5 (*P*=0.0033). The attending group achieved higher scores than the resident group (*P*=0.0031) and ChatGPT 3.5 (*P*=0.0068), with these differences being statistically significant (Fig. 2D). Among all the BC-related issues, statistical analysis exhibited that ChatGPT 4.0 obtained higher and statistically different scores compared with ChatGPT 3.5 (*P*=0.0265), whereas no statistical differences were observed based on the comparisons between the other groups. Furthermore, there was a tendency for the AI group to exhibit lower scores on BC-related issues than the physician groups (Fig. 2D). In the renal-tumor-related issues, statistical analysis showed that the attending group scored higher than ChatGPT 3.5 and the difference was statistically significant (*P*=0.0172). However, the differences between the other groups were not statistically significant (Fig. 2D). Statistical analyses exhibited that the attending group scored significantly higher on prostatitis-related issues than the resident group, and this difference was statistically significant (*P*=0.0285). While LLMs in the AI group scored closer to each other and outperformed the attending group, there was still a trend toward lower scores (Fig. 2E). Statistical analysis showed that ChatGPT 4.0 attained higher scores than the attending and resident groups in adrenal-tumor-related issues, and the differences between the groups were statistically significant (*P*=0.0032, 0.0169). Additionally, ChatGPT 3.5 attained higher scores than the attending group with statistically significant differences (*P*=0.0471) (Fig. 2E). Regarding the penile-tumor-related and testicular-tumor-related issues, statistical analysis revealed a statistically significant difference between ChatGPT 4.0 and the resident group (*P*=0.032) (Fig. 2E). Details of the comparisons between all groups regarding the urological knowledge section can be seen in Table 1.

**Table 1:** Scores of the groups in the urological knowledge section.

Outcomes	Resident physician	Attending physician	ChatGPT 3.5	ChatGPT 4.0
BPH	15.00 (2–34)	26.62 (11–41)^a^	12.67 (9–18)^b^	32.67 (31–36)^c^
Prostatitis	30.27 (4–16)	32.92 (9–18)^a^	21.33 (11–13)	28 (11–14)
Bladder cancer	30.27 (13–52)	32.92 (19–49)	21.33 (18–23)^b^	28 (26–29)^c^
Renal tumor	15.00 (6–27)	18.77 (6–24)	10.67 (8–12)^b^	15.00 (13–17)
Adrenal tumor	10.64 (6–15)	9.231 (4–13)	13.00 (10–15)^b^	15.33 (13–18)^a^ ^c^
Penile and testicular tumors	6.00 (2–12)	8.84 (2–17)	10.00 (9–12)	10.67 (9–12)^a^
Urinary system tuberculosis	10.55 (5–17)	11.31 (5–20)	11.67 (5–15)	11.67 (10–15)
Hydronephrosis	2.909 (0–5)	2.308 (0–5)	0.667 (0–2)	0
Anatomy of the urinary system	2.09 (0–2)	2.30 (0–2)	2.00 (2–2)	2.00 (2–2)
Prostate cancer	1.455 (0–2)	1.615 (0–5)	1.333 (0–2)	2.00 (2–2)
Kidney transplant	9.455 (5–15)	9.154 (2–15)	10.33 (7–12)	13.33 (10–15)
Tumor general	1.09 (0–2)	1.08 (0–5)	2.00 (2–2)	2.00 (2–2)

BPH, benign prostatic hyperplasia.

a Attending physician, ChatGPT 3.5, ChatGPT 4.0 versus resident physician, *P*<0.05.

b ChatGPT-3.5, ChatGPT 4.0 versus attending physician, *P*<0.05.

c ChatGPT-3.5 versus ChatGPT 4.0, *P*<0.05.

**Figure 3:**
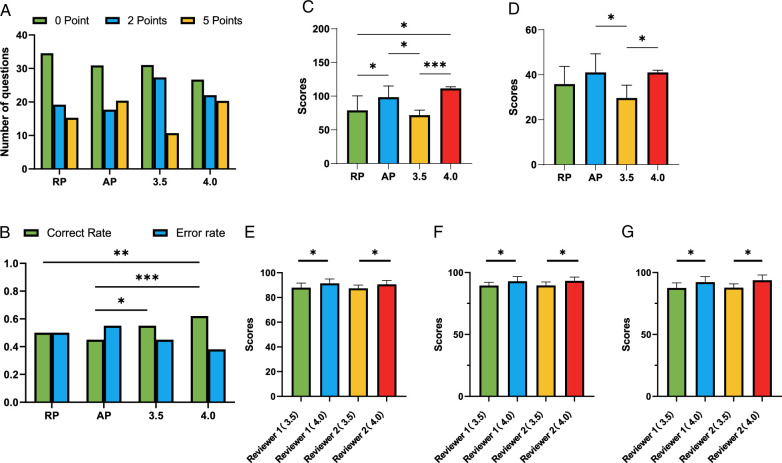
Assessment of artificial intelligence model performance versus clinician comparison group (A. Score rate, B. Correct rate, C. Case study issues, D. Indeterminate multiple-choice issues, E–G. Differences in psychological support capabilities of different AI large language models: urinary incontinence, erectile dysfunction, preoperative anxiety).

Details on score rates for each comparison group, percentage correct, and comparisons of case study and multiple-choice scores are presented in Figure 3A–D and Supplementary File 3, Supplemental Digital Content 4, http://links.lww.com/JS9/C996.

### Primary outcomes in psychological issues

#### Evaluation of artificial intelligence model performance regarding psychological support competencies

Combining the assessment results of both physician groups, the psychological support recommendations provided by ChatGPT, versions 3.5 and 4.0 were all deemed valid psychological support recommendations (>80 points). Based on an analysis of 10 incontinence-related psychological support issues, statistical results showed that ChatGPT 4.0 consistently outperformed ChatGPT 3.5, according to both reviewers, with a statistically significant difference in scores (*P*=0.044, *P*=0.030) (Fig. 3E). Based on an analysis of 10 erectile dysfunction-related psychological support issues, statistical results showed that ChatGPT 4.0 consistently outperformed ChatGPT 3.5, according to both reviewers, with a statistically significant difference in scores (*P*=0.036, *P*=0.014) (Fig. 3F). Based on the evaluation of 10 preoperative anxiety-related psychological support issues, statistical analysis demonstrated that ChatGPT 4.0 consistently scored higher than ChatGPT 3.5, according to both reviewers, with the difference in scores being statistically significant (*P*=0.026, *P*=0.002) (Fig. 3G). All statistical data for Figure 3E–G are shown in Supplementary Table 2 (Supplemental Digital Content 8, http://links.lww.com/JS9/C1000).

### Secondary outcomes in psychological issues

The responses of ChatGPT, versions 3.5 and 4.0 to the 32 clinical psychological guidance issues were individually evaluated by two clinical psychologists. The results are presented in Supplementary File 4, Supplemental Digital Content 9, http://links.lww.com/JS9/D2.

## Discussion

With the rapid advancement of AI technologies, the utilization of LLMs such as ChatGPT in the medical field has emerged as a prominent research topic^25,26^. The application of LLMs in the medical field not only demonstrates their potential for disease diagnosis, recommending treatment plans, and analyzing medical literature but also introduces novel perspectives and methods for shaping the future of healthcare services^27–29^. These AI capabilities offer viable solutions to contemporary challenges in the healthcare system, such as constrained resources and demanding workloads for doctors^30–34^. In this study, we initially discerned variances in the AI’s performance in clinical knowledge tests by comparing it with the actual performance of a group of physicians and assessed its ability to provide psychological support.

While AI excels in the diagnosis and treatment of most urologic diseases, its application in real clinical settings encounters significant limitations. These limitations primarily arise from the AI’s lack of expertise in handling complex data, specific medical domains, and the lack of real-world clinical experience^35–38^. Notably, the AI models exhibited significant disparities in treating different types of urological diseases. This could be attributed to insufficient pretraining data, resulting in knowledge gaps within the LLMs. Alternatively, the disparities may stem from setting fewer issues on certain disease types, leading to sample size bias in the results^39–41^. Examining Supplementary Figure 1, Supplemental Digital Content 6, http://links.lww.com/JS9/C998, it is evident that ChatGPT 4.0, as a representative of LLMs, exhibits several shortcomings. A simple statistical classification of pertinent topics reveals deficiencies in specific issues responses, such as staging and treatment of BC of different stages, diagnosis and treatment of complex prostatic hyperplasia, selection of intraoperative anesthesia modalities, specific scope of lymph node dissection for malignant tumors and their names, clinical manifestations of adrenocortical carcinoma, adjunctive investigations for complex urinary tract infections, definition of rare types of hydronephrosis, pheochromocytoma, biochemical monitoring after renal transplantation, surgical approach, and biochemical monitoring after renal transplantation. In these areas, ChatGPT 4.0 achieved unsatisfactory scores, registering zero. In contrast, the clinicians’ responses to these issues were not excellent but were at least deemed satisfactory. The abovementioned complex questions pose challenges even for senior urologists, let alone generic LLMs or junior physicians. However, this also underscores the need for innovative applications of LLMs, particularly in specialized disease models. As a generalized LLM, ChatGPT 4.0 is not yet ‘ready’ for clinical practice, despite offering some viable suggestions and maintaining a tolerable error rate.

ChatGPT has demonstrated some competence in providing psychological support^42^. Originally designed as ‘a chatbot’ the software serves its intended purpose well. In the three selected urological psychological application scenarios in this study, ChatGPT effectively offered psychological support suggestions. However, upon reviewing the contents of psychological advice from different ChatGPT versions, certain issues become apparent (Supplementary Table 3, Supplemental Digital Content 10, http://links.lww.com/JS9/D3). For example, the advice given in version 3.5 tends to be lengthy, with notable similarities between responses, probably resulting from limitations imposed by its pretrained dataset. Overall, AI models have the potential to be more comprehensive regarding psychological support compared with clinicians in clinical practice. Clinicians, often focused on diagnosing and treating diseases, may neglect to provide psychological guidance and comfort to patients, for example, in the treatment of erectile dysfunction. Faced with a substantial patient population, physicians may prioritize rational medication use or treatments^43,44^. In cases of psychogenic erectile dysfunction, psychological support is crucial, but it is often overlooked. However, AI models still have limitations in understanding emotions and providing in-depth psychological feedback. At present, AI models are not capable of thinking like a ‘human being’ within the current technological context.

This study represents an initial exploration that underscores the potential of AI technologies for supporting clinical decision-making and providing medical support. However, it also underscores the limitations of AI, particularly in navigating intricate clinical situations that demand profound expertise and empirical judgment. However, in the actual healthcare environment, the clinician’s job is much more than just providing treatment. A perfect patient–physician relationship requires a multidimensional assessment that includes patient–physician communication, patient cooperation, and the physician’s ability to put himself in the shoes of the patient to provide the ‘best’ rather than the ‘most expensive’ personalized treatment. This includes communication, patient cooperation, and the physician’s ability to put himself in the patient’s shoes and provide the ‘most appropriate’ rather than the ‘most expensive’ treatment. A procedure that simply offers ‘potential’ treatment options is not an ‘emotional and flesh-and-blood’ clinician, and patients, as a vulnerable group, often need a warm voice rather than cold texts. Therefore, at this stage, AI technologies are more suitable as an aid to physicians rather than independent decision makers. While there are reports of individuals relying on ChatGPT for medical diagnosis, there are also studies exhibiting that LLMs can generate misleading information that impacts the accuracy of physicians’ diagnoses^45–47^. Therefore, a physician’s experience, intuition, and expertise remain crucial in guiding AI through complex cases. The application of AI in real clinical settings requires a broader and more diverse set of real-world data to enhance its accuracy and adaptability in managing complex cases, indicating a considerable journey ahead.

This study has certain limitations. First, it relies heavily on predefined clinical questions and standardized tests, potentially limiting its ability to capture the complexity of real clinical settings fully. Moreover, the training data for AI models may not include all urologic diseases or adequately cover all relevant clinical variables. Second, while AI models can provide high-quality responses, the opacity of their internal decision-making process may pose challenges in interpretation. Finally, owing to the geographical limitation brought about by the fact that the scope of discussion of this study was limited to China, the results of ChatGPT/LLM’s applicability to the broad international medical arena are yet to be further explored. This is especially significant in the medical field, where clear logic and evidence support medical decisions.

## Conclusion

LLMs excel in handling standardized clinical medical problems and providing medical, psychological counseling. They enhance physicians’ ability to provide improved psychological support advice to patients, thus addressing a critical aspect often overlooked in the current healthcare landscape. However, considering the lack of clinical thinking abilities in AI models, potential misinformation in pretraining datasets that may mislead physician diagnoses and treatments, and the inability to provide refined and personalized decision-making recommendations, current LLMs represented by ChatGPT are better suited as auxiliary tools for clinicians to enhance diagnostic and therapeutic efficiency.

## Ethical approval

Not applicable.

## Consent

Not applicable.

## Source of funding

This work was supported by the National Natural Science Foundation of China: 82102171; National Natural Science Foundation of China (Tianyuan Fund for Mathematics): 12326610; Shenzhen Science and Technology Program: JCYJ2023080714100902, JCYJ20220818100015031; Shenzhen Medical Research Fund (SMRF): A2302048.

## Author contribution

S.W., S.Z., and S.Z,: study concept and design. R.S., R.L., A.Z.: data analysis and graphical beautification. R.L., L.P.: analysis and interpretation of data. L.P., R.L., and A.Z.: drafting of the manuscript. J.Z., F.Y., and R.L.: critical revision of the manuscript for important intellectual content. S.W., S.Z., S.Z., and L.P.: obtaining funding. S.Z.: administrative, technical, or material support. S.W.: supervision.

## Conflicts of interest disclosure

The authors declare no conflicts of interest.

## Research registration unique identifying number (UIN)

Not applicable.

## Guarantor

Song Wu.

## Data availability statement

All data generated and analyzed during this study are included in this published article. The data presented in the article may be requested by consulting the correspondence author.

## Provenance and peer review

Not commissioned, externally peer-reviewed.

## Supplementary Material

SUPPLEMENTARY MATERIAL
