# Supplementary material for: Amplifying Chinese physicians’ emphasis on patients’ psychological states beyond urologic diagnoses with ChatGPT – a multicenter cross-sectional study
Source: Int J Surg. 2024 Jul 2;110(10):6501–8. doi: 10.1097/JS9.0000000000001775 (PMC11487044; doi:10.1097/JS9.0000000000001775)
Supplement: SUPPLEMENTARY MATERIAL [file js9-110-6501-s002.docx]

**Supplementary Table 3**

**Amplifying Chinese Physicians' Emphasis on Patients' Psychological States Beyond Urologic Diagnoses with ChatGPT—A Multi-Center Cross-Sectional Study**

| **Supplementary Table 3. ChatGPT's demonstration of competence in psychological support.** | |
| --- | --- |
| 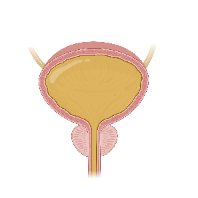 | **Patients with urinary incontinence are often depressed because of the shame they feel. How should the physician go about adjusting the patient's state of mind?** |
| 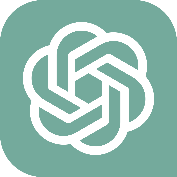 | 1. Establish a supportive, non-judgmental environment to build trust and encourage open communication.2. Educate the patient about urinary incontinence, its causes, and treatments to reduce anxiety and shame.3. Discuss personalized treatment options, including lifestyle changes, exercises, medications, or surgery.4. Refer to specialists like urologists or physical therapists for focused care in managing incontinence.5. Encourage self-care practices, including a healthy lifestyle, exercise, and balanced diet, to improve overall well-being.6. Suggest support groups or counseling for sharing experiences and reducing feelings of isolation.7. Address the emotional impact of incontinence by inquiring about mental health and providing relevant referrals.8. Offer behavioral therapies like CBT to help cope with and change negative thought patterns.9. Discuss the use of medications for symptom management, including benefits and side effects.10. Regularly monitor the patient's progress and provide ongoing emotional support.11. Empower the patient to take an active role in their treatment and management for improved self-esteem.12. Address body image concerns by offering resources or referrals to specialists for body confidence improvement. |
| 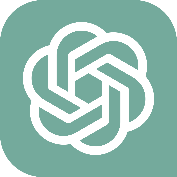 | 1. Engage in dialogue to understand and empathize with the patient's emotional responses, ensuring they feel understood and cared for. 2. Provide detailed information about the causes, treatments, and preventive measures for incontinence to alleviate anxiety and embarrassment. 3. Encourage patients to actively participate in their treatment, such as physical therapy and lifestyle changes, to improve symptoms. 4. Suggest joining support groups for sharing experiences with others facing similar incontinence challenges. 5. Recommend professional psychiatric or psychotherapeutic help, like CBT, for severe emotional issues related to incontinence. 6. Foster a positive outlook and self-acceptance, emphasizing that incontinence is not a personal fault or stigma. 7. Regularly monitor and adjust the treatment plan, ensuring the patient feels valued and cared for throughout their treatment journey. |
| 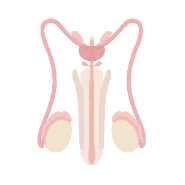 | **When male patients avoid having sex with their partners because of anxiety about sexual performance, how can physicians encourage them to confront this issue?** |
| 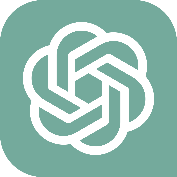 | When male patients avoid having sex with their partners due to anxiety about sexual performance, physicians can play a role in encouraging them to confront and address this issue. Here are some steps to help patients in this situation: 1. Create a safe, non-judgmental environment for discussing sexual health concerns.2. Initiate a gentle conversation about sexual health, using open-ended questions.3. Normalize performance anxiety as a common and manageable issue.4. Educate the patient about sexual health and factors contributing to performance anxiety.5. Explore the emotional effects of performance anxiety on personal and relational well-being.6. Help the patient identify specific triggers or patterns of their anxiety.7. Recommend individual or couple therapy, like CBT or sex therapy, for further support.8. Assess and address any underlying medical conditions affecting sexual performance.9. Emphasize the importance of open communication with partners about sexual concerns.10. Suggest couple's counseling to improve communication and understanding.11. Teach relaxation and mindfulness techniques to manage anxiety during sexual activity.12. Prescribe appropriate medication, if necessary, for anxiety and related symptoms.13. Encourage a gradual reintroduction to intimacy, starting with non-sexual forms.14. Assist in setting realistic expectations for sexual performance and intimacy.15. Provide ongoing support through regular follow-ups and adapt treatment plans as needed. |
| 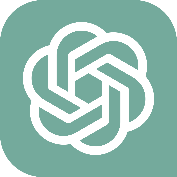 | When male patients avoid having sex with their partners because of anxiety about sexual performance, physicians can take the following steps to encourage them to confront the problem: 1. Have an in-depth discussion to fully understand the patient's anxiety and its specific causes.2. Show empathy and emotional support to make the patient feel respected and not judged.3. Explain that sexual performance anxiety is common and treatable like other health issues.4. Educate the patient about sexual health, functioning, and human physiology to dispel fears.5. Encourage patients to involve their partners in treatment for better transparency and trust.6. Discuss and offer appropriate treatment options, including psychotherapy, medication, or lifestyle changes.7. Teach patients stress management techniques like deep breathing and meditation.8. Set gradual, realistic goals to improve sexual function and build confidence.9. Refer the patient to a sex therapist or mental health professional when necessary.10. Support the patient's treatment progress with regular check-ups and positive reinforcement.11. Promote a holistic view of health, emphasizing that sexuality is one aspect of overall well-being. |
| **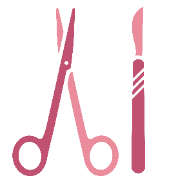** | **How should physicians advise and encourage patients when they refuse to leave their homes because they fear that a new urinary flow pattern may lead to leaks or accidents?** |
| 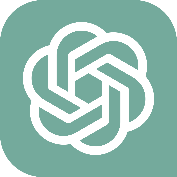 | 1. Initiate empathetic communication to explore and understand the patient's specific fears and anxieties. 2. Normalize the patient's concerns about urinary flow and the fear of accidents, explaining it's a common worry. 3. Provide education on the new urinary flow pattern, its functioning, and the adjustment process. 4. Discuss various management techniques and strategies, including bathroom schedules and protective measures. 5. Offer guidance on fluid intake and dietary choices to help manage urinary function and prevent accidents. 6. Encourage pelvic floor exercises or physical therapy to strengthen muscles and improve bladder control. 7. Suggest a gradual exposure approach to leaving the home, starting with short, familiar outings. 8. Consider referring the patient to a therapist for behavioral therapy to learn coping strategies and relaxation techniques. 9. Connect the patient with support groups or peers who have similar experiences for reassurance. 10. Discuss the use of medications or medical devices with a urologist or specialist for managing symptoms. 11. Schedule regular follow-up appointments for ongoing support and to monitor progress. 12. Respect the patient's autonomy and decision-making in managing their activities and healthcare. 13. Encourage involving a supportive network of friends or family for emotional support during outings. 14. Set realistic and achievable goals for gradually expanding activities outside the home and celebrate milestones. 15. Provide reassurance and hope that with time and management, confidence can improve and normalcy can return. |
| 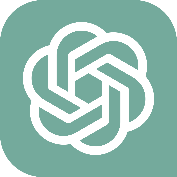 | 1. Educate patients in detail about how the new urinary flow modality works, common issues, and management techniques. 2. Provide skills training with hands-on demonstrations to help patients understand and master the new urinary flow modality. 3. Encourage patients to start with short trips close to home, gradually increasing in distance and duration to build confidence. 4. Help patients develop contingency plans, like carrying spare medical equipment and locating restrooms, for a sense of preparedness. 5. Offer psychological support through mental health professionals for patients with persistent anxiety. 6. Facilitate open communication between patients and their family and friends to ensure understanding and support. 7. Provide information on relevant products and services, such as absorbent devices, to minimize leakage concerns. 8. Conduct regular follow-ups to monitor progress and make adjustments to the patient's support and treatment plan. 9. Introduce patients to support groups or individuals in similar situations for social support and shared experiences. 10. Emphasize the importance of maintaining an active social life and daily activities for overall health and quality of life. |


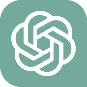
 ChatGPT 3.5
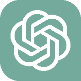
 ChatGPT 4.0
