# Supplementary material for: Amplifying Chinese physicians’ emphasis on patients’ psychological states beyond urologic diagnoses with ChatGPT – a multicenter cross-sectional study
Source: Int J Surg. 2024 Jul 2;110(10):6501–8. doi: 10.1097/JS9.0000000000001775 (PMC11487044; doi:10.1097/JS9.0000000000001775)
Supplement: SUPPLEMENTARY MATERIAL [file js9-110-6501-s003.docx]

**Supplementary file 1**

**Amplifying Chinese Physicians' Emphasis on Patients' Psychological States Beyond Urologic Diagnoses with ChatGPT—A Multi-Center Cross-Sectional Study**

**The following are additions to the "Methods" section.**

**Methods**

**Selection of Large Language Models** ChatGPT versions 3.5 and 4.0 were selected as resulting from their significant disparities in technical evolution and functional optimization. These versions represent distinct stages in the advancement of generative LLMs, rendering them suitable for assessing capabilities in addressing clinically and psychologically relevant questions [31-34].

**Population** The study comprised two primary groups: an experimental group consisting of the ChatGPT models and a control group of 25 urology specialists from eight prominent medical institutions in China. These clinicians, ranging from residents to attending physicians, engaged in a controlled question-and-answer session utilising a specialised ‘QR code’ applet (<https://www.wjx.cn/>). Each participating physician who completed all the questions received a shopping voucher worth RMB 100. Physicians were instructed to respond to all questions in a closed-book format within a 2-hour timeframe, and the response process was unidirectional, preventing participants from revisiting and altering answers to previously addressed questions. Individual scores were not immediately accessible to doctors after submission; instead, all scores were aggregated to the backend of the program for the experimenter's review.

**Clinical Issues**

The issues were structured as multiple-choice questions with no definite answer; each question presented multiple alternatives, and the correct answer could be one or more of them. Participants selected the answer they deemed correct based on their knowledge base. The total mark for the assessment was set at 345, with individual issues worth 5 points, 2 points deducted for under-selection, and 0 points for selecting any incorrect answer.
