# Supplementary material for: Amplifying Chinese physicians’ emphasis on patients’ psychological states beyond urologic diagnoses with ChatGPT – a multicenter cross-sectional study
Source: Int J Surg. 2024 Jul 2;110(10):6501–8. doi: 10.1097/JS9.0000000000001775 (PMC11487044; doi:10.1097/JS9.0000000000001775)
Supplement: SUPPLEMENTARY MATERIAL [file js9-110-6501-s004.docx]

**Supplementary file 2**

**Amplifying Chinese Physicians' Emphasis on Patients' Psychological States Beyond Urologic Diagnoses with ChatGPT—A Multi-Center Cross-Sectional Study**

**The following are Urology Clinical Knowledge Topic Design and Answers. The red text represents the reference answer to the question.**

1. History summary: The patient is a male, 67 years old, with dysuria for 1 year, nocturia 3 times, accompanied by frequent urination. There was no urinary pain or hematuria. History of diabetes mellitus, hypertension and cerebrovascular accident was denied. Physical examination: T36.6°C, P88 beats/min, R20 beats/min, BP130/70 mm Hg.Which examination should not be done before treatment?

A International Prostate Symptom Score (IPSS)

B Quality of life assessment (QoL)

C Serum prostate-specific antigen (PSA)

D Free urine flow rate

E Ultrasound residual urine output measurement

F Bladder Pressure Volume Measurement

G Pressure-flow rate measurement

H Static urethral pressure tracing

I Urine routine

2. Abstract: The patient is a 67-year-old male with dysuria for 1 year, nocturia 3 times, accompanied by frequent urination. There was no urinary pain or hematuria. History of diabetes mellitus, hypertension and cerebrovascular accident was denied. Physical examination: T36.6°C, P88 beats/min, R20 beats/min, BP130/70mmHg. What further investigations should be performed? Prompts: IPSS=18 points, QoL=3 points, PSA=3.0ng/ml, maximal urinary flow rate=14ml/s, residual urine volume=15ml. urinary routine is normal.

A Ultrasound of the prostate

B Prostate CT

C Intravenous urography (IVU)

D Prostate aspiration biopsy

E Cystoscopy

F Rectal palpation

3. Abstract: The patient is a 67-year-old male with dysuria for 1 year, nocturia 3 times, accompanied by frequent urination. There was no urinary pain or hematuria. History of diabetes mellitus, hypertension and cerebrovascular accident was denied. Physical examination: T36.6°C, P88 beats/min, R20 beats/min, BP130/70 mm Hg. What diagnosis is not being considered? Hint: Rectal palpation: enlarged prostate, shallow central sulcus, no hard nodules detected. Ultrasound of the prostate: 50 ml of prostate, no hypoechoic areas seen in the outer glandular region.
A Chronic prostatitis
B Prostate cancer
C Benign prostatic hyperplasia
D Benign prostatic hyperplasia with prostate cancer.
E Diagnosis not clear, need further examination

4. Abstract: The patient is a 67-year-old male with dysuria for 1 year, nocturia 3 times, accompanied by frequent urination. There was no urinary pain or hematuria. History of diabetes mellitus, hypertension and cerebrovascular accident was denied. Physical examination: T36.6°C, P88 beats/min, R20 beats/min, BP130/70 mm Hg. What is the treatment of choice?

A Tamsulosin hydrochloride

B Probenecid

C Cordova in combination with povidonexin

D Comstock E Transurethral resection of the prostate (TURP)

F Suprapubic prostatectomy

G Finasteride

5. Abstract: The patient is a 67-year-old male with dysuria for 1 year, nocturia 3 times, accompanied by frequent urination. There was no urinary pain or hematuria. History of diabetes mellitus, hypertension and cerebrovascular accident was denied. Physical examination: T36.6°C, P88 beats/min, R20 beats/min, BP130/70 mm Hg. What further investigations can be performed? Tip: After six months of treatment with Cordovan and Polyjuice, there was no improvement in symptoms. The patient strongly urges surgery.

A Cystography

B Pressure-flow rate measurement

C Bladder pressure-volume measurement

D Abdominal leakage point manometry

E Leakage point manometry of the detrusor muscle

F IVU

G Ultrasound of the urinary tract

6. Abstract: The patient is a 67-year-old male with dysuria for 1 year, nocturia 3 times, accompanied by frequent urination. There was no urinary pain or hematuria. History of diabetes mellitus, hypertension and cerebrovascular accident was denied. Physical examination: T36.6°C, P88 beats/min, R20 beats/min, BP130/70 mm Hg. Which treatment is most appropriate? Hint: Urodynamic examination suggests no bladder outlet obstruction.IVU and urinary ultrasound show no abnormalities.

A Transurethral resection of the prostate (TURP)

B Suprapubic prostatectomy

C Radiofrequency therapy of the prostate

D Replacement of alpha-blocker

E Watchful waiting

7. Abstract: The patient is a 67-year-old male with dysuria for 1 year, nocturia 3 times, accompanied by frequent urination. There was no urinary pain or hematuria. History of diabetes mellitus, hypertension and cerebrovascular accident was denied. Physical examination: T36.6°C, P88 beats/min, R20 beats/min, BP130/70 mmHg. How should the treatment plan be adjusted? Tip: After changing the α-blocker, the symptoms of dysuria improved significantly, but the symptoms of urinary frequency and nocturia did not change.

A Increase the α-blocker dose.

B Increase the dose of tolterodine.

C Increase the dose of probenecid

D Increase the dose of cardioplegia

E Add pyridostigmine

F Add tachycardia

8. History Summary: The patient is a 38-year-old male with intermittent painless full-length hematuria of the flesh for 1 day. The hematuria was washout-like, without clots. There was no urinary frequency, urgency or pain. No straining to urinate. No back pain. Healthy in general. Physical examination: no percussion pain in both kidneys, no tenderness in bilateral ureteral stretches, and no distension or tenderness in the suprapubic area. Which is the most likely diagnosis?

A Urinary tract infection

B Bladder stones

C Bladder cancer

D Ureteral cancer

E Cancer of the renal pelvis

F Kidney Cancer

G Bladder Tuberculosis

H Ureteral polyp

9. History Summary: The patient is a 38-year-old male with intermittent painless full-length hematuria of the flesh for 1 day. The hematuria was washout-like, without clots. There was no urinary frequency, urgency or pain. No straining to urinate. No back pain. Healthy in general. Physical examination: no percussion pain in both kidneys, no tenderness in bilateral ureteral stretches, no bulging or tenderness in the suprapubic area. What is not a preferred screening test?

A Renal ECT

B isotope bone scan

C Urine cytology

D Pelvic CT

E Pelvic MRI

F Ultrasound of the urinary tract

10. History Summary: The patient is a 38-year-old male with intermittent painless full-length hematuria of the flesh for 1 day. The hematuria was washout-like with no clots. There was no urinary frequency, urgency or pain. No urinary effort. No lumbar pain. Healthy in general. Physical examination: no percussion pain in both renal regions, no pressure pain in bilateral ureteral trips, no bulging or pressure pain in the suprapubic region. What tests do not make a definitive diagnosis? Suggestive: no cancer cells seen on 3 consecutive urine cytology tests. ultrasound suggests: a 2 cm sized occupancy in the right wall of the bladder, with no visible tibia, which does not move with positional changes.

A IVU

B Cystoscopy + biopsy

C Pelvic CT

D Pelvic MRI

E Isotope bone scan

F Renal ECT

11. History Summary: The patient is a 38-year-old male with intermittent painless full-length hematuria of the flesh for 1 day. The hematuria was washout-like with no clots. There was no urinary frequency, urgency or pain. No urinary effort. No lumbar pain. Healthy in general. Physical examination: no percussion pain in both renal regions, no pressure pain in bilateral ureteral trips, no bulging and pressure pain in the suprapubic region. What is the clinical stage of this case of bladder migratory cell carcinoma? Hint: Cystoscopy showed a coral-like mass on the right wall near the ureteral orifice, light orange-red in color, about 2 cm in size, with a short tip, and no abnormality on the remaining bladder walls. The pathology report of the mass biopsy was grade II migratory cell carcinoma.A small filling defect on the left side of the bladder was seen vaguely on IVU, and the rest of the bladder showed no abnormality.

A Further pelvic CT is needed to determine this.

B Further pelvic MRI is required to confirm this.

C Further isotope bone scanning is required to confirm

D Tis

E Ta

F T2aN0M0

G T1N0M0

H T2N0M0

I T3aN0M0

12. History Summary: The patient is a 38-year-old male with intermittent painless full-length hematuria of the flesh for 1 day. The hematuria was washout-like with no clots. There was no urinary frequency, urgency or pain. No urinary effort. No lumbar pain. Healthy in general. Physical examination: no percussion pain in both renal regions, no pressure in bilateral ureteral trips, no bulging or pressure in the suprapubic region. Which treatment measure is most appropriate?

A Radical cystectomy

B radical cystectomy + ileal cystectomy

C radical cystectomy + in situ ileocecal neobladder

D Transurethral electrolysis of bladder tumor (TURBt)

E Bladder instillation of BCG

F Bladder instillation of mitomycin

G Partial bladder resection

H TURBt + bladder infusion of mitomycin

I Partial cystectomy + bladder infusion of mitomycin

13. History Summary: The patient is a 38-year-old male with intermittent painless full-length hematuria of the flesh for 1 day. The hematuria was washout-like with no clots. There was no urinary frequency, urgency or pain. No urinary effort. No lumbar pain. Healthy in general. Physical examination: no percussion pain in both renal regions, no pressure pain in bilateral ureteral trips, no bulging and pressure pain in the suprapubic region. Which anesthesia would be the most effective to choose to avoid occlusive nerve reflexes in this case of proposed transurethral cystectomy for bladder tumor?

A Epidural anesthesia

B Epidural anesthesia + right obturator nerve block

C Epidural anesthesia + bilateral obturator nerve block

D Sacral anesthesia + right occlusive nerve block

E Sacral anesthesia + bilateral obturator nerve block

F Lumbar anesthesia + right occlusive nerve block

G Lumbar anesthesia + bilateral occlusive nerve block

H General anesthesia

I General anesthesia + right sided obturator nerve block

J General anesthesia + bilateral obturator nerve block

14. History Summary: The patient is a 38-year-old male with intermittent painless full-length hematuria of the flesh for 1 day. The hematuria was washout-like with no clots. There was no urinary frequency, urgency or pain. No urinary effort. No lumbar pain. Healthy in general. Physical examination: no percussion pain in both renal regions, no pressure pain in bilateral ureteral trips, no bulging and pressure pain in the suprapubic region. In this case, a transurethral cystectomy for bladder tumor is proposed. Which intraoperative bladder irrigation solution is preferred?

A 0.9% saline

B 5% dextrose

C 10% dextrose

D Distilled water

E 10% mannitol

F 20% mannitol

15. History Summary: The patient is a 38-year-old male with intermittent painless full-length hematuria of the flesh for 1 day. The hematuria was washout-like with no clots. There was no urinary frequency, urgency or pain. No urinary effort. No lumbar pain. Healthy in general. Physical examination: no percussion pain in both renal regions, no pressure in bilateral ureteral trips, no bulging or pressure in the suprapubic region. When is the best time to start bladder instillation of mitomycin?

A At the end of surgery

B 1 week postoperatively

C 2 weeks postoperatively

D 3 weeks postoperatively

E 4 weeks postoperatively

16. Detailed reading of the abstract of the medical record, the following characteristics can be summarized: ① elderly male patients; ② the main complaint of urinary abnormalities, the prominent one is urinary frequency, which gradually develops into difficulty in urination, so that urination does not go out for 16 hours; ③ accompanied by the phenomenon of infection; ④ there is no history of hematuria and trauma; ⑤ there is a history of hypertension for 10 years; ⑥ physical examination has a high blood pressure, the bladder area is bulging, and the skin is dry and poor elasticity; ③ emergency medical consultation at 22 hours. Which examination should preferably be performed first in an emergency?

A Rectal palpation for prostate after emptying the bladder

B Rectal palpation first to visualize the prostate as soon as possible

C Electrocardiogram

D Serum potassium, sodium and chloride

E Serum creatine phosphokinase, lactate dehydrogenase, prostatic acid phosphatase

F Renogram

G Drainage urine culture for pathogenic bacteria, bacterial count and drug sensitivity test

H Drainage urine smear for Gram staining

17. Detailed reading of the abstract of the medical record, the following characteristics can be summarized: ① elderly male patient; ② the main complaint of urinary abnormalities, the prominent one is urinary frequency, which gradually develops into difficulty in urination, so that urination does not go out for 16 hours; ③ accompanied by the phenomenon of infection; ④ there is no history of hematuria and history of trauma; ⑤ there is a history of hypertension for 10 years; ⑥ physical examination there is a high blood pressure, the bladder area is bulging, the skin is dry and poor elasticity; ③ emergency room at 22:00 hours to seek medical attention. What should be done as soon as possible in the emergency room?

A Catheterization followed by indwelling catheter

B No catheter left in place after catheterization

C Intramuscular atropine

D Intramuscular neostigmine

E Intramuscular tachycardia

F Intramuscular sedation

18. Reading the abstract in detail, the following features can be summarized: ① elderly male patient; ② abnormal urination as the main complaint, the prominent one is frequent urination, which gradually develops into difficulty in urination to the point of not urinating for 16 hours; ③ accompanied by the phenomenon of infection; ④ no history of hematuria and trauma; ⑤ a history of hypertension for 10 years; ⑥ physical examination with high blood pressure, bladder area bulge, dry and poor elasticity of the skin; ③ emergency room visit at 22 hours. What are the main diseases to be considered in the initial diagnosis? Blood leukocytes 13.0 × 10／L, neutrophils 0.78, chest radiographs show a still large heart shadow.

A Acute prostatitis with urinary retention

B Prostatic hyperplasia with infection and urinary retention

C Prostatic hyperplasia with a stone in the posterior urethra.

D Posterior urethral stone impaction with urinary retention

E Urethral stricture

F Coronary heart disease

G Hypertensive heart disease

19. Detailed reading of the abstract of the medical record, the following characteristics can be summarized: ① elderly male patient; ② abnormal urination as the main complaint, highlighting the frequency of urination, which gradually developed into dysuria, to the point of urination for 16 hours; ③ accompanied by the phenomenon of infection; ④ without a history of hematuria and history of trauma; ⑤ history of hypertension for 10 years; ⑥ physical examination has a high blood pressure, the bladder area is bulging, the skin is dry, poor elasticity; ③ 22 hours of emergency room to the doctor. What further investigations should be performed based on the above? Rectal palpation reveals loss of the central sulcus of the prostate with enlarged lobes on both sides. The texture is hard.

A Excretory urography

B Blood sedimentation

C Abdominal plain film

D Serum urea nitrogen and creatinine

E Cystoscopy and retrograde pyelogram

F Renogram

G Ultrasound of both kidneys, bladder and prostate

H Blood prostate acid phosphatase and prostate specific antigen (PSA)

20. Reading the abstract in detail, the following characteristics can be summarized: ① elderly male patient; ② abnormal urination as the main complaint, the prominent one is urinary frequency, which gradually develops into difficulty in urination to the point of urinating for 16 hours; ③ accompanied by the phenomenon of infection; ④ there is no history of hematuria and history of trauma; ⑤ there is a history of hypertension for 10 years; ⑥ physical examination has a high blood pressure, the bladder area is bulging, and the skin is dry and poor elasticity; ③ the patient seeks emergency medical treatment at 22:00 hours. Which of the following tests in this patient would provide further insight into cardiac function?

A Phosphocreatine kinase, glutamate aminotransferase, lactate dehydrogenase

B Lipids

C cardiac ultrasound

D cardiac blood pool imaging

E Cardiopulmonary impedance mapping

F Cardiac systolic time interval measurement (STI)

G Bicycle test

H Chest X-ray

I Cold compression test

J Myocardial imaging

21. Detailed reading of the medical history summary, the following characteristics can be summarized: ① elderly male patient; ② abnormal urination as the main complaint, the prominent one is urinary frequency, which gradually develops into difficulty in urination to the point of not urinating for 16 hours; ③ accompanied by the phenomenon of infection; ④ no history of hematuria and trauma; ⑤ a history of hypertension for 10 years; ⑥ physical examination with high blood pressure, bladder area bulge, dry and poor elasticity of the skin; ③ emergency room visit at 22 hours. Based on the above findings, what diagnosis is considered in conjunction with the history? Since four days of admission, he was poor in spirit, did not think about food and drink, and had a 24-hour urine output of 780 ml. Hb120g/L, blood urea nitrogen 15.0mmol/L (42mg/dl), and blood creatinine 221μmol/L (2.5mg/dl). Urine culture had Aspergillus growth, >100×10 colonies/L (>100×10 colonies/ml). Blood carbon dioxide partial pressure was 21.1 mmol/L (47 ml%). Blood pH 7.33, BE-7mmol/L, SB19mmol/L, PCO 3.7kPa (28mmHg).

A Metabolic acidosis

B Compensated respiratory alkalosis

C Respiratory acidosis

D Respiratory alkalosis and compensatory acidosis

E Prostatic hyperplasia

F Compensated renal insufficiency

G Azotemia

H Uremia

I Pyelonephritis

J Acute cystitis

22. Detailed reading of the abstract of the medical record, the following characteristics can be summarized: ① elderly male patient; ② abnormal urination as the main complaint, the prominent one is urinary frequency, which gradually develops into difficulty in urination, so much so that urination does not occur for 16 hours; ③ accompanied by the phenomenon of infection; ④ there is no history of hematuria and history of trauma; ⑤ there is a history of hypertension for 10 years; ⑥ physical examination there is a high blood pressure, the bladder area is bulging, the skin is dry and poor elasticity; ③ emergency room at 22 times to seek medical attention. What are the main reasons for the above conditions? Since four days of admission, he was poor in spirit, did not think about food and drink, and had a 24-hour urine output of 780 ml. Hb120g/L, blood urea nitrogen 15.0mmol/L (42mg/dl), and blood creatinine 221μmol/L (2.5mg/dl). Urine culture had Aspergillus growth, >100×10 colonies/L (>100×10 colonies/ml). Blood carbon dioxide partial pressure was 21.1 mmol/L (47 ml%). Blood pH 7.33, BE-7 mmol/L, SB19 mmol/L, PCO3.7 kPa (28 mmHg).

A Resulting from renal impairment due to prolonged lower urinary tract obstruction

B Inadequate water and electrolyte intake after disease onset

C Excessive loss of sodium from the urine

D Pyelonephritis due to uncontrolled lower urinary tract infection

E Failure to use diuretics

F Renal impairment due to hypertension

G Due to cardiac hypoplasia

23. Reading the abstract in detail, the following characteristics can be summarized: ① elderly male patient; ② abnormal urination as the main complaint, the prominent one is frequent urination, which gradually develops into difficulty in urination to the point of not urinating for 16 hours; ③ accompanied by the phenomenon of infection; ④ there is no history of hematuria and history of trauma; ⑤ there is a history of hypertension for 10 years; ⑥ physical examination has a high blood pressure, the bladder area is bulging, the skin is dry and poor elasticity; ③ at 22 times the emergency room visits the doctor. Which of the following is an indication for surgery for prostatic hyperplasia? Proposed surgical treatment.

A Frequent urination and difficulty in urination are still obvious after medication, affecting night rest.

B A hard nodule is found on prostate examination, and prophylactic resection of the prostate is performed to prevent cancer.

C Recurrent urinary retention

D Dilatation of the upper urinary tract leading to renal impairment.

E Residual urine volume of 40 ml

F Depends on ultrasound to determine the size of the prostate.

G Prostatic hyperplasia combined with bladder stones

24. The patient is a female, 45 years old. Paroxysmal dizziness for 2 years, sometimes accompanied by sudden onset of precordial discomfort, accompanied by pallor, dyspnea, profuse sweating, blood pressure up to 200/120mmHg during the attack. weight loss of about 8kg over the past two years. normal diet, normal bowel movements. Physical examination: T:37.2℃, P:100 beats/min, BP:150/100mmHg. abdomen is flat. What imaging tests should be performed to help diagnose

A Ultrasound of liver, gallbladder, spleen and pancreas

B Peri-abdominal aortic and pelvic ultrasound.

C Bilateral adrenal ultrasound

D IVP

E CTU

F KUB

G Bilateral adrenal CT

H MRU

I Beta-iodobenzylguanidine assay

25. The patient is a female, 45 years old. Paroxysmal dizziness for 2 years, sometimes accompanied by sudden onset of precordial discomfort, accompanied by pallor, dyspnea, profuse sweating, blood pressure up to 200/120mmHg during the attack. weight loss of about 8kg over the past two years. diet is normal, bowel movement is normal. Physical examination: T:37.2℃, P:100 beats/min, BP:150/100mmHg. abdomen flat. ultrasound suggests left adrenal region -4cm×5cm spherical mass, internal echogenicity is uneven. ct suggests left adrenal region 4cm×4.5cm parenchymal space-occupying lesion, border smooth and adrenal gland closely connected, border clear, internal has uneven enhancement. In order to establish the diagnosis, it would be reasonable to perform the following tests

A Electrocardiogram

B Lung function

C Thyroid function tests

D Blood gas analysis

E 24-hour urine CA and VMA measurements

F Plasma Catecholamines

G 24-Hour Urine Potassium

H 24-Hour Urine 17,Hydroxy and 17 Keto Steroids Measurement

26. The patient is a female, 45 years old. Paroxysmal dizziness for 2 years, sometimes accompanied by sudden onset of precordial discomfort, accompanied by pallor, dyspnea, profuse sweating, blood pressure up to 200/120mmHg during the attack. weight loss of about 8kg over the past two years. normal diet, normal bowel movements. Physical examination: T:37.2℃, P:100 beats/min, BP:150/100mmHg. abdomen flat. 24-hour urine CA and VMA higher than the upper limit of normal 2 times. ECG: sinus rhythm, 100 beats/min, II, III, AVF ST segment downshift. The probable diagnosis for this patient should be

A Primary hypertension

B Secondary hypertension

C left adrenocortical tumor

D left adrenal medullary tumor

E Catecholamines

F Pheochromocytoma

G Cardiac arrhythmia

27. The patient is a female, 45 years old. Paroxysmal dizziness for 2 years, sometimes accompanied by sudden onset of precordial discomfort, accompanied by pallor, dyspnea, profuse sweating, blood pressure up to 200/120mmHg during the attack. weight loss of about 8kg over the past two years. normal diet, normal bowel movements. Physical examination: T:37.2°C, P:100 beats/min, BP:150/100mmHg. abdomen is flat. The treatment options for this patient are

A α-blocker to control blood pressure

B open left adrenal tumor resection

C aminoglycosaminoglycan injection therapy

D Laparoscopic left adrenal tumor resection

E Ultrasound-guided left adrenal tumor ablation

F Watchful waiting

28. The patient is a female, 45 years old. Paroxysmal dizziness for 2 years, sometimes accompanied by sudden onset of precordial discomfort, accompanied by pallor, dyspnea, profuse sweating, blood pressure up to 200/120mmHg during the attack. weight loss of about 8kg over the past two years. normal diet, normal bowel movements. Physical examination: T:37.2°C, P:100 beats/min, BP:150/100mmHg. flat abdomen. The patient's perioperative treatment is correct.

A Blood pressure can be controlled with alpha-blockers

B Sodium nitroprusside is the drug of choice for the treatment of paroxysmal blood pressure increase

C sinus tachycardia can be treated with cardiac glycosides.

D A small amount of intraoperative blood reserve

E Blood volume should not be supplemented before surgery because of the patient's high basal blood pressure.

F Preoperative patients should not be strenuously active

G Intraoperative central venous pressure should be monitored

29. A 67-year-old male with an asymptomatic 3-cm strongly echogenic lesion in the lower and middle pole of the right kidney was found on ultrasound 2 weeks ago. In order to further clarify the diagnosis, the patient can choose which of the following examinations

A CT scan of both kidneys

B renal enhancement CT of both kidneys

C MRI enhancement of both kidneys

D IVP

E MRU

F Cystoscopy

30. The patient is a 67-year-old male. 2 weeks ago, ultrasound examination revealed a 3-cm strongly echogenic space-occupying lesion in the middle and lower pole of the right kidney, with outward growth and asymptomatic. The possible diagnosis is

A renal malformation tumor

B renal pelvis cancer

C renal carcinoma

D renal tuberculosis

E renal cyst

F Renal lipoma

31. The patient is a male, 67 years old. 2 weeks ago ultrasound examination revealed a 3 cm partial echogenic space-occupying lesion in the middle and lower pole of the right kidney, with outward growth, and asymptomatic. Treatment of this patient may be

A Renal, perinephric fat capsule and hilar lymph node resection together

B cuff-like resection of the kidney, ureter and part of the bladder

C nephrectomy alone

D Pharmacologic antituberculosis therapy

E Observation and follow-up

F Partial nephrectomy with preservation of renal units

32. The patient is a 72-year-old male with intermittent full-length painless hematuria for 2 months. Ultrasound shows no hydronephrosis in both kidneys or ureters, and a 2.2 cm × 1.0 cm isoechoic mass in the left wall of the bladder, protruding into the bladder lumen. The next step in the workup should be

A Cystoscopy with biopsy of the mass

B Pelvic CT scan with enhancement

C IVP

D transrectal prostate ultrasound

E Urodynamics

F Bilateral renal CT scan with enhancement

G Whole body PET

33. The patient was a 72-year-old male with intermittent full-length painless hematuria for 2 months. Ultrasound showed no hydronephrosis in both kidneys and ureters, and a 2.2cm x 1.0cm isoechoic mass in the left wall of the bladder, protruding into the bladder cavity. Cystoscopy showed a ca. 2cmxlcm cauliflower-like mass outside and above the left ureteral orifice on the left wall of the bladder, with a tip, and a biopsy of the mass was given after electrocautery, with no lesions seen in the remaining bladder. Pathologic return of biopsy: low-grade uroepithelial carcinoma of the bladder.

A Pelvic radiotherapy

B systemic chemotherapy

C local bladder perfusion chemotherapy

D Watchful waiting

E Immunotherapy

F Endocrine therapy

34. The patient is a 72-year-old male with intermittent full-length painless hematuria of the flesh for 2 months. Ultrasound shows no hydronephrosis in both kidneys or ureters, and an isoechoic mass of 2.2 cm × 1.0 cm in the left wall of the bladder, protruding into the bladder cavity. The patient's time for repeat cystoscopy should be

A Two weeks

B one month

C two months

D three months

E Six months

F Nine months

G One year

35. Male, 30 years old, 3 months after kidney transplantation, has been oral cyclosporine, primidone, prednisone treatment, renal function is good, today morning the patient appeared to have a fever, body temperature 38 ℃, urine output is normal, come to the hospital in order to clarify the diagnosis of the need to do which examination

A blood routine, biochemistry

B Chest X-ray

C color ultrasound of transplanted kidney

D Cyclosporine concentration

E Urine routine

36. Male, 30 years old, 3 months after kidney transplantation, has been taking oral cyclosporine, primaquine, prednisone treatment, renal function is good, this morning the patient appeared to have fever, body temperature 38 ℃, urine output is normal, come to the hospital chest X-ray suggests that the scattered patchy shadow of both lungs, the blood leukocytes are 12 × 10 /L, cyclosporine concentration of 380 ng / ml, the next step of the examination choice

A Blood CMVIgM test

B. Sputum bacterial culture

C. Sputum smear

D. Sputum fungal culture

E Lung function

37. Male, 30 years old, 3 months after kidney transplantation, has been taking oral cyclosporine, primidone, prednisone treatment, renal function is good, today morning the patient appeared fever, body temperature 38 ℃, urine output is normal, come to the hospital to see the examination results suggestive of CMVIgM (±), sputum culture did not see bacterial and fungal growth, the next step of the most meaningful treatment is

A antibiotics

B antifungal drugs

C antiviral ganciclovir

D anti-TB treatment

E None of the above

38. Male, 30 years old, 3 months after renal transplantation, has been oral cyclosporine, primidone, prednisone treatment, renal function is good, this morning the patient appeared to have a fever, body temperature 38 ℃, urine output is normal, come to the hospital treatment process of immunosuppressive drugs have meaningful adjustments are

A increase cyclosporine, primidone and prednisone dosage

B Increase the dosage of cyclosporine, discontinue primidone, and leave prednisone unchanged.

C Decrease cyclosporine dosage and leave primidone and prednisone unchanged.

D Decrease cyclosporine dosage, discontinue primidone, and increase prednisone dosage appropriately

E Reduce cyclosporine, primidone and prednisone.

39. Male, 32 years old. Frequent urination, urinary urgency, painful urination for 1.5 years. Symptoms recurring and gradually aggravated, conventional anti-infective treatment was not effective. In the last 2 months, there were repeated episodes of cloudy urine and terminal hematuria, bladder irritation symptoms worsened, accompanied by nausea, low-grade fever, and left lumbar distension. According to the patient's condition, the initial diagnosis is first considered

A Urinary system tumor

B urinary system tuberculosis

C acute attack of chronic prostatitis

D Upper left urinary tract stone with infection

E Chronic pyelonephritis

F Chronic interstitial cystitis

40. Male, 32 years old. Frequent urination, urinary urgency, painful urination for 1.5 years. Symptoms recurring and gradually aggravated, conventional anti-infective treatment was not effective. In the last 2 months, recurrent cloudy urine and terminal hematuria, bladder irritation symptoms worsened, accompanied by nausea, low-grade fever, left lumbar distension and pain. The current investigations to be performed are

A Intravenous pyelogram

B urinary angiography

C Urine routine

D Bacterial culture of mid-stream urine + drug sensitivity

E Urine sediment for antacids

F Cystoscopy

41. Male, 32 years old. Frequent urination, urinary urgency, painful urination for 1.5 years. Symptoms recurring and gradually aggravated, conventional anti-infective treatment was not effective. In the last 2 months, recurrent cloudy urine and terminal hematuria, bladder irritation symptoms worsened, accompanied by nausea, low-grade fever, left lumbar distension and pain. Based on the clinical diagnosis, the primary therapeutic measure is (Hint: Antacids were found on examination. The right kidney function is normal without fluid accumulation. The left renal collecting system was severely disrupted, with multiple narrowing and dilatation of the left ureter. Bladder capacity is normal).

A Antituberculosis treatment

B Systemic supportive therapy

C Correction of malnutrition and hypoproteinemia

D Symptomatic treatment

D Symptomatic treatment E Combined treatment with Chinese and Western medicine

F Early surgical treatment

42. Male, 32 years old. Frequent urination, urinary urgency, painful urination for 1.5 years. Symptoms recurring and gradually aggravated, conventional anti-infective treatment was not effective. In the last 2 months, recurrent cloudy urine and terminal hematuria, bladder irritation symptoms worsened, accompanied by nausea, low-grade fever, and left-sided lumbar distension and pain. The next treatment plan should be taken (Hint: After 2 weeks of treatment, the patient's general condition has improved and clinical symptoms have significantly decreased).

A Continue anti-tuberculosis treatment

B Total left nephrectomy

C Total resection of the left kidney and ureter

D Total left nephrectomy and ureteral resection, and continue anti-tuberculosis treatment after surgery.

E Left nephrostomy

F Left nephrostomy with ureteral dilatation

43. Male, 77 years old. He was hospitalized for six months because of frequent urination, uncontrollable mainstream urination during activities and nocturnal enuresis. History: 5 years ago, the symptoms of thinning of the urinary line and waiting for urination appeared, and gradually aggravated. Physical examination: general condition was good. The base of the bladder was located 8cm above the pubic bone on percussion. dactyloscopy: prostate hyperplasia of the third degree, urine routine: WBC (++++), RBC (+). The patient's urinary incontinence should be classified as

A True stress incontinence

B complete urethral insufficiency

C Urge incontinence

D mixed urge/stress incontinence

E Filling incontinence

F Sphincter dyssynergia

44. Male, 77 years old. He was hospitalized for six months because of frequent urination, uncontrollable mainstream urination during activities and nocturnal enuresis. History: 5 years ago, the symptoms of thinning of the urinary line and waiting for urination appeared, and gradually aggravated. Physical examination: general condition was good. The base of the bladder was located 8cm above the pubic bone on percussion. dactyloscopy: prostate hyperplasia of the third degree, urine routine: WBC (++++), RBC (+). Blood creatinine is 155 μmol/L. The correct interpretation is (Suggestion: urodynamic examination suggests bladder outlet obstruction, 500 ml of residual urine, increased bladder compliance, and low and flat cystometric curves in both the storage and voiding phases).

A Bladder outlet obstruction bladder dyssynergia

B High-pressure acute urinary retention

C High-pressure chronic urinary retention

D Low-pressure acute urinary retention

E Low-pressure chronic urinary retention

F Bladder outlet obstruction bladder compensation

45. Male, 77 years old. He was hospitalized for six months because of frequent urination, uncontrollable mainstream urination during activities and nocturnal enuresis. History: 5 years ago, the symptoms of thinning of the urinary line and waiting for urination appeared, and gradually aggravated. Physical examination: general condition was good. The base of the bladder was located 8cm above the pubic bone on percussion. dactyloscopy: prostate hyperplasia of the third degree, urine routine: WBC (++++), RBC (+). Blood creatinine is 155 μmol/L. This type of incontinence is characterized by

A Most of them have bladder hyperalgesia.

B large volume bladder

C mostly high-compliance bladder

D mostly low-compliance bladder

E Significant decrease in both mean and maximal urinary flow rates

F Mean and maximal urinary flow rates are markedly elevated

46. Male, 77 years old. He was hospitalized for six months because of frequent urination, uncontrollable mainstream urination during activities and nocturnal enuresis. History: 5 years ago, the symptoms of thinning of the urinary line and waiting for urination appeared, and gradually aggravated. Physical examination: general condition was good. The base of the bladder was located 8cm above the pubic bone on percussion. dactyloscopy: prostate hyperplasia of the third degree, urine routine: WBC (++++), RBC (+). Blood creatinine is 155 μmol/L. Based on the patient's condition, the diagnosis and treatment plan should take the form of

A IVU examination for upper urinary tract

B Urine culture examination and treatment of urinary tract infection with sensitive antibiotics

C indwelling urinary catheter to observe the recovery of renal function

D prostate removal surgery after improvement of renal function

E Use of penile clips

F Use of penile condom

47. A 25-year-old woman presented with "left lumbar discomfort for 2 weeks". Renal B-mode ultrasound (4 months ago): left renal malformation tumor. The principles of treatment for renal staggered tumor are.

A Asymptomatic tumors less than 4 cm in diameter are recommended for observation.

B Asymptomatic tumors less than 4 cm in diameter are treated with arterial embolization.

C Tumors larger than 4 cm in diameter with symptoms should be treated with renal unit preservation surgery or selective arterial embolization whenever possible.

D Preservation of renal function should be the primary consideration

E More aggressive treatment may be considered for women of childbearing age

F Pregnancy may reduce the size of the staggered tumor

G Bleeding or ruptured tumor, consider selective arterial embolization to relieve patient risk

H Asymptomatic or minimally symptomatic patients with tumors greater than 4 cm in diameter should be reviewed every 6 months

48. Which of the following are complications of prostatic hyperplasia:

A Bladder stones

B Acute urinary retention

C True diverticulum of the bladder

D Inguinal hernia

E Bladder tumor

49. Extra-renal manifestations of renal cell carcinoma may include all of the following except

A High blood pressure

B rapid blood sedimentation

C erythrocytosis

D hyperkalemia

E Skin rash

50. Boy, 4 years old, whose mother noticed an abnormal shape of the abdomen when she bathed the child, came to the clinic and on examination diagnosed nephroblastoma, which of the following would be reasonable?

A anemia and fever

B Malaise state

C hypertension

D abdominal mass

E gross hematuria

51. Giant hydronephrosis is defined as ( )

A Adult hydronephrosis with a volume of more than 1000 ml

B Adult hydronephrosis of more than 1500 ml

C Adult hydronephrosis of more than 800 ml

D Pediatric hydronephrosis of more than 600 ml

E Pediatric hydronephrosis with a volume greater than the total amount of urine in 24 hours.

52. During rectal palpation, the following structures may be palpated ( )

A uterus

B prostate gland

C seminal vesicles

D ovary

E posterior vaginal wall

53. The following statements about tumors of the urinary tract are correct ( )

A Renal tumors are common in adults

B Nephroblastoma is one of the most common malignant solid tumors in infants and children

C Bladder tumors are the most common tumors of the urinary system

D Prostate cancer is extremely common in Europe and the United States, but is relatively rare in China

E Testicular tumors are the most common solid tumors in young adult men aged 20-40 years old

54. Female, 56 years old, intermittent painless hematuria throughout for more than 2 years. Cystoscopy showed a papilloma of about 3cm x 2.5cm x 2cm with a tip on the right wall of the bladder. Which of the following treatments is correct ( )

A Postoperative immunotherapy

B partial cystectomy

C total cystectomy

D Postoperative intravesical instillation chemotherapy

E Transurethral cystectomy for bladder tumors

55. The types of renal cell carcinoma not included in the 2004 World Health Organization (WHO) pathohistologic classification are ( )

A Mucinous tubular and spindle cell carcinoma

B Cystadenocarcinoma

C Bellini collecting duct carcinoma

D Suspicious cell carcinoma

E Sarcomatoid carcinoma

56. Non-muscle invasive bladder cancer is grouped according to risk of recurrence and prognosis, which of the following characterize the high-risk group ( )

A Multiple or highly recurrent and tumor diameter >3 cm

B Bladder cancer stage T1

C Bladder cancer in situ (Cis)

D Bladder cancer stage G3

E Bladder cancer stage TaG1

57. Which of the following descriptions is not consistent with prostate sarcoma ( )

A Rectal palpation of the prostate is hard as a stone.

B Normal serum prostate-specific antigen (PSA)

C Sensitive to endocrine therapy

D Bone metastases are often osteogenic in nature.

E Prevalent in elderly patients

58. Which of the following are precancerous lesions of penile cancer ( )?

A Intraepithelial neoplasia grade III (Ⅲ)

B Giant condyloma acuminatum

C Proliferative erythema (Queyrat)

D Vulvar carcinoma in situ (Bowen disease)

E Eczema-like carcinoma (Paget disease)

59. Which of the following is not a target area for postoperative radiotherapy in patients with stage I testicular seminomas ( )

A Contralateral testis

B inguinal lymph nodes

C Para-abdominal lymph nodes

D mediastinal lymph nodes

E iliac paravascular lymph nodes

60. The patient is a 32-year-old male who presented with pain and enlargement of the left testis with a heavy feeling 1 month ago. The size of the left testis on palpation was about 4cm×3cm×3cm, and the superficial lymph nodes were not palpable and enlarged. Ultrasonography suggested the possibility of left testicular tumor.

The possibility of a tumor in the left testis was suggested by the ultrasound examination. Which of the following tumor markers must be tested?

A Alpha-fetoprotein (AFP)

B human chorionic gonadotropin (β-hCG)

C lactate dehydrogenase (LDH)

D Neuronal enolase (NSE)

E Placental alkaline phosphatase (PALP)

61. The patient is a 32-year-old male who presented with pain and enlargement of the left testis with a heavy feeling 1 month ago, and the size of the left testis on palpation was about 4cm×3cm×3cm, with no palpable enlargement of the superficial lymph nodes. Ultrasonography suggested the possibility of a left testicular tumor. The patient underwent radical resection of the left testis, and the postoperative pathology report was seminoma. Further examination revealed enlarged retroperitoneal lymph nodes, the largest of which was about 3cm×2cm and partially fused to encapsulate the abdominal aorta, and no abnormality was seen on chest CT examination, and the appropriate postoperative adjuvant treatment for this patient was ( )

A chemotherapy

B radiotherapy

C endocrine therapy

D observation

E immunotherapy

62. Treatment of patients with a clinical diagnosis of stage I spermatogonia includes ( )

A Radical testicular surgery

B Neoadjuvant chemotherapy + radical testicular surgery

C Retroperitoneal lymph node dissection to accurately stage the tumor and guide subsequent treatment

D Adjuvant radiotherapy

E Cisplatin-centered combination chemotherapy

63. ACTH-dependent Cushing's syndrome should be treated with transsphenoidal microadenoma removal as the first choice, or ( ) if surgery fails or is inoperable.

A Subrenal gland tumor removal

B Pituitary radiotherapy

C drug therapy

D antibiotic therapy

E Bilateral subtotal adrenalectomy

64. The correct account of preoperative patient circulatory system adjustment for adrenal surgery is ( ).

A The patient's cardiac functional status should be fully understood before surgery.

B Routinely apply a period of myocardial polarization therapy

C Adjust the blood volume to reach or approach the normal level as much as possible.

D A small amount of potassium-sparing diuretics can be applied to reduce the patient's cardiac load.

E Potassium-sparing diuretics may be applied in large amounts to reduce the patient's cardiac load.

65. Clinical manifestations of nonfunctioning adrenocortical carcinoma include ( ).

A Intermittent high fever

B rapid onset of disease

B rapid onset of disease C malaise and lethargy

D intermittent low fever

E slow onset of disease

66. The scope of regional lymph node dissection for limited renal cancer includes ( ).

A Lymph nodes on the right side from the right diaphragmatic pedicle, along the periphery of the inferior vena cava down to the bifurcation of the abdominal aorta and the right side of the kidney

A Retroperitoneal lymph nodes within the area of lymphatic drainage.

B Left side from the foot of the left diaphragm, along the periphery of the abdominal aorta down to the lymph nodes at the bifurcation of the abdominal aorta and the left side of the kidney

Retroperitoneal lymph nodes within the area of lymphatic drainage.

C Extended lymph node dissection adds to the regional lymph node dissection the lymph nodes between the abdominal aorta and inferior vena cava and the lymph nodes contralateral to the affected kidney.

Lymph nodes between the abdominal aorta and inferior vena cava and anterior and posterior lymph nodes of the abdominal aorta or inferior vena cava on the side opposite to the kidney

D. Lymph nodes on the right side from the right diaphragmatic pedicle, along the periphery of the abdominal aorta down to the bifurcation of the abdominal aorta, and on the left side of the kidney.

Retroperitoneal lymph nodes within the area of lymphatic drainage.

E Lymph nodes on the left side from the left diaphragmatic pedicle, around the inferior vena cava down to the bifurcation of the abdominal aorta and the right kidney

Retroperitoneal lymph nodes within the area of lymphatic drainage

67. Renal vascular smooth muscle lipoma tumors larger than 4 cm may be considered an option ( ).

A Conservative treatment

B renal artery embolization

C Tumor enucleation

D radical nephrectomy

E Nephrectomy

68. The correct description of the pathology of simple renal cysts is ( ).

A most of them are single, can also be multiple

B Most of them occur in the renal parenchyma

C Isolated and spherical, with thin and smooth walls.

D dark blue in appearance, mostly undivided

E Isolated spherical cysts with thick, non-smooth walls.

69. A pararenal cyst is seen on IVU ( ).

A Rounded mass compressing the renal pelvis in the pararenal hilum or renal sinus

B arcuate indentation and distortion

C fluid dark area near the renal hilum

D Easily mistaken for hydronephrosis

E Clear border next to the renal pelvis
