# Supplementary material for: Amplifying Chinese physicians’ emphasis on patients’ psychological states beyond urologic diagnoses with ChatGPT – a multicenter cross-sectional study
Source: Int J Surg. 2024 Jul 2;110(10):6501–8. doi: 10.1097/JS9.0000000000001775 (PMC11487044; doi:10.1097/JS9.0000000000001775)
Supplement: SUPPLEMENTARY MATERIAL [file js9-110-6501-s005.docx]

**Supplementary file 3**

**Amplifying Chinese Physicians' Emphasis on Patients' Psychological States Beyond Urologic Diagnoses with ChatGPT—A Multi-Center Cross-Sectional Study**

**The following are additions to the "Results" section.**

**Results**

**Grouped based on the benignity or malignancy of the disease**The topics were grouped according to benign diseases, and statistical analysis demonstrated that the attending physician group scored significantly higher than both the resident group (p=0.004) and the ChatGPT 3.5 group (p=0.0161). Similarly, the ChatGPT 4.0 group scored higher than the ChatGPT 3.5 group (p=0.004) and the resident group (p=0.033) (Figure 2C). The topics were grouped according to malignant disease, and statistical analysis exhibited that the ChatGPT 4.0 group scored significantly higher than the ChatGPT 3.5 group (p=0.0022) (Figure 2C).

**Grouping based on urological knowledge section**

Statistical analysis revealed no significant differences between the Resident, Attending, ChatGPT 3.5, and ChatGPT 4.0 groups in research on urinary tuberculosis, hydronephrosis, anatomy, prostate cancer, kidney transplantation, and general oncological issues (Supplementary Figure 2).

**Comparison of Score Rates by Group**

Statistics on the question score rates for each comparison group exhibited that GPT-4 group and Attending physician group performed similarly. Collectively, the GPT-4 group performed better with higher scoring rates at both the 2-point and 5-point scales (Figure 3A). Statistics on the correct rate (CR) and error rate (ER) for each comparison group demonstrated that the AI group performed significantly better than the physician group. Statistical analysis demonstrated that the ChatGPT-4 group had a higher CR than the Resident (p=0.002), Attending (p=0.011), and ChatGPT-3.5 (p＜0.0001) groups, with the difference between the groups being statistically significant (Figure 3B).

**Assessment of performance on case studies and multiple-choice questions**

The case study questions comprised of 9 urology simulation cases and were subdivided into 46 subtopics. Statistical analysis showed that the GPT-4.0 group scored higher than ChatGPT 3.5 group (p=0.0010), while the Attending group scored higher than the ChatGPT 3.5 (p=0.0184) and Resident group (p=0.0201). (Figure 3C).

Additionally, compile the performance data for each group on the independent indeterminate multiple-choice questions. Statistical analysis exhibited that ChatGPT 4.0 had higher scores compared to ChatGPT 3.5 (p=0.0273), and the Attending physician group also had higher scores compared to ChatGPT 3.5 (p=0.0442). The differences between the other comparison groups were not statistically significant (Figure 3D).
