# Supplementary material for: Amplifying Chinese physicians’ emphasis on patients’ psychological states beyond urologic diagnoses with ChatGPT – a multicenter cross-sectional study
Source: Int J Surg. 2024 Jul 2;110(10):6501–8. doi: 10.1097/JS9.0000000000001775 (PMC11487044; doi:10.1097/JS9.0000000000001775)
Supplement: SUPPLEMENTARY MATERIAL [file js9-110-6501-s006.docx]

**Supplementary Table 1**

**Amplifying Chinese Physicians' Emphasis on Patients' Psychological States Beyond Urologic Diagnoses with ChatGPT—A Multi-Center Cross-Sectional Study**

**Supplementary Table 1. Psychological Support Capacity Assessment Scale**

| **1.1 Quality of psychological counseling for patients with urinary incontinence scale** |
| --- |
| **1. Objective diagnosis and presentation (total points: 30)** |
| **Clearly explains the types and causes of urinary incontinence (10 points)** |
| **Provided a comprehensive overview of the current stage of the patient's condition (10 points)** |
| **Explained the patient's treatment options (10 points)** |
| **2. Counseling skills (total: 35 points)** |
| **Appropriate models of psychological detachment are used, such as cognitive behavioral therapy (15 points)** |
| **The content of the diversion is specific and individualized (10 points)** |
| **Adequate attention was given to the patient's feelings and concerns during the de-escalation (10 points)** |
| **3. Communication with patients (total points: 20)** |
| **Ability to communicate with patients in clear, concise language (10 points)** |
| **Comprehensive and accurate answers to patient questions (10 points)** |
| **4. Resources and references (total: 15 points)** |
| **Relevant mental health resources are provided (7.5 points)** |
| **Further reading and information was provided to help patients better understand their condition (7.5 points)** |

| **1.2 Quality of Psychological Diversion Scale for ED Patients** |
| --- |
| **1. Specialized knowledge (Total 20 points)** |
| **In-depth understanding of ED (10 points)** |
| **Recommendations based on the latest medical research (10 points)** |
| **2. Communication skills (15 points)** |
| **Easy-to-understand explanatory approach (7 points)** |
| **Listening patiently and responding to patient concerns (8 points)** |
| **3. Individualized recommendations (20 points)** |
| **Customized recommendations for patient-specific situations (10 points)** |
| **Consideration of patient's lifestyle, psychological state (10 points)** |
| **4. Supportive and encouraging (15 points)** |
| **Provide emotional support and encouragement (8 points)** |
| **Helping patients build confidence to cope with ED (7 points)** |
| **5. Privacy and sensitivity (15 points)** |
| **Protection of patient privacy (8 points)** |
| **Sensitivity to patient emotions and concerns (7 points)** |
| **6. Follow-up and provision of resources (15 points)** |
| **Follow-up counseling or treatment opportunities (7 points)** |
| **Recommended additional resources or readings (8 points)** |

| **1.3 Quality of psychological counseling for patients with preoperative anxiety scale** |
| --- |
| **1. Understanding and empathy (Total: 25 points)** |
| **Does the physician demonstrate an in-depth understanding of the patient's situation? (7 points)** |
| **Does the physician show enough compassion and concern? (6 points)** |
| **Are patients encouraged to discuss their questions and concerns openly? (6 points)** |
| **Is the patient provided with a safe, stress-free environment to facilitate communication? (6 points)** |
| **2. Education and knowledge transfer (Total: 25 points)** |
| **Did the physician provide enough medical knowledge to help the patient understand their symptoms? (7 points)** |
| **Are the pros and cons of different treatment options explained? (6 marks)** |
| **Does it answer the patient's concerns about treatment and side effects? (6 points)** |
| **Are patients educated about how to communicate with their partner about their problems? (6 points)** |
| **3. Professionalism and relevance of the methodology (total: 25 points)** |
| **Is the advice given based on scientific evidence and current best practice? (13 marks)** |
| **Are the recommendations made customized to the individual patient? (12 points)** |
| **4. Psychotherapeutic techniques (Total: 20 points)** |
| **Did the practitioner apply appropriate psychotherapeutic techniques to help the patient deal with anxiety, fear, or other emotional problems? (10 points)** |
| **Are specific psychotherapeutic methods or techniques provided, such as relaxation techniques, cognitive behavioral therapy, etc.? (10 points)** |
| **5. Feedback (Total: 5 points)** |
| **Does the practitioner encourage patients to provide feedback during or after treatment? (5 points)** |
