# Supplementary material for: Amplifying Chinese physicians’ emphasis on patients’ psychological states beyond urologic diagnoses with ChatGPT – a multicenter cross-sectional study
Source: Int J Surg. 2024 Jul 2;110(10):6501–8. doi: 10.1097/JS9.0000000000001775 (PMC11487044; doi:10.1097/JS9.0000000000001775)
Supplement: SUPPLEMENTARY MATERIAL [file js9-110-6501-s008.pdf]

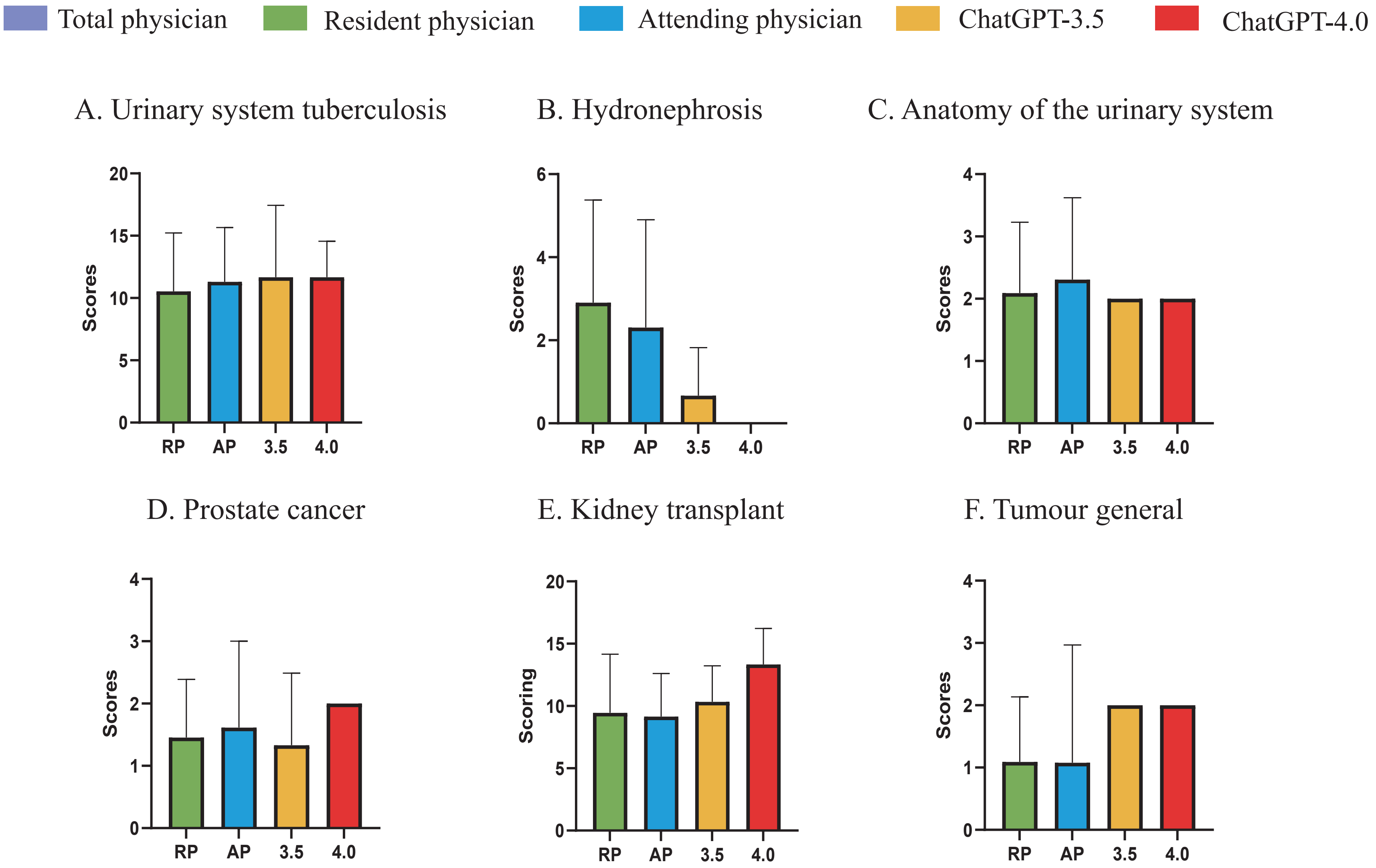

Supplementary Figure 2. Assessment of Artificial Intelligence Model Performance vs. Clinician Comparison Group (A. Urinary system tuberculosis, B. Hydronephrosis, C. Anatomy of the urinary system, D. Prostate cancer, E: Kidney transplant, F: Tumor general).
