# Supplementary material for: Amplifying Chinese physicians’ emphasis on patients’ psychological states beyond urologic diagnoses with ChatGPT – a multicenter cross-sectional study
Source: Int J Surg. 2024 Jul 2;110(10):6501–8. doi: 10.1097/JS9.0000000000001775 (PMC11487044; doi:10.1097/JS9.0000000000001775)
Supplement: SUPPLEMENTARY MATERIAL [file js9-110-6501-s009.docx]

**Supplementary Table 2**

**Amplifying Chinese Physicians' Emphasis on Patients' Psychological States Beyond Urologic Diagnoses with ChatGPT—A Multi-Center Cross-Sectional Study**

**Supplementary Table 2.1 Results of the assessment of clinical psychological guidance issues**

**(Urinary incontinence)**

| **Question Number** | **Reviewer 1（3.5）** | **Reviewer 1（4.0）** | **Reviewer 2（3.5）** | **Reviewer 2（4.0）** |
| --- | --- | --- | --- | --- |
| **1** | **97** | **95** | **93** | **93** |
| **2** | **86** | **88** | **89** | **90** |
| **3** | **88** | **89** | **85** | **87** |
| **4** | **85** | **89** | **85** | **91** |
| **5** | **85** | **92** | **85** | **89** |
| **6** | **91** | **98** | **89** | **96** |
| **7** | **85** | **86** | **85** | **85** |
| **8** | **87** | **92** | **87** | **92** |
| **9** | **88** | **92** | **89** | **89** |
| **10** | **87** | **93** | **87** | **93** |

**Supplementary Table 2.2 Results of the assessment of clinical psychological guidance issues
 (Erectile dysfunction)**

| **Question Number** | **Reviewer 1（3.5）** | **Reviewer 1（4.0）** | **Reviewer 2（3.5）** | **Reviewer 2（4.0）** |
| --- | --- | --- | --- | --- |
| **1** | **91** | **96** | **91** | **98** |
| **2** | **85** | **89** | **85** | **90** |
| **3** | **90** | **96** | **93** | **95** |
| **4** | **90** | **97** | **93** | **97** |
| **5** | **88** | **95** | **88** | **94** |
| **6** | **94** | **96** | **92** | **95** |
| **7** | **90** | **95** | **90** | **93** |
| **8** | **86** | **88** | **88** | **91** |
| **9** | **89** | **87** | **86** | **89** |
| **10** | **92** | **90** | **90** | **90** |

**Supplementary Table 2.3 Results of the assessment of clinical psychological guidance issues (Preoperative anxiety)**

| **Question Number** | **Reviewer 1（3.5）** | **Reviewer 1（4.0）** | **Reviewer 2（3.5）** | **Reviewer 2（4.0）** |
| --- | --- | --- | --- | --- |
| **1** | **87** | **94** | **89** | **98** |
| **2** | **83** | **90** | **85** | **92** |
| **3** | **89** | **97** | **87** | **94** |
| **4** | **92** | **99** | **90** | **97** |
| **5** | **85** | **96** | **89** | **96** |
| **6** | **95** | **88** | **90** | **97** |
| **7** | **86** | **93** | **89** | **98** |
| **8** | **84** | **91** | **83** | **90** |
| **9** | **83** | **90** | **83** | **90** |
| **10** | **91** | **84** | **92** | **85** |
