# Supplementary material for: Amplifying Chinese physicians’ emphasis on patients’ psychological states beyond urologic diagnoses with ChatGPT – a multicenter cross-sectional study
Source: Int J Surg. 2024 Jul 2;110(10):6501–8. doi: 10.1097/JS9.0000000000001775 (PMC11487044; doi:10.1097/JS9.0000000000001775)
Supplement: SUPPLEMENTARY MATERIAL [file js9-110-6501-s010.docx]

**Supplementary file 4**

**Amplifying Chinese Physicians' Emphasis on Patients' Psychological States Beyond Urologic Diagnoses with ChatGPT—A Multi-Center Cross-Sectional Study**

**The following are responses of different ChatGPT models in 30 clinical psychological support scenarios.**

**Psychosocial support capacity results for ChatGPT 4.0**

1. Q：Patients with urinary incontinence are often depressed because of the shame they feel. How should the physician go about adjusting the patient's state of mind?

A：1. **Adequately understand and assess the patient's emotional response**: Understand the patient's concerns, fears and feelings of embarrassment through dialog with the patient. Ensure that the patient feels understood and cared for. 2. **Provide information and education**: explain the causes of incontinence, treatment options and preventive measures. Understanding the causes and possible treatment options may reduce the patient's feelings of anxiety and embarrassment. 3. **Encourage active participation in treatment**: Let patients know that they can improve their symptoms by actively participating in treatment, e.g., through physical therapy, diet and lifestyle changes. 4. **Establish a support system**: If possible, encourage patients to join relevant support groups and share their experiences with others who also suffer from incontinence. 5. **Appropriate psychological counseling**: If the emotional problems are severe, professional assistance from a psychiatrist or psychotherapist may be required. Some psychotherapies, such as Cognitive Behavioral Therapy (CBT), may be helpful in dealing with the sufferer's depression and anxiety. 6. **Promote positive attitude towards life and self-acceptance**: Emphasize that incontinence is not a personal fault or a stigma. Through positive encouragement and support, patients are helped to accept their situation and face it positively. 7. **Closely follow up on treatment results**: Keep abreast of the progress of treatment and adjust the treatment program when necessary so that patients feel valued and cared for.

2. Q：How should physicians explain to patients when they refuse incontinence treatment because they are concerned about possible side effects?

A：1. **Ask for concerns in detail**: First, it is important to understand in detail what specific side effects the patient is concerned about and what information or understanding these concerns are based on. This helps the physician to target explanations and clarifications.2. **Provide accurate information**: explain all aspects of the treatment program, including expected benefits, possible risks, and side effects. Language that is easily understood by the patient should be used, avoiding overly complex medical terminology.3. **Explain the likelihood and management of side effects**: detail the likelihood of side effects and explain how the healthcare team will manage and mitigate them. Emphasize that not everyone will experience these side effects and that many can be effectively managed with medications or other measures.4. **Encourage patients to ask questions**: Encourage patients to ask questions and answer them patiently. Patient questions may reveal deep-seated concerns and misconceptions.5. **Provide written information**: If possible, provide written information that the patient can read and digest slowly at home. The information should include a detailed description of the treatment, as well as contact information in case the patient has any further questions or concerns.6. **Emphasis on Individualized Treatment**: Explain why this particular treatment plan is recommended and emphasize that the treatment plan is tailored to the patient's specific situation. Each person's situation is unique and therefore side effects may vary.7. **Keep open communication**: Emphasize that patients can communicate with their healthcare team at any time to share their feelings and concerns. Good communication helps build trust, which is essential to receiving treatment.8. **Provide time and support**: Understand that the patient may need time to think about it, provide support, and offer the assistance of a counselor if needed.

3. Q：How should physicians or psychologists help patients when they are afraid of establishing intimate relationships with people due to the psychological trauma caused by incontinence?

A：1. **In-depth understanding of the patient's feelings**: Engage in an in-depth dialog to understand the patient's specific concerns and feelings. Find out how the patient views his or her body and the impact of incontinence on his or her life.2. **Provide education and insight**: Explain that incontinence is a medical problem, not a personal failure or weakness. Emphasize that many people are affected by this problem and that there are many effective treatments available to help improve symptoms. 3. **Perform psychotherapy**: Individual or group psychotherapy, such as cognitive-behavioral therapy, can help patients identify and change negative beliefs about self-image and relationships. 4. **Teach communication skills**: Help patients learn how to communicate about their condition with a partner or close friend. This may include role-playing and feedback so that patients can practice in a comfortable and supportive environment. 5. **Promote self-acceptance and self-compassion**: help patients accept their situation and understand that they are not incomplete or unworthy of love because of this problem through a careful, empathetic and supportive approach. 6. **Provide social support**: refer or direct the patient to support groups or social events to interact with and learn from others with similar experiences. 7. **Ongoing Medical Treatment and Follow-Up**: work closely with the patient to find the most effective medical treatment plan, with ongoing evaluation and adjustment. 8. **Family or Partner Involvement**: if appropriate, consider counseling with the patient's family or partner so they are informed and learn how to provide support. 9. **Cultivate a positive outlook on life**: Encourage the patient to explore new hobbies and social activities, which can help increase self-confidence and reduce feelings of isolation associated with incontinence.

4. Q：How can physicians encourage and support patients when they refuse to go out in public for fear of becoming incontinent?

A：When patients refuse to go out and about for fear of becoming incontinent in public, this can have a serious impact on their social life and overall quality of life. Physicians can encourage and support patients in the following ways:

1. **Understanding the patient's concerns**: engaging in open and honest communication with the patient to understand their specific concerns and emotions in order to target support and solutions. 2. **Provide medical solutions**: review and potentially adjust the patient's medical treatment plan to ensure that incontinence symptoms are minimized. This may include medication, physical therapy, or other specialized interventions.

3. **Teach management skills**: Teach patients daily skills and strategies such as voiding diaries, timed voiding, and wearing absorbent undergarments to increase their confidence when out and about.

4. **Gradual Exposure**: Work with the patient to set up gradually increasing goals for going outside, starting with short close up outings and gradually increasing the difficulty and distance. Each step can be evaluated and progress celebrated in collaboration with a physician or therapist.

5. **Provide psychological support**: encourage the patient to express feelings and provide emotional support and encouragement. Psychotherapy, such as cognitive-behavioral therapy, may be helpful in dealing with the anxiety and fear associated with incontinence.

6. **Encourage social interaction**: Encourage the patient to interact with family and friends and find a supportive and understanding social environment. Support groups and social activities may also help increase the patient's confidence and comfort.

7. **Provide practical information**: for example, provide information about the location of nearby public restrooms and tips on how to maintain personal hygiene while out and about.

8. **Set personalized goals and rewards**: Work with the patient to set realistic goals for going out and provide positive feedback and rewards to celebrate each success.

9. **FAMILY AND COMMUNITY SUPPORT**: teaches family members, relatives and friends how to provide support and encourages them to participate in the patient's treatment and recovery process.

10. **Long-term follow-up and assessment**: Continuously monitor the patient's progress, conduct regular assessments, and adjust treatment and support plans as needed.

5.Q：How should physicians build trust with patients when they fear that discussing incontinence will make them look weak and thus avoid communicating with their physicians?

Incontinence can be a very personal and sensitive matter, and for many people, discussing it can be embarrassing or uncomfortable. When patients avoid communicating with their physicians because they fear that discussing incontinence will make them look weak, here are some steps physicians can take to build their patients' trust:

A：1. Protect privacy: Ensure that the environment in which you communicate with your patients is private and comfortable. Make it clear that all information will be treated in the strictest confidence and used only for medical purposes.

2. Demonstrate empathy and understanding: show patients that you understand their concerns and care about their feelings by using open, non-judgmental language and attitudes.

3. Communicate incrementally: don't delve into sensitive details right away. Establish a basic level of trust and comfort first, then gradually guide the conversation to incontinence issues.

4. Provide education and information: By educating patients about the medical aspects of incontinence, you can help alleviate their feelings of embarrassment or shame. Explain that this is a common medical problem that affects many people and that there are multiple treatment options that can help.

5. Ask appropriate questions**: encourage patients to share their feelings and concerns by asking open-ended, non-threatening questions. Give them plenty of time to answer and express your interest and understanding by listening.

6. Planning with patients: Give patients an active role in making decisions about their treatment plan. By asking for their thoughts and opinions and working with them to determine next steps, you can increase their sense of involvement and trust.

7. Provide ongoing support: Clearly express your willingness as their physician to provide ongoing support and assistance. Follow up regularly to show that you care about their progress and well-being.

8. Advocates Dignity: emphasizes that every person has the right to the best medical care and promises that their dignity and self-respect will be respected and protected.

9. Encourage family support: If the patient agrees, family members can be encouraged to participate in discussions and treatment planning to increase support and understanding.

10.Provide professional psychological support: If needed, a psychologist can be recommended to work with the patient to further address the emotional issues associated with incontinence.

In summary, by demonstrating empathy, understanding, patience, and expertise, practitioners can build trust with their patients and make them feel comfortable discussing incontinence. This trust building may take time and effort, but it is a key part of promoting patient health and well-being.

6. Q：When patients isolate themselves at home because they are afraid that others will know about their incontinence problem, how should physicians or psychologists counsel them?

Urinary incontinence issues can be a very personal and sensitive matter, and for many people, discussing it can be embarrassing or uncomfortable. When patients are concerned that discussing incontinence issues will make them look weak and thus avoid communicating with their physician, here are some steps physicians can take to build patient trust:

1. **Protect privacy**: Ensure that the environment in which you communicate with your patients is private and comfortable. Make it clear that all information will be treated in the strictest confidence and used only for medical purposes.

2. **Demonstrate empathy and understanding**: show patients that you understand their concerns and care about their feelings by using open, non-judgmental language and attitudes.

3. **Communicate incrementally**: don't delve into sensitive details right away. Establish a basic level of trust and comfort first, then gradually guide the conversation to incontinence issues.

4. **Provide education and information**: By educating patients about the medical aspects of incontinence, you can help alleviate their feelings of embarrassment or shame. Explain that this is a common medical problem that affects many people and that there are multiple treatment options that can help.

5. **Ask appropriate questions**: encourage patients to share their feelings and concerns by asking open-ended, non-threatening questions. Give them plenty of time to answer and express your interest and understanding by listening.

6. **Planning with patients**: Give patients an active role in making decisions about their treatment plan. By asking for their thoughts and opinions and working with them to determine next steps, you can increase their sense of involvement and trust.

7. **Provide ongoing support**: Clearly express your willingness as their physician to provide ongoing support and assistance. Follow up regularly to show that you care about their progress and well-being.

8. **Advocates Dignity**: emphasizes that every person has the right to the best medical care and promises that their dignity and self-respect will be respected and protected.

9. **Encourage family support**: If the patient agrees, family members can be encouraged to participate in discussions and treatment planning to increase support and understanding.

10. **Provide professional psychological support**: If needed, a psychologist can be recommended to work with the patient to further address the emotional issues associated with incontinence.

In summary, by demonstrating empathy, understanding, patience, and expertise, practitioners can build trust with their patients and make them feel comfortable discussing incontinence. This trust building may take time and effort, but it is a key part of promoting patient health and well-being.

7. How should physicians or psychologists counsel patients when they isolate themselves at home because they are afraid that others will know about their incontinence problem?

A: When patients isolate themselves at home because they are afraid that others will know about their incontinence problem, this is a complex emotional issue that needs to be handled with care. A physician or psychologist can use the following strategies to counsel and support them:

1. **Build a trusting relationship**: First, establish a safe, nonjudgmental communication environment with the patient. Let them know that their feelings are normal and that you are there to support, not judge them.

2. **Understand the patient's concerns**: gain a deeper understanding of the patient's concerns and fears in order to provide individualized support and solutions. Try to understand their specific reasons for isolating themselves.

3. **Educate and enlighten**: explain that incontinence is a common medical condition that affects many people. Provide information about treatment and management strategies to alleviate their concerns and discomfort.

4. **Provide support and resources**: inform patients about support groups and other resources, such as local incontinence associations or online communities, so that they can connect with others who are experiencing the same thing.

5. **Encourage social engagement**: Progressively encourage patients to interact with family, friends, and the community. Where possible, work with their family members or friends to provide support together.

6. **SET REALISTIC GOALS**: Work with the patient to set small but realistic goals to gradually increase their social engagement. Celebrate each small accomplishment and provide ongoing encouragement.

7. **Provide psychotherapy**: Consider recommending psychotherapy, especially if isolated problems are more complex or deep-rooted. Approaches such as cognitive-behavioral therapy may be helpful in dealing with social fears and isolation related to incontinence.

8. **Provide family guidance**: If applicable, teach family members how to provide support and understanding and work with them to promote a more supportive family environment.

Providing family guidance is an important aspect of dealing with the problem of isolating oneself for a person with urinary incontinence, especially when the family is the person's primary social network. Here are some specific steps on how to teach family members to provide support and understanding and work with them to promote a more supportive family environment:

1. **Understanding Family Dynamics**: work with the patient and family members to assess the family's needs and dynamics and determine the type of support they need.

2. **Education and information sharing**: provide family members with basic information about incontinence, explaining what it is, why it occurs, and how to manage it. This can help reduce misunderstandings and anxiety.

3. **Provide communication skills training**: Teach family members how to communicate with the patient in an open, supportive, and non-judgmental manner. Emphasize the importance of listening and empathy.

4. **SET CLEAR EXPECTATIONS AND BOUNDARIES**: Work with family members to set clear goals and expectations so they can provide appropriate support without feeling pressured.

5. **Encourage cooperation and participation**: Encourage family members to participate in the patient's plan of care, such as attending doctor's appointments or participating in setting treatment goals.

6. **Provide emotional support strategies**: teach family members how to provide emotional support while also taking care of their own emotional needs.

7. **Emphasize privacy and dignity**: explain how to respect the patient's privacy and dignity when providing support, including guidance on when and how to discuss incontinence.

8. **Establish regular check-ins**: arrange regular meetings or phone calls with family members to discuss progress, resolve problems, and provide ongoing support.

9. **Provide external resources and support**: If needed, direct family members to external support, such as joining a continence support group or seeking out a specialized mental health professional.

10. **Emphasize teamwork**: Emphasize the spirit of teamwork among the physician, patient, and family members, and encourage all to work together to improve the patient's quality of life.

Through these strategies, physicians can help family members more effectively support patients with incontinence, thereby reducing their feelings of isolation and fear and promoting a more supportive and understanding family environment. This multifaceted approach contributes to an overall improvement in the patient's mental and emotional health and promotes their social engagement and well-being.

9. **Monitoring Progress**: Meet with patients regularly to assess their progress and mental health. Make timely adjustments to the treatment plan if the situation does not improve or worsens.

10. **Remind patients of their right to confidentiality**: Emphasize that all discussions and information about their condition will be held in the strictest confidence to alleviate concerns about sharing information with outsiders.

Through these strategies, physicians and psychologists can help patients overcome the fear of isolating themselves due to incontinence issues and encourage them to reconnect with their social environment and community. Ongoing support and individualized treatment may be the key to success.

8. Q：How can physicians help patients when they have sleep disorders due to stress caused by incontinence problems?

A：When patients suffer from stress due to incontinence problems that cause sleep disturbances, physicians can help in the following ways:

1. **Understanding the depth of the problem**: First, through detailed communication with the patient, understand the specific impact of incontinence on their daily life and sleep. Analyze whether stress, fear, depression, or other emotional issues are causing the sleep disorder.

2. **Provide education and explanation**: explain to the patient the nature and commonality of incontinence and emphasize its manageable and treatable aspects. Appropriate education can reduce fear and stress.

3. **Establish an appropriate plan of care**: develop an individualized incontinence management plan based on the patient's needs and circumstances, including possible medication, pelvic floor muscle training, etc.

4. **Introduce sleep hygiene measures**: Teach patients good sleep hygiene practices, such as keeping a consistent schedule, avoiding caffeine and alcohol, and performing only activities such as sleeping in bed.

5. **Emotional support and psychological interventions**: provide emotional support and, if necessary, introduce psychological treatments such as cognitive behavioral therapy to deal with anxiety and stress related to incontinence.

6. **Rational Use of Medications**: if the sleep disorder is severe, it may be necessary to consider short-term medication interventions. However, this should be used as a last resort and carefully monitored.

7. **Teaching Relaxation Techniques**: Teach the patient to use relaxation techniques such as deep breathing and progressive muscle relaxation to aid in falling asleep.

8. **Follow-up and Monitoring**: Follow up on the patient's progress regularly and adjust the treatment plan as needed. Closely monitor the patient's sleep status and overall health.

9. **Involve family support**: if applicable, work with family members to ensure they understand the patient's situation and know how to provide support.

10. **Provide professional referrals**: if the above measures are not effective, consider referring the patient to a sleep disorder or mental health professional.

By using a combination of the above methods, physicians can help patients with incontinence address the resulting sleep disorders. The key is to take a comprehensive, individualized approach and work closely with the patient to address their specific needs and concerns.

9. Q：How should physicians recommend alternative treatments to patients when they have persistent depression due to chronic incontinence problems but refuse medication for fear of its side effects?

A：When patients have persistent depression due to chronic incontinence problems and refuse medication because they are concerned about the side effects of medication, physicians may consider the following steps to recommend alternative treatments to them:

1. **In-depth understanding of the patient's concerns**: communicate with the patient in depth to understand the specific reasons why they are concerned about the side effects of medication to provide tailored support and explanations.

2. **Explain the severity of the depressive illness**: emphasize the severity of the depressed mood and the need for treatment to ensure that the patient understands the impact that not treating may have on their overall health and quality of life.

3. **Recommend non-pharmacologic treatment options**:

- **Cognitive Behavioral Therapy (CBT)**: CBT is an evidence-supported psychotherapeutic approach to the treatment of depressive disorders. Explain to patients how CBT works and how it can improve feelings by changing patterns of thinking and behavior.

- **Counseling and support groups**: refer patients to specialized counseling and support groups to share experiences and get support from others.

- **Lifestyle interventions**: Teach patients about lifestyle changes such as healthy eating, regular exercise and good sleep habits to improve emotional well-being.

- **Meditation and Relaxation Techniques**: guide patients in relaxation techniques such as meditation and progressive muscle relaxation that can help reduce stress and depression.

4. **Provide a comprehensive treatment plan**: work with patients to develop a comprehensive treatment plan that includes the non-pharmacological treatments mentioned above, ensuring that they understand and agree with the proposed program.

5. **Closely follow up and evaluate**: meet with the patient regularly to follow up on progress, evaluate the effectiveness of the non-pharmacological treatments, and adjust the treatment plan accordingly.

6. **Involves family and social support**: encourages support from the patient's family members and friends and teaches them how to help the patient.

7. **Provides specialist referrals**: If needed, patients can be referred to a mental health specialist or psychologist for further evaluation and treatment.

By providing non-pharmacological treatment options and thoroughly explaining the various treatments available, physicians can increase patients' willingness to cooperate with treatment and help alleviate their depressed feelings. The key is to ensure that the patient is involved in the decision-making process, understands every aspect of the treatment plan, and feels supported and understood.

10. Q：When patients choose to remain silent for fear of being ridiculed or criticized by family or friends for speaking out about their incontinence, how can physicians reassure and encourage them?

A：When patients choose to remain silent because they are worried that they will be ridiculed or criticized by their family or friends for speaking out about their incontinence problem, physicians can take the following measures to reassure and encourage them:

1. **Ensure privacy and confidentiality**: Emphasize that communication between the physician and patient is private and that information will be kept strictly confidential so that patients feel safe and comfortable sharing their concerns.

2. **Demonstrate empathy and understanding**: show patients that you understand their concerns and affirm that their feelings are valid by listening and being empathetic.

3. **Educate and explain**: explain that incontinence is a common medical condition and not a personal mistake or defect. Many people may experience similar problems and there are many effective treatments available.

4. **Provide support strategies**:

- **Social skills training**: teaches patients how to talk to others about their health condition and how to cope with possible negative reactions.

- **Suggest joining a support group**: recommend that the patient participate in an incontinence support group to talk to people who are going through similar experiences.

5. **Encourage the patient to communicate with family**: if applicable, encourage the patient to share their situation with a trusted family member or friend and provide guidance on how to have these conversations.

6. **Provide family education**: if the patient agrees, provide education with family members to help them understand incontinence and teach them how to provide support.

7. **BUILD TRUSTING RELATIONSHIPS**: Through a series of meetings, build a trusting relationship with the patient so they know they can trust you and rely on you for support.

8. **Follow-up and monitoring**: follow up with patients regularly to see how they are feeling and how they are progressing and make adjustments as needed.

9. **Involve a mental health professional**: If a patient's concerns become difficult to manage, consider referring them to a mental health professional for further support and treatment.

Through the methods described above, physicians can work with patients to manage the social and emotional challenges of incontinence. The key is to show empathy, provide education, and partner with patients to find solutions that work for them. Through a patient-centered approach, patients can be empowered to feel more confident and in control and encouraged to take a more active role in managing their health.

11. Q：How can physicians reassure and convince male patients when they refuse to mention their erectile dysfunction symptoms because of embarrassment and shame?

A：When male patients are embarrassed and ashamed of erectile dysfunction (ED) and refuse to mention their symptoms, physicians can take the following steps to reassure and convince them:

1. **Create a safe and private environment**: ensure that the conversation takes place in a non-intrusive, confidential environment where the patient feels comfortable and safe.

2. **Demonstrate a professional attitude and empathy**: adopt a non-judgmental and empathetic attitude so that patients feel their feelings are understood and respected.

3. **Educate and normalize**: explain to patients that erectile dysfunction is a common medical problem and one that many men may experience. Emphasize that it is not their fault or defect.

4. **Use non-directive questioning**: use a wider range of questions to assess men's health and can ask about sexual health indirectly to reduce patient stress.

5. **Explain the diagnostic and treatment process in detail**: explain the possible causes of erectile dysfunction, as well as the diagnostic and treatment options available, emphasizing the importance of early intervention.

6. **Provide literature and resources**: provide reliable literature and resources for patients to read and learn about ED in private.

7. **Emphasize collaboration and support**: make sure patients know that you, as the physician, will work with them, support them throughout the process, and protect their privacy.

8. **Involve patients in the decision-making process**: encourage patients to participate in the decision-making process for diagnosis and treatment, and consider their wishes and comfort level throughout the process.

9. **Provide psychological support**: if necessary, involve a mental health professional to help patients deal with the emotional issues associated with erectile dysfunction.

10. **Continuous follow-up and monitoring**: continue to support the patient through regular follow-ups, and encourage and support them during the course of treatment.

By doing so, the practitioner can gradually reduce the patient's feelings of embarrassment and shame and encourage them to discuss erectile dysfunction openly. It is important that the entire process be filled with understanding and empathy so that patients feel respected and supported and are thus more likely to cooperate with their physicians in effective diagnosis and treatment.

12. Q：When male patients avoid having sex with their partners because of anxiety about sexual performance, how can physicians encourage them to confront this issue?

A：When male patients avoid having sex with their partners because of anxiety about sexual performance, physicians can take the following steps to encourage them to confront the problem:

1. **Understanding and assessing the problem**: Have an in-depth conversation with the patient to understand their feelings and the specific causes of their anxiety. A thorough understanding of the problem is key to providing effective support and solutions.

2. **Provide emotional support and empathy**: show understanding and concern to ensure the patient feels respected and supported, not judged.

3. **Normalize the problem**: Explaining sexual performance anxiety is a common problem that many people may face and can be addressed like any other health issue.

4. **Educate the Patient**: Provide education about sexual health, sexual functioning, and human physiology; sometimes knowing the scientific facts can dispel fears and misconceptions.

5. **Encourage partner communication**: If possible, encourage patients to participate in treatment or counseling with their partners to increase transparency and trust.

6. **Provide treatment options**: Discuss and provide appropriate treatment options, such as psychotherapy, medication, or lifestyle modifications, depending on the patient's specific situation.

7. **Teach stress management techniques**: patients can be taught how to manage anxiety using deep breathing, meditation, or other relaxation techniques.

8. **Set achievable goals**: Work with patients to set gradual, achievable goals that slowly improve sexual function and increase confidence.

9. **Provide professional referrals**: If necessary, refer the patient to a sex therapist or mental health professional for specialized treatment.

10. **Continuous Follow-up and Encouragement**: Support the patient's progress in the treatment process through regular check-ups and follow-ups, praising and encouraging positive changes.

11. **Encourage a holistic view of health**: Emphasize that sexuality is only one part of overall health and well-being and encourage patients to find satisfaction and happiness in other areas.

Through these strategies, practitioners can help patients understand and confront their sexual performance anxiety, facilitate communication, provide practical tools and support, and guide them toward a more holistic, healthy sex life. This process can take time and patience, as well as close collaboration with mental health and sexual health professionals.

13. Q：When male patients are afraid of the possible side effects of sex therapy or medication, and thus refuse the relevant treatment, what should physicians do to persuade them?

A：When a male patient refuses treatment because he is worried about the possible side effects of sex therapy or medication, the physician can take the following measures to persuade them:

1. **In-depth understanding of the concern**: First, the physician needs to understand what specific side effects the patient is concerned about and ask them where they learned this information. Understanding the patient's specific concerns will help to answer their questions more precisely.

2. **Provide accurate information**: Explain the rationale for the treatment, its effectiveness, and possible side effects in clear, easy-to-understand language. Provide scientific evidence and data to correct possible misunderstandings or misinformation.

3. **Explain risks and benefits**: clearly explain the potential benefits and risks of the treatment so that the patient understands why the treatment is recommended and explains how possible side effects are monitored and managed.

4. **Introduce alternatives**: if applicable, introduce the patient to other possible treatment options, including non-pharmacologic treatments or different types of medications.

5. **Emphasize individualized treatment**: explain to the patient that the treatment plan will be adapted to their specific situation and needs and will be closely monitored to minimize the risk of side effects.

6. **Invite patients to participate in decision-making**: make patients feel that they are part of the treatment decisions, ask for their views and preferences, and encourage them to ask questions.

7. **Provide written materials and resources**: provide written educational materials, such as brochures or reliable online resources, that patients can further understand and consider even at home.

8. **Encourage Second Opinions**: If patients still feel uncertain, they can be encouraged to seek a second opinion, which can help increase trust and comfort.

9. **Regular follow-up and support**: maintain regular contact with patients, provide ongoing support and education, and let them know that the physician is always available for consultation.

Through these strategies, physicians can increase patients' understanding and trust, reduce their fear of side effects, and help them make informed medical decisions. This process can take patience and time, and through open and transparent communication, patient acceptance and satisfaction with treatment can be increased.

14. Q：When male patients are frustrated with their bodies because of erectile dysfunction or premature ejaculation and therefore refuse to undergo the necessary physical examination, how should physicians pacify and persuade them?

A：When male patients are frustrated with their bodies because of erectile dysfunction or premature ejaculation problems, and therefore refuse to undergo the necessary physical examination, physicians should take a careful approach to reassure and convince them. The following are possible steps:

1. **Establish trust and channels of communication**: demonstrate respect for the patient's feelings and privacy through empathetic, understanding language and attitudes to ensure they feel comfortable and safe.

2. **Gain insight into concerns**: ask patients about their specific concerns and feelings in order to better understand their situation and provide appropriate support.

3. **EXPLAIN THE IMPORTANCE OF THE EXAMINATION**: Explain in detail why a physical exam is necessary and how it can be used to determine the most effective treatment plan. Make sure the patient understands that these exams are meant to help them, not to judge or stigmatize them.

4. **DESCRIBE THE EXAMINATION PROCESS**: Explain the specific steps of the examination so that the patient understands the process during. Understanding the process may reduce fear and anxiety.

5. **Provide privacy guarantees**: emphasize that all information and test results will be kept confidential and used only for medical purposes.

6. **Encourage patients to participate in decision-making**: make patients feel that they are in control of their treatment by asking for their input, allowing them to ask questions, and considering their needs and preferences.

7. **Provide psychological support**: If needed, consider recommending a psychologist for more intensive psychological support to help patients overcome their concerns and fears.

8. **Emphasize teamwork**: Let patients know that the physician and the entire healthcare team are on the same page with them, working together to improve their health.

9. **Give Time**: If patients need time to think, don't force them to make an immediate decision. Provide enough time and support to allow them to make their own decisions.

Through these steps, physicians can reduce patients' fear and anxiety, help them understand the importance of physical examination, and enhance their cooperation and trust. This requires the practitioner to have good communication skills and empathy in order to individualize care and support to the patient.

15. Q：When male patients worry that their sexual dysfunction will affect their partner's satisfaction and thus develop low self-esteem or avoidance, how can physicians encourage and support them?

A：When male patients worry that their sexual dysfunction will affect their partner's satisfaction and consequently develop low self-esteem or avoidance, physicians can take the following measures to encourage and support them:

1. **Establish a trusting relationship**: Communicate with patients in an open, empathetic and non-judgmental manner so that they feel safe and understood.

2. **Understand deep concerns**: gain insight into the patient's specific concerns, feelings, and expectations through open-ended questions and listening.

3. **Educate and explain**: explain to patients that sexual dysfunction is a common medical problem and share with them information about its causes, diagnosis, and treatment.

4. **Emphasize the importance of partner communication**: Encourage patients to share feelings and concerns with their partners and emphasize the importance of communication and understanding for a satisfying sexual relationship.

5. **Provide treatment options**: explain available treatment options, including medications, psychotherapy, and physical therapy, and discuss the positive impact they may have on the patient's and partner's relationship.

6. **Encourage realistic expectations**: help the patient understand that improvement may take time and effort and may involve trial and error.

7. **Consider partner involvement**: if the patient agrees, consider inviting the partner to participate in discussions or therapy to promote shared understanding and support.

8. **Referral to professional psychological support**: If the situation is complex or deep psychological issues are involved, referral to a professional mental health professional, such as a sexual health psychotherapist, may be appropriate.

9. **Establish an ongoing support system**: ensure that patients know they can contact their physician or other support person at any time to establish an ongoing supportive relationship.

10. **Emphasize overall health and well-being**: Help patients see that sexual health is only one part of overall health and well-being and encourage them to seek balance and fulfillment in other areas of their lives.

Through these steps, practitioners can help patients understand that sexual dysfunction does not equate to failure or incompleteness and can be improved with appropriate intervention and support. It is important to communicate with patients in an empathetic and respectful manner to promote genuine dialog and therapeutic collaboration.

16. Q：When male patients are reluctant to talk about their sexual function for fear of being ridiculed or criticized by their partners, family members or society, how should physicians reassure and encourage them?

A：When male patients are reluctant to talk about their sexual function for fear of being ridiculed or criticized by their partners, family members or society, physicians can take the following measures to reassure and encourage them:

1. **Create a safe environment**: Ensure that the conversation with the patient takes place in a private, stress-free environment where the patient knows that all information will be kept confidential.

2. **Demonstrate empathy**: reduce the patient's anxiety and defenses by demonstrating understanding and compassion.

3. **Provide education and information**: explaining the commonality of sexual dysfunction and that it can be treated in several ways helps to reduce stigma and isolation.

4. **Gradually guide**: patients may need time to become comfortable discussing the issue, so guide them gradually and don't force them to share all their concerns right away.

5. **Emphasize the importance of communication**: Encourage the patient to share their feelings and concerns with a partner or trusted family member, and possibly offer guidance or involvement to facilitate this conversation.

6. **Provide professional support**: if the patient is particularly nervous or worried about communicating with a partner or family member, the support of a sexual health psychotherapist or other professional may be recommended.

7. **Set a clear treatment plan**: work with the patient to develop a clear, individualized treatment plan so they know what will happen next and to reduce uncertainty.

8. **Reflect social realities**: help patients understand that social perceptions of sexual functioning problems are changing, and that more and more people are recognizing that this is a medical problem, not a personal failure.

9. **Ongoing follow-up and support**: ensuring that patients know that they can contact their physician at any time demonstrates ongoing concern and support, which can increase patient comfort and trust.

By doing so, the practitioner can reduce the patient's anxiety and stigma and encourage them to positively confront and deal with their sexual functioning. The key is to demonstrate empathy and patience throughout the process and ensure that patients feel supported and understood.

17. Q：When male patients worry that their sexual dysfunction problems will lead to their partners leaving or being unfaithful, how should physicians advise and help them deal with this concern?

A：When male patients worry that their sexual dysfunction will cause their partner to leave or be unfaithful, this concern may further exacerbate the patient's anxiety and stress. Physicians can take the following steps to help and advise them in dealing with this concern:

1. **Listening and understanding**: first, give patients ample time and space to express their concerns and feelings, demonstrating true understanding and empathy.

2. **Educate and explain**: explaining sexual dysfunction is a common medical problem experienced by many people. It is important for patients to understand that it is not their fault and is not an insurmountable obstacle.

3. **Encourage partner communication**: if possible, encourage the patient to discuss the issue with their partner. Sometimes open and honest communication between couples can eliminate many unnecessary concerns and misunderstandings.

4. **Provide professional counseling**: Consider recommending that patients and their partners receive counseling or sex therapy together. Sometimes professional third-party intervention can help to resolve deep-seated communication and trust issues.

5. **Develop an individualized treatment plan**: Work with the patient to develop a treatment plan that works for them and explains each step in detail to alleviate their uncertainties and fears.

6. **Emphasize overall relationship health**: Emphasize that sex is not the only important aspect of a relationship and encourage patients to consider other ways to increase intimacy and trust.

7. **Ongoing support and follow-up**: Stay in touch with the patient and provide ongoing support and encouragement. Their confidence and relationship may improve as treatment progresses.

8. **Provision of resources and materials**: Sometimes providing printed or online materials about sexual dysfunction and effective treatment can also help patients and their partners better understand the problem.

By doing the above, practitioners can help patients understand that sexual dysfunction is not the entirety of who they are, nor does it necessarily lead to a partner leaving or being unfaithful. The key is to show empathy, provide correct information, and encourage the patient and partner to work together to solve the problem. Such efforts may not only help resolve the sexual dysfunction but may also enhance their overall relationship.

18. Q：How can physicians convince male patients to seek help when they are experiencing intense anxiety and depression due to sexual dysfunction but fear that psychotherapy will be perceived as "weak"?

A：Physicians face a sensitive and complex challenge when male patients experience intense anxiety and depression due to sexual dysfunction but fear that psychotherapy will be perceived as "weak". Such situations require careful communication and support, and the following are some possible ways to convince patients to seek help:

1. **Demonstrate empathy and understanding**: The first thing to do is to show understanding and concern for the patient's feelings and ensure that they feel safe to share their concerns.

2. **Dispelling stereotypes**: articulate the importance of mental health and dispel misconceptions and stereotypes about psychotherapy. It can be emphasized that seeking psychological help is not a sign of weakness, but a form of positive and responsible self-care.

3. **Educate on the importance of mental health**: explain how mental health is linked to overall health and how it affects sexual functioning. Clarify that psychotherapy is a legitimate medical intervention and is as important as physical therapy.

4. **Provide appropriate resources and information**: provide information about psychotherapy, including its benefits, process, and possible outcomes. This helps to eliminate the patient's fear of the unknown.

5. **Share success stories**: If appropriate, sharing some success stories of treatment (ensuring confidentiality) can help boost the patient's confidence in treatment.

6. **Introduce gentle introductory options**: for example, suggest that the patient first have an informal conversation with a psychologist or explore some online mental health resources and self-help tools.

7. **Emphasize privacy and confidentiality**: Emphasizing the confidentiality of psychotherapy may help to alleviate the concerns of patients who may be worried about others knowing they are receiving psychotherapy.

8. **Planning with the patient**: Invite the patient to participate in the development of the treatment plan and clarify their roles and responsibilities throughout the process.

9. **Ongoing support and follow-up**: Make sure the patient knows that you will be there to provide support throughout the treatment process and be available to answer any questions they may have.

10. **Introduce family support**: if the patient agrees, consider working with the family to promote a more supportive home environment.

By taking these steps, practitioners can help patients overcome concerns about psychotherapy, understand its importance, and encourage them to take positive steps to improve their mental health and overall quality of life.

19. Q：When male patients begin to avoid all forms of intimacy, even simple physical touch, because of concerns about their sexuality, how should physicians encourage them to rebuild intimacy with their partners?

A：When male patients begin to avoid all forms of intimacy, even simple physical touch, because of concerns about their sexuality, physicians may use the following strategies to encourage them to rebuild intimacy with their partners:

1. **Build trust and understanding**: The practitioner needs to establish a solid relationship of trust with the patient, demonstrate understanding of their concerns, and emphasize that the treatment process is a collaborative effort.

2. **Educate patients about the diversity of intimacy**: Emphasize that intimacy is about more than just sex. Discussing sharing feelings with your partner, hugging, kissing, etc. are all important parts of intimacy.

3. **Encourage open communication**: Advise the patient to discuss their feelings and concerns openly with their partner and provide some communication tips and strategies.

4. **Gradually increase physical contact**: Provide gradual exercises and suggestions that the patient can experiment with his/her partner, e.g., start with a handshake, hug, and gradually increase the intimacy of physical contact.

5. **Assist in setting reasonable goals**: Set achievable goals with the patient and slowly work towards rebuilding intimacy.

6. **Referral to couples or sex therapy**: If needed, a professional couples therapist or sex therapist can be brought in to provide more specialized and targeted assistance.

7. **Emphasize partner's supportive role**: Work with the patient's partner to explain their important role in the treatment process and provide support and encouragement.

8. **Follow-up and Support**: Through regular follow-up, understand the patient's progress and feelings, and provide support and encouragement as needed.

9. **Provide information and resources**: Provide books, articles, or online resources on intimacy building for further understanding and learning.

10. **Emphasize patience and gentleness**: Encourage patients to be patient with both them and their partners, recognizing that rebuilding intimacy may be a gradual process.

In these ways, practitioners can help male patients understand and value the many facets of intimacy and encourage them to take positive steps to overcome their concerns and rebuild an intimate connection with their partner. This can take time and effort, but with the right guidance and support, most patients are able to make progress in this area.

20. Q：When male patients are afraid to discuss their sexual functioning with their doctors for fear of being misinterpreted or judged, what can physicians do to reassure and build their trust?

A：When male patients are afraid to discuss their sexual functioning with their doctors for fear of being misunderstood or judged, physicians can take the following steps to reassure and build their trust:

1. **Demonstrate empathy and professionalism**: Communicate to the patient that their problem is being treated as a serious and important medical issue by demonstrating understanding, respect, and compassion.

2. **Ensure Privacy**: Clearly communicate to the patient that all discussions and information will be kept confidential and follow the rules of medical confidentiality.

3. **Provide a comfortable environment**: Ensure the privacy of the consultation room and provide a comfortable setting to reduce the patient's tension and anxiety.

4. **USE PROFESSIONAL LANGUAGE**: Avoid language that could be interpreted as judgmental or demeaning, and insist on the use of neutral and professional medical terminology.

5. **EXPLAIN THE COMMONNESS OF SEXUAL DYSFUNCTION**: Explain to the patient how common sexual dysfunction is in men to reduce their feelings of embarrassment or shame.

6. **DETAILED HISTORY TAKING**: Demonstrating attention to the problem and professionalism through thorough and detailed history taking helps to enhance patient trust.

7. **Provide adequate time and patience**: Don't be in a hurry to push the discussion; give the patient enough time to express his/her concerns and feelings.

8. **Encourage questions**: By encouraging patients to ask questions, they feel more in control of their treatment.

9. **Explain treatment options**: Clearly explain possible treatment options, including their effects and side effects, so that patients can make more informed decisions.

10. **Referral to other resources**: If appropriate, refer the patient to a specialized sexual health therapist for help.

11. **Follow-up support**: Provide ongoing support and let patients know that you are their partner throughout the treatment process through regular follow-up.

By using the above strategies, physicians can allay male patients' concerns when discussing sexual functioning and help them understand that this is a normal medical issue that can be discussed openly with healthcare professionals. The ultimate goal is to motivate patients to feel safe and comfortable communicating with their physicians in order to better diagnose and treat their problems.

21. How should physicians reassure and convince patients when they are reluctant to undergo the necessary rehabilitation for fear of post-surgical pain?

When patients are reluctant to undergo the necessary rehabilitation training because they are worried about post-surgical pain, physicians can take the following measures to reassure and convince them:

1. **Fully understand the patient's concerns**: Show understanding and empathy by first listening to the patient's concerns and understanding their specific fears and worries about pain.

2. **Provide pain management options**: explain to the patient the pain management measures available, including pharmacologic and non-pharmacologic treatments, such as physical therapy, to alleviate discomfort during rehabilitation training.

3. **EXPLAIN THE IMPORTANCE OF REHABILITATION**: Clearly explain the importance of rehabilitation in restoring function, minimizing complications, and improving quality of life.

4. **Set Reasonable Expectations**: Work with the patient to set realistic and achievable rehabilitation goals, avoiding overly optimistic or unrealistic promises.

5. **Take training step by step**: Propose a progressive rehabilitation program, starting with simple and comfortable exercises and gradually increasing the intensity to reduce the patient's fear.

6. **Provide psychological support**: If needed, consider working with a mental health professional to provide additional emotional support and anxiety relief for the patient.

7. **INVITE FAMILY SUPPORT**: Communicate with the patient's family members to explain the importance of their support and involvement in the recovery process.

8. **Showcase Success Stories**: If possible, contact patients who have successfully recovered and ask them to share their experiences to boost patients' confidence.

9. **Maintain transparent communication**: Communicate with patients on a regular basis to understand their recovery progress and any concerns about pain, and make timely adjustments to the rehabilitation program.

10. **Regular Assessment of Progress**: Keep patients informed of their progress and the importance of continuing their efforts through regular assessments and feedback.

11. **Provide educational materials**: Provide written or video educational materials to keep patients informed about the stages of the rehabilitation process and why each stage is necessary.

By doing the above, physicians can help patients understand the importance of rehabilitation training and provide enough support and confidence so that they are willing to actively participate in the necessary rehabilitation training. This comprehensive approach helps to alleviate patients' fears and doubts and motivates them to work towards a better recovery.

22. What should physicians do to encourage patients when they are embarrassed by the physical changes caused by urinary diversion and refuse to change clothes or display their bodies in public?

When patients are embarrassed by the physical changes caused by urinary diversion (e.g., urostomy) and refuse to change or display their bodies in public, physicians can take the following steps to encourage and support them:

1. **Understanding and empathy**: Always communicate with the patient in an understanding and empathetic manner, fully recognizing the importance of the change in body image to the patient and understanding their feelings.

2. **Education and Explanation**: clearly explain the purpose and importance of urinary diversion surgery and how to live a normal life. Information and training on post-operative care and daily management can be provided.

3. **Demonstrate concealment options**: Show patients the concealment methods and tools available, such as special undergarments or coverings, to help them feel more comfortable in public.

4. **Gradually Increase Confidence**: Encourage patients to gradually increase their comfort level in public, starting with small and familiar settings.

5. **Provide psychological support**: Consider bringing in the help of a psychologist or social worker to address the patient's emotional issues in more depth.

6. **INVITE PEER SUPPORT**: If possible, connect the patient with people who are going through the same experience and allow them to share their experiences and support each other.

7. **SET ACTUAL GOALS**: Work with the patient to set specific, measurable goals so they can gradually adjust to their new body image.

8. **Emphasize other aspects of the body**: Work with patients to explore other positive attributes of their body and appearance and to emphasize that the surgery does not change their overall value and identity as a person.

9. **Encourage participation in social activities**: Encourage patients to gradually engage in social activities that they enjoy in order to enhance their self-confidence and social skills.

10. **Ongoing Attention and Support**: Continue to stay in touch with the patient throughout the recovery process to provide ongoing support and encouragement.

11. **Provide specialized resources and community support**: Inform patients about urinary diversion support groups and resources so they can connect with others who have had similar experiences.

By doing so, physicians can gradually help patients adjust to their new body image and encourage them to feel more confident and comfortable participating in public. This is not only a medical issue, but also involves the psychological and social needs of the patient, so a multidisciplinary approach may be needed to provide a full range of support.

23. What can physicians do to encourage and support patients when they are concerned that their urinary flow pattern after diversion will cause them to avoid socializing by being looked at differently in public places?

When patients are concerned that the way their urine flows after diversion will cause them to be looked at differently in public places and thus avoid socializing, the physician can encourage and support the patient through the following strategies:

1. **Education and training**: Explain in detail the need for diversion and provide training on how to manage and cover the urinary diversion after surgery. Provide practical tips and tools so that patients can easily manage it when they are away from home.

2. **Provide practical products**: Tell patients about concealment products designed specifically for diversion patients, such as special underwear, coverings, etc., to help them feel more comfortable.

3. **Empathy and Encouragement**: Demonstrate understanding of the patient's concerns and encourage them to participate in social activities, emphasizing that reorientation does not change their value as human beings.

4. **Gradual Transition**: Help the patient set up a plan for gradual participation in social activities, starting with small, familiar settings and gradually increasing complexity and public exposure.

5. **Psychological Intervention**: If needed, bring in the assistance of a mental health professional to address possible anxiety or low self-esteem through counseling or therapy.

6. **PEER SUPPORT**: Connects the patient with people or support groups who share the same experience so that they can share experiences, encourage each other, and understand.

7. **Emphasize the positive**: Work with the patient to emphasize their strengths and hobbies, encouraging them to re-engage in life and social activities.

8. **Encourage support from family and friends**: Educate family members and friends on how to provide emotional and practical support and encourage them to participate in social activities with the patient.

9. **Ongoing follow-up**: Communicate regularly with the patient about their progress and any ongoing concerns, and provide ongoing support and encouragement.

10. **Provide community resources**: Provide information about support groups, activities, and other community resources that will help with their social integration.

By combining education, psychosocial support, social skills training, and ongoing attention, practitioners can help patients overcome concerns about diversionary surgery and return to active participation in social activities. Such an integrated approach can effectively address patients' concerns and help them re-establish a confident and satisfying social life after surgery.

24. When patients avoid intimacy with their partners because they are concerned about their sexual attractiveness due to physical changes after surgery, what should physicians do to help them build their self-confidence?

When patients are concerned about their sexual attractiveness because of physical changes after surgery, and thus avoid intimacy with their partners, physicians can help build their confidence in the following ways:

1. **Conducting education and communication**: Begin by explaining to the patient the need for the procedure and the expected recovery process after surgery. Help patients understand that while appearance may change, this does not necessarily affect their intimate relationship with their partner.

2. **Encourage Partner Involvement**: If possible, encourage the patient's partner to participate in the discussion and support the process. Sometimes a partner's understanding and acceptance can go a long way to alleviating a patient's concerns.

3. **Psychological support**: If the patient's concerns persist or increase, consider referring the patient to a mental health professional for counseling or treatment. Professional psychological support can help address deep-seated anxiety and low self-esteem.

4. **Emphasize well-rounded personalities and values**: Work with patients to explore their other strengths and attractions that go beyond physical appearance.

5. **Provide practical solutions**: If there are specific concerns about appearance, such as scars, deformities, etc., the practitioner can provide viable solutions, such as rehabilitation therapy, counseling on plastic surgery, etc.

6. **Promote intimate communication skills**: Specialized communication skills training may be offered or recommended to teach patients how to communicate their feelings and concerns to their partners.

7. **Establishing a progressive intimacy program**: Help the patient and partner to develop a progressive program to gradually re-establish intimacy. Start with non-sexual physical touch and emotional communication and gradually progress to deeper levels of intimacy.

8. **Provide resources and information**: Provide educational resources and information about recovery, body image, sexual health, etc. to help patients gain a more comprehensive understanding.

9. **Ongoing follow-up**: Check in with patients regularly to see how they are progressing in rebuilding intimacy so that support and intervention can be provided in a timely manner.

10. **Encourage participation in support groups**: If possible, introduce the patient to a group of others who are going through similar experiences so that they can share experiences and receive support.

Through a combination of education, communication, psychosocial support and practical interventions, practitioners can effectively help patients overcome concerns about sexual attraction brought about by physical changes after surgery and help them re-establish intimacy with their partners. This not only contributes to their physical healing, but also to emotional and psychological recovery and growth.

25. How should physicians advise and encourage patients when they refuse to leave their homes because they fear that a new urinary flow pattern may lead to leaks or accidents?

When patients refuse to leave their homes because they fear that the new urinary flow method may cause leaks or accidents, physicians can advise and encourage them by taking the following steps:

1. **Provide detailed education**: Fully explain how the new urinary flow modality works and common problems that may arise. Let patients know that their concerns are manageable and teach them how to properly use the medical equipment or products they need.

2. **Skills Training**: Provides patients with a better understanding and mastery of the new urinary flow modality through hands-on demonstrations and training to increase confidence.

3. **Encouragement of gradual trial**: Suggest short trips for patients starting from a close distance and gradually increasing the distance and time. This increases their confidence and adaptability to the new urinary flow modality.

4. **Provide contingency plans**: Plan possible contingency plans with the patient, such as carrying spare medical equipment, choosing locations with restrooms, etc., so they feel more prepared and in control.

5. **Psychological support**: If the patient's anxiety persists, consider offering the support of a mental health professional to explore and address their concerns in more depth.

6. **Facilitate support from family and friends**: Encourage patients to communicate their concerns and needs with family and friends and facilitate their understanding and support.

7. **Provide information about related products and services**: Special medical supplies, such as absorbent devices or special diapers, may be recommended to minimize leakage concerns.

8. **Follow-up and Ongoing Evaluation**: Through regular follow-up, learn about the patient's progress and challenges, and make timely adjustments to programs and support.

9. **Social support**: If possible, introduce the patient to others going through similar situations or to support groups so they can share experiences and solutions.

10. **Emphasis on quality of life**: Motivate patients to overcome their fears by emphasizing the importance of active participation in social activities and daily life for overall health and quality of life.

By doing so, physicians can help patients understand and adapt to their new urinary flow patterns, alleviate their concerns, and gradually encourage them to return to their daily lives. Importantly, this process may take time and patience in order for patients to gradually gain confidence and comfort.

26. How should physicians reassure and persuade patients when they are anxious or stressed about learning the necessary self-care skills because they are concerned about the need for post-operative care after diversion?

When patients are anxious or stressed about their care needs after diversion and are reluctant to learn the necessary self-care skills, practitioners can use the following strategies to reassure and persuade them:

1. **In-depth understanding of concerns**: Communicate with patients to understand their specific concerns and fears so that they can be answered and addressed more effectively.

2. **Provide detailed education and training**: Provide a detailed explanation of the process of care after diversion and teach the required skills step-by-step. Practical demonstrations and exercises can help patients better understand and master the process.

3. **Teach step-by-step**: Break down the care process into small, manageable steps and teach them step-by-step; don't give the patient all the information at the beginning to avoid causing stress.

4. **Provide supportive materials**: Create or provide easy-to-follow illustrations, videos, or manuals that patients can repeat and practice at home.

5. **Encourage family member involvement**: If possible, encourage family members to participate in the learning and support process so that the patient feels more supported.

6. **Set achievable goals**: Set achievable learning goals with the patient so they feel more in control and motivated.

7. **Provide Ongoing Support**: Ensure that patients know that they can seek support and help from their healthcare team at any time during their home care journey.

8. **Emphasize Importance and Benefits**: Clearly explain the importance of self-care skills for post-operative recovery and quality of life so that patients understand the long-term benefits of learning these skills.

9. **Increase self-confidence**: Gradually increase the patient's self-confidence and autonomy through positive feedback and encouragement.

10. **Arrangement of follow-up**: Ensure that the patient receives adequate support and guidance during the learning and practicing process through regular follow-ups.

11. **Mental health support**: If a patient's anxiety persists, consider working with a mental health professional to more fully address their emotional needs.

By using the above methods, physicians can build a trusting relationship with their patients, provide the support and guidance needed to help them overcome their fears and anxieties about self-care after rerouting, and inspire motivation and confidence in their learning. This process may require patience and empathy in order for the patient to gradually adapt and acquire the needed self-care skills.

27. What should physicians do to encourage patients to return to normal life when they develop strong feelings of denial about their daily life after diversion and thus refuse to participate in family or work activities?

When patients develop strong negativity about their daily life after diversion and thus refuse to participate in family or work activities, the practitioner may need to encourage and support them to return to their normal life by doing the following:

1. **Understanding Specific Concerns**: Have an in-depth conversation with the patient to find out what they are specifically worried about, whether it is adjustment to their new physical condition, concerns about their social life, or doubts about their ability to work, etc.

2. **Provide education and explanation**: Explain in detail the recovery process after diversion and how to gradually return to a normal family and work life.

3. **Set progressive goals**: Help patients to set progressively increasing goals, starting with simple daily tasks and gradually increasing the level of difficulty and participation, encouraging them to gradually re-engage in life.

4. **Provide vocational rehabilitation support**: If necessary, consider support from vocational rehabilitation services to help the patient re-adjust to the work environment and responsibilities.

5. **Psychological support and therapy**: Sometimes, patients may need professional mental health support to deal with negativity and regain a positive attitude. Psychotherapy or psychological support groups may be effective options.

6. **Encourage family support**: Encourage family members to participate in the recovery process with positive support and understanding.

7. **Showcase of Successful Cases**: Sometimes, showing patients the cases of other people who have successfully recovered and reintegrated into normal life can boost their confidence.

8. **Continuous monitoring and support**: Regularly follow up with the patient to assess progress, answer questions and provide necessary support.

9. **Promote social participation**: Encourage patients to gradually participate in social activities, such as friends' gatherings or interest groups, to alleviate the feeling of isolation.

10. **Emphasize positive quality of life**: Clearly express the likelihood that rehabilitation will lead to a return to normal life and work, emphasizing positive quality of life.

11. **Consider multidisciplinary teamwork**: Collaboration of a multidisciplinary team of physicians, nurses, social workers, psychologists, etc. may be helpful in providing a full range of support.

Through these strategies, physicians can encourage and support patients to overcome denial and gradually re-engage in family and work life. Importantly, this process may take time and patience in order for the patient to gradually build confidence and resilience. Physicians should maintain a compassionate and supportive attitude to ensure that patients know they are not alone in this process and have access to the support and resources they need.

28. How should physicians reassure and advise patients to seek help when they are depressed because of physical changes after surgery, but fear that they will be seen as "weak" if they talk to others?

The physician's role is to provide support, understanding, education, and encouragement when a patient is depressed because of the physical changes following surgery, but is concerned that talking to others will be seen as a "weakness". The following are some possible strategies:

1. **Build trust and confidentiality**: First, the practitioner should explicitly reassure the patient that all conversations are confidential and emphasize that his or her role is non-judgmental and supportive.

2. **DEMONSTRATE UNDERSTANDING AND SYMPATHY**: Show understanding and empathy for the patient's emotional response, emphasizing that physical changes and adjustments after surgery are a normal and common challenge.

3. **Dismantle the notion of "weakness "**: Expressing feelings clearly and asking for help is not a sign of weakness, but a common and healthy response. It is a common and healthy response that explains that many people may need support and help in similar situations.

4. **Provide educational information**: explains that depression is a treatable medical condition and not a sign of personal weakness or failure. Provide information about depressive symptoms and possible treatments.

5. **Encourage professional help**: Emphasize that seeking help from a mental health professional is a reasonable and effective step. Provide appropriate resources and referrals.

6. **Promote family and friend support**: Encourage the patient to share their feelings with a trusted family member or friend, and may also provide guidance on how to communicate with others.

7. **Set up a follow-up plan**: Work with the patient to develop a specific action plan, including possible follow-up dates, goals, etc., and emphasize that you will continue to support them.

8. **CONSIDER MEDICATION SUPPORT**: If the situation is serious, discuss the possibility of medication, explaining how it works and how it might work.

9. **Emphasize a positive outlook for recovery**: Emphasize that many people successfully adapt to physical changes and return to active lives with appropriate support and treatment.

10. **Provide Self-Help Strategies**: Self-help strategies that may help improve mood, such as exercise, good eating and sleeping habits, etc., may also be presented.

By doing so, the practitioner can work to allay the patient's concerns and encourage them to seek the necessary support and help. Practitioners need to maintain open and non-judgmental communication to provide patients with clear information and workable solutions to support their mental health needs during the recovery process.

29. When patients are afraid to discuss their post-surgical quality of life or psychological problems with their physicians or health-care providers, how can physicians build their trust and encourage them to open up their communication?

The process of building trust and facilitating open communication is important when patients are afraid to discuss quality of life or psychological issues after surgery with their doctor or healthcare provider. Here are some possible strategies:

1. **Demonstrate Sincere Concern**: Demonstrate concern for the patient's overall well-being, not limited to the physical condition, through body language, verbalization, and sincerity.

2. **Ensure Privacy and Confidentiality**: Clearly inform the patient that all discussions will remain confidential and ensure that the conversation takes place in a private setting.

3. **Encourage open-ended questions**: Guide patients to share their feelings and concerns through open-ended questions such as "How has your life changed since your surgery?" rather than the closed-ended "Do you feel good?" .

4. **Emphasize a holistic view of health**: Emphasize that mental health and quality of life are important components of a holistic health plan, just as important as physical health.

5. **Set Clear Expectations**: Explain that discussing these issues is a routine part of care and contributes to more comprehensive, individualized care.

6. **Demonstrate patience and listen**: Take the time to listen to the patient's concerns and feelings rather than rushing to provide solutions. Sometimes being heard and understood is an important support in itself.

7. **Provide information and resources**: Provide clear information and resources for possible concerns and problems, explaining possible solutions and support.

8. **Establish follow-up mechanisms**: Set up mechanisms for regular check-ins and communication to ensure that patients know that they are always available to discuss these issues with healthcare professionals.

9. **Collaborative Care**: If possible, establish a collaborative care team, including mental health professionals, etc., to fully support the patient's needs.

10. **Encourage Patient Participation in Decision Making**: Encourage patients to participate in the treatment planning and decision making process so that they feel in control and empowered.

11. **Provide self-care strategies**: Self-care strategies that may help improve psychological and quality of life can be taught and encouraged.

12. **Positive Affirmation and Support**: Acknowledging and supporting the patient's efforts and progress increases their self-confidence.

By doing so, the practitioner can work to allay the patient's fears and concerns and promote more open and helpful communication. This requires patience, empathy and expertise to ensure that patients feel comfortable and safe sharing their feelings and needs.

30. How should physicians reassure and persuade patients when they refuse to undergo necessary medical examinations or treatments due to fear of re-operation or treatment that may be required after surgery?

When a patient develops a fear of another surgery or treatment that may be required, and thus refuses to undergo the necessary medical tests or treatments, the physician and the healthcare team need to take a holistic and compassionate approach to understand, reassure, and convince them. Here are some possible strategies:

1. **In-depth understanding of the patient's concerns**: First, it is important to understand why the patient is concerned or afraid of another procedure or treatment. Is it fear of pain, fear of cost, fear of unknown outcomes or some other reason? Only when you truly understand their concerns can you respond effectively.

2. **Provide complete information**: Explain the need for reoperation or treatment, the process, and the expected outcome in a way that the patient can understand. Emphasize why this is the best or only option and explain the possible consequences if it is not performed.

3. **Discusses risks and benefits**: Clearly explains the potential risks and benefits of the surgery or treatment and how the health care team plans to mitigate those risks.

4. **Provide support and comfort**: Ensure that the patient knows that the healthcare team will fully support them, including post-operative pain management, rehabilitation support, etc.

5. **Present success stories**: If appropriate, share success stories of similar surgeries or treatments with patients, or even connect them with patients who have already gone through a similar process.

6. **Involve family and support networks**: If the patient agrees, family or support people can be included in the discussion so that they too can understand the situation and support the patient.

7. **Consider patient choices**: Where possible, involve the patient in the treatment decision-making process and consider their choices and preferences.

8. **Provide psychological support**: may require support from a mental health professional if the patient's fears are symptoms of severe anxiety or fear.

9. **Develop a clear plan**: If the patient agrees to the procedure or treatment, develop a clear plan to minimize uncertainty and fear.

10. **Keep lines of communication open**: Encourage the patient to ask questions or express concerns at any time and ensure that the healthcare team is always available to answer questions or provide support.

11. **Gentle Persuasion**: If the patient remains concerned, consistently and gently persuade, explain why treatment is necessary, and reiterate support.

Through these strategies, the practitioner can work to allay the patient's fears in a compassionate and respectful manner, encouraging them to undergo the necessary tests or treatments. This may take time, patience, and interdisciplinary collaboration to ensure that the patient receives the full range of support and understanding.

**Psychosocial support capacity results for ChatGPT 3.5**

1. Q：Patients with urinary incontinence are often depressed because of the shame they feel. How should the physician go about adjusting the patient's state of mind?

A: Managing urinary incontinence and addressing the associated feelings of shame and depression requires a holistic approach that involves both medical and psychological support. Here are some steps that a physician can take to help adjust a patient's state of mind:

1. Create a Supportive and Non-Judgmental Environment:

- It's crucial to establish trust and make the patient feel comfortable discussing their condition. Ensure that the patient knows they can talk openly about their concerns without fear of judgment.

2. Provide Education:

- Educate the patient about urinary incontinence, its causes, and potential treatments. Understanding the condition can help reduce anxiety and feelings of shame.

3. Explore Treatment Options:

- Discuss various treatment options tailored to the patient's specific situation, such as lifestyle modifications, pelvic floor exercises, medications, or surgical interventions. Addressing the physical aspect of incontinence can significantly improve the patient's quality of life.

4. Refer to Specialists:

- If necessary, refer the patient to specialists like urologists, gynecologists, or physical therapists who can provide more focused care and expertise in managing urinary incontinence.

5. Encourage Self-Care:

- Encourage patients to practice self-care, which includes maintaining a healthy lifestyle with regular exercise, a balanced diet, and adequate hydration. These habits can positively impact their condition and overall well-being.

6. Recommend Support Groups:

- Suggest joining support groups or counseling sessions, where patients can connect with others who are experiencing similar challenges. Sharing experiences can help reduce feelings of isolation and shame.

7. Address Psychological Well-being:

- Recognize the emotional impact of urinary incontinence. Inquire about the patient's mental health and provide appropriate referrals to mental health professionals or therapists who specialize in managing depression and anxiety.

8. Behavioral Therapies:

- Behavioral therapies, such as cognitive-behavioral therapy (CBT), can help patients develop coping strategies and change negative thought patterns related to their condition.

9. Medications:

- In some cases, medication may be prescribed to manage symptoms or underlying causes of incontinence. Discuss the potential benefits and side effects of any prescribed medications.

10. Monitor Progress:

- Regularly follow up with the patient to track their progress, adjust treatment plans if necessary, and provide ongoing emotional support.

11. Empower the Patient:

- Encourage the patient to take an active role in their treatment and management. Providing them with a sense of control over their condition can improve their self-esteem and reduce feelings of shame.

12. Address Body Image Concerns:

- Some patients may have body image issues related to incontinence. Offer resources or referrals to specialists who can address these concerns and help improve body confidence.

Remember that every patient is unique, and their needs may vary. Tailor your approach to each individual's situation and consider involving a multidisciplinary team of healthcare professionals to provide comprehensive care. Ultimately, the goal is to help patients regain their confidence, improve their quality of life, and manage any associated emotional challenges.

2. Q：How should physicians explain to patients when they refuse incontinence treatment because they are concerned about possible side effects?

A: When a patient refuses incontinence treatment due to concerns about potential side effects, it's essential for physicians to engage in open and empathetic communication. Here's a step-by-step approach on how to explain the situation to the patient:

1. **Listen Actively**: Begin by listening carefully to the patient's concerns. Give them ample time to express their worries and fears related to the treatment and its potential side effects. Show empathy and understanding throughout the conversation.

2. **Acknowledge Their Concerns**: Start by acknowledging the patient's concerns and validating their feelings. You might say, "I understand that you have concerns about the possible side effects of the treatment, and it's perfectly normal to feel this way."

3. **Provide Information**: Explain the treatment options in detail, including the potential side effects. Use plain language and avoid medical jargon. Present the information in a balanced manner, highlighting both the benefits and risks of the treatment.

4. **Answer Questions**: Encourage the patient to ask questions and address any misconceptions they may have. Be prepared to provide evidence-based information and cite relevant research or clinical data to support your explanation.

5. **Discuss Alternatives**: If there are alternative treatments with fewer side effects or non-invasive options, discuss them with the patient. Present the pros and cons of each alternative, allowing the patient to make an informed choice.

6. **Assess Risk-Benefit Ratio**: Help the patient understand the risk-benefit ratio of the treatment. Discuss how the potential benefits of managing incontinence may outweigh the risks of side effects, especially in terms of improving their quality of life.

7. **Personalize the Conversation**: Tailor your explanation to the patient's specific situation and concerns. For instance, if they have particular worries about certain side effects, address those directly.

8. **Provide Reassurance**: Offer reassurance that you will closely monitor the patient's progress during treatment and promptly address any side effects or concerns that may arise. Emphasize that the medical team is there to support them throughout the process.

9. **Involve Shared Decision-Making**: Engage the patient in shared decision-making. Encourage them to take an active role in their healthcare by weighing the risks and benefits and making a choice that aligns with their values and preferences.

10. **Respect Autonomy**: Ultimately, respect the patient's autonomy and decision-making process. If the patient still chooses not to proceed with the treatment after receiving comprehensive information and support, document their decision and continue to provide care consistent with their choices.

11. **Follow Up**: Schedule follow-up appointments to monitor the patient's condition and their overall well-being. This ongoing care can provide opportunities to revisit treatment options if the patient's circumstances or preferences change.

12. **Provide Resources**: Offer educational materials or recommend reputable sources where the patient can find additional information about their condition and treatment options.

Remember that patients have the right to make decisions about their own healthcare, even if those decisions differ from what a healthcare provider might recommend. The key is to provide them with the information and support they need to make informed choices that align with their values and concerns.

3. Q：How should physicians or psychologists help patients when they are afraid of establishing intimate relationships with people due to the psychological trauma caused by incontinence?

A: Fear of establishing intimate relationships due to psychological trauma caused by incontinence can significantly impact a patient's quality of life. Physicians, psychologists, and other healthcare professionals can play a crucial role in helping patients address these concerns. Here are some steps to assist patients in managing this fear:

1. **Open and Non-Judgmental Communication**:

- Create a safe and non-judgmental environment where patients feel comfortable discussing their fears and emotions. Ensure they know that their concerns are valid and that you are there to support them.

2. **Provide Education**:

- Educate the patient about incontinence, its causes, and available treatments. Explain that incontinence is a medical condition and not a reflection of their worth as a person. Knowledge can help demystify the condition and reduce fear.

3. **Address Psychological Trauma**:

- Acknowledge the psychological trauma associated with incontinence. Encourage the patient to express their feelings and experiences related to the trauma. Consider referring them to a mental health professional with expertise in trauma if necessary.

4. **Counseling and Psychotherapy**:

- Refer the patient to a psychologist or therapist who specializes in dealing with trauma, body image issues, and relationships. Therapy can provide a safe space to explore and address the emotional impact of incontinence.

5. **Cognitive-Behavioral Therapy (CBT)**:

- CBT techniques can help patients challenge and reframe negative thought patterns and beliefs related to their condition. It can empower them to develop coping strategies and improve self-esteem.

6. **Support Groups**:

- Suggest joining support groups or therapy groups specifically for individuals dealing with incontinence-related issues. Connecting with others who have similar experiences can reduce feelings of isolation and offer a sense of community.

7. **Intimacy and Relationship Counseling**:

- Refer the patient to a counselor or therapist who specializes in intimacy and relationship issues. This can help them address concerns about forming or maintaining intimate relationships.

8. **Self-Esteem Building**:

- Help the patient work on building their self-esteem and self-confidence. Encourage them to focus on their strengths, interests, and qualities that have nothing to do with their medical condition.

9. **Gradual Exposure and Desensitization**:

- Gradually expose the patient to situations that trigger their fear of intimacy, allowing them to become more comfortable over time. This can be done with the guidance of a therapist.

10. **Partner Involvement**:

- Encourage open communication between the patient and their partner. Provide resources or referrals for couples' counseling to address any relationship challenges that may arise.

11. **Medical Management**:

- Continue to address the physical aspect of incontinence through appropriate medical treatment and management. Successfully managing incontinence can improve the patient's self-esteem and reduce fear.

12. **Ongoing Support**:

- Maintain regular follow-up appointments to monitor the patient's progress and provide ongoing support. Adjust treatment plans or therapeutic approaches as needed.

It's essential to take a multidisciplinary approach, involving both medical and mental health professionals, to address the psychological trauma associated with incontinence and help patients regain their confidence and ability to form intimate relationships. Patience, empathy, and understanding are key components of this process.

4. Q：How can physicians encourage and support patients when they refuse to go out in public for fear of becoming incontinent?

A：Encouraging and supporting patients who refuse to go out in public due to the fear of becoming incontinent is essential for improving their quality of life and overall well-being. Here are some strategies that physicians can use to help patients overcome this fear:

1. **Open and Non-Judgmental Communication**:

- Create a safe and empathetic space for the patient to discuss their fears and concerns. Make it clear that their feelings are valid, and you are there to support them.

2. **Education**:

- Provide information about the patient's specific condition, its causes, and available treatments. Understanding the condition and its management can help alleviate fears.

3. **Set Realistic Goals**:

- Work with the patient to set achievable goals for gradually increasing their comfort level with going out in public. Start with short outings or trips to familiar and convenient places.

4. **Behavioral Therapy**:

- Cognitive-behavioral therapy (CBT) can be beneficial in addressing anxiety-related issues. A therapist can help the patient identify and change negative thought patterns and develop coping strategies for managing their fears.

5. **Exposure Therapy**:

- Gradual exposure to situations that trigger anxiety can be helpful. Encourage the patient to practice leaving their comfort zone in a controlled and supportive manner. Start with less challenging situations and progressively work towards more challenging ones.

6. **Support Groups**:

- Suggest joining support groups or therapy groups for individuals dealing with similar challenges. Sharing experiences with others can reduce feelings of isolation and provide a sense of community.

7. **Self-Management Techniques**:

- Teach the patient relaxation techniques, such as deep breathing exercises or mindfulness, to manage anxiety and panic in public settings.

8. **Medical Management**:

- Ensure that the patient's incontinence is well-managed with appropriate medical treatments, medications, or interventions. Improved control can boost their confidence when going out.

9. **Medication for Anxiety**:

- In some cases, patients may benefit from medications prescribed by a psychiatrist to help manage anxiety or panic disorders. Refer the patient to a mental health specialist for evaluation if needed.

10. **Accompaniment and Support**:

- Encourage the patient to have a supportive friend or family member accompany them when going out initially. Having someone they trust by their side can provide reassurance.

11. **Positive Reinforcement**:

- Praise and reward the patient for their efforts in facing their fears and going out in public. Positive reinforcement can motivate them to continue challenging themselves.

12. **Regular Follow-Up**:

- Schedule regular follow-up appointments to monitor the patient's progress. Celebrate their successes, no matter how small, and make adjustments to their treatment plan as needed.

13. **Accessibility Planning**:

- Help the patient plan outings with easy access to restroom facilities. Knowing that there are convenient restrooms nearby can alleviate anxiety.

14. **Empower Decision-Making**:

- Involve the patient in decisions about their treatment plan and exposure goals. Empower them to take control of their journey toward overcoming their fear.

Remember that progress may be gradual, and setbacks can occur. Patience, understanding, and ongoing support are crucial elements of helping patients overcome their fear of going out in public due to incontinence concerns. Collaboration with mental health professionals may also be beneficial in managing anxiety and phobias related to this fear.

5.Q：How should physicians build trust with patients when they fear that discussing incontinence will make them look weak and thus avoid communicating with their physicians?

A：Building trust with patients who fear discussing incontinence or any health concern due to worries about appearing weak is crucial for their well-being. Here are some strategies that physicians can use to foster trust and encourage open communication:

1. **Create a Safe and Non-Judgmental Environment**:

- Establish a welcoming and empathetic atmosphere during appointments. Ensure patients feel safe to discuss their concerns without fear of judgment or criticism.

2. **Active Listening**:

- Practice active listening by giving patients your full attention. Show empathy and understanding for their emotions and concerns. Use verbal and non-verbal cues to demonstrate that you are genuinely interested in their well-being.

3. **Normalize the Conversation**:

- Normalize discussions about incontinence and similar health issues. Let patients know that these concerns are common and not a sign of weakness. Share statistics or anecdotes about how many people experience similar problems.

4. **Emphasize Confidentiality**:

- Reassure patients that their medical information is kept confidential. Explain the importance of patient-doctor confidentiality, which allows for open and honest discussions without fear of personal information being shared.

5. **Educate About the Nature of Health Issues**:

- Provide information about incontinence and its causes. Explain that it is a medical condition that can affect people of all ages and backgrounds, emphasizing that it's not a reflection of personal strength or weakness.

6. **Emphasize Collaboration**:

- Frame the doctor-patient relationship as a partnership. Let patients know that you are there to work together to address their concerns and improve their health.

7. **Use Empowering Language**:

- Encourage patients to view seeking medical help as a sign of strength and self-care. Phrases like "taking charge of your health" or "making informed decisions" can help empower patients.

8. **Provide Reassurance**:

- Reassure patients that seeking help is a positive step towards improving their quality of life. Highlight the potential benefits of addressing incontinence, such as increased comfort and confidence.

9. **Share Success Stories**:

- Share stories or anecdotes of other patients who have successfully managed incontinence or similar issues. Hearing about real-life success can inspire hope and reduce stigma.

10. **Respect Autonomy**:

- Respect the patient's autonomy and decisions. Let them know that they have control over their healthcare choices, including whether or not to discuss and address incontinence.

11. **Follow Up and Support**:

- Schedule follow-up appointments to monitor progress and provide ongoing support. Demonstrating your commitment to their well-being can reinforce trust.

12. **Refer to Specialists**:

- If needed, refer patients to specialists, such as urologists or pelvic floor therapists, who can provide specialized care and further reduce the stigma associated with discussing incontinence.

13. **Continuing Education**:

- Stay up-to-date with the latest treatments and research related to incontinence. Being knowledgeable and confident in your ability to help patients can inspire trust.

Building trust with patients who have concerns about appearing weak when discussing incontinence may require time and patience. However, by fostering an open and supportive environment, providing education, and emphasizing collaboration, physicians can help patients feel more comfortable discussing and addressing their health issues.

6. Q：When patients isolate themselves at home because they are afraid that others will know about their incontinence problem, how should physicians or psychologists counsel them?

A： When patients isolate themselves at home due to the fear of others knowing about their incontinence problem, it's essential for physicians and psychologists to provide counseling and support to help them overcome this isolation. Here are some steps to consider:

1. **Initiate Open Communication**:

- Create a safe and non-judgmental space for the patient to discuss their feelings and concerns. Let them know that their feelings are valid, and you are there to help.

2. **Normalize Incontinence**:

- Educate the patient about incontinence and emphasize that it is a common medical condition that affects many people of all ages. Normalizing the issue can reduce stigma and shame.

3. **Discuss Emotional Impact**:

- Explore the emotional impact of isolation on the patient's mental health. Ask about their feelings of loneliness, depression, or anxiety related to their self-imposed isolation.

4. **Encourage Gradual Exposure**:

- Work with the patient to develop a plan for gradually reintegrating into social activities and outings. Start with small, manageable steps and gradually increase exposure to social situations.

5. **Cognitive-Behavioral Therapy (CBT)**:

- Consider referring the patient to a psychologist or therapist who specializes in CBT. CBT can help them identify and change negative thought patterns related to incontinence and social isolation.

6. **Address Social Anxiety**:

- If the patient is experiencing social anxiety, discuss strategies to manage and alleviate these symptoms. This may include relaxation techniques, exposure therapy, or medication, depending on the severity of the anxiety.

7. **Support Groups**:

- Suggest joining incontinence support groups or therapy groups where the patient can connect with others facing similar challenges. Peer support can be highly beneficial in reducing feelings of isolation.

8. **Medication for Anxiety or Depression**:

- If the patient is experiencing severe anxiety or depression, consider referring them to a psychiatrist for evaluation and potential medication management.

9. **Develop Coping Strategies**:

- Help the patient develop coping strategies for managing incontinence in social situations. This might include using protective products, finding accessible restrooms, or practicing relaxation techniques.

10. **Role-Playing and Desensitization**:

- Role-play different social scenarios with the patient to help them become more comfortable with potential challenges they may face when outside the home. Gradual desensitization can be part of this process.

11. **Involve Loved Ones**:

- Encourage the patient to share their concerns with trusted friends or family members who can provide emotional support and accompany them to social events if needed.

12. **Set Realistic Expectations**:

- Help the patient understand that progress may be gradual, and setbacks can occur. It's essential to set realistic expectations and celebrate small achievements along the way.

13. **Regular Follow-Up**:

- Schedule regular follow-up appointments to monitor the patient's progress and provide ongoing support. Adjust the treatment plan as needed based on their experiences and challenges.

14. **Empower Decision-Making**:

- Empower the patient to make choices about their social interactions and participation. Encourage them to take control of their journey toward social reintegration.

15. **Assess Safety and Physical Health**:

- Ensure that the patient's physical health and safety needs are met at home. Address any barriers to mobility or self-care that may be contributing to their isolation.

It's important to approach each patient's situation with empathy and patience, recognizing that overcoming isolation related to incontinence can be a challenging process. Collaboration with mental health professionals and the involvement of a support network can be instrumental in helping patients gradually regain their social life and confidence.

7. How should physicians or psychologists counsel patients when they isolate themselves at home because they are afraid that others will know about their incontinence problem?

A： When patients isolate themselves at home due to the fear that others will know about their incontinence problem, physicians and psychologists can play a critical role in counseling and supporting them. Here are some steps to effectively counsel and help these patients:

1. **Create a Safe and Supportive Environment**:

- Establish a trusting and non-judgmental atmosphere where the patient feels comfortable discussing their concerns. Ensure confidentiality to build trust.

2. **Active Listening and Empathy**:

- Listen attentively to the patient's feelings, fears, and experiences. Show empathy by acknowledging their emotions and demonstrating that you understand their distress.

3. **Normalize the Condition**:

- Educate the patient about incontinence as a common medical issue that affects people of all ages. Stress that they are not alone in dealing with this condition and that many individuals experience similar challenges.

4. **Discuss the Emotional Impact**:

- Explore the emotional impact of incontinence on the patient's life, including their social relationships, self-esteem, and mental health. Allow the patient to express their concerns without judgment.

5. **Identify Negative Beliefs**:

- Help the patient identify and challenge negative beliefs or cognitive distortions related to their condition. Encourage them to replace these negative thoughts with more realistic and positive ones.

6. **Social Anxiety Management**:

- If social anxiety is a significant factor, consider referring the patient to a therapist or counselor with expertise in anxiety disorders. Cognitive-behavioral therapy (CBT) or exposure therapy may be helpful in managing social anxiety.

7. **Gradual Exposure**:

- Collaborate with the patient to create a gradual exposure plan to re-integrate into social activities. Start with small and manageable social interactions, gradually increasing the level of exposure over time.

8. **Support Groups**:

- Encourage the patient to join incontinence support groups or therapy groups. Sharing experiences with others facing similar challenges can reduce feelings of isolation and provide a sense of community.

9. **Medication Evaluation**:

- If the patient's anxiety or depression is severe and significantly impacts their life, consider a referral to a psychiatrist for a medication evaluation. Medication may help manage symptoms while other strategies are implemented.

10. **Involve Family and Friends**:

- Encourage the patient to involve trusted friends or family members in their treatment and recovery process. These individuals can offer emotional support and accompany them to social events.

11. **Develop Coping Strategies**:

- Work with the patient to develop practical coping strategies for managing incontinence in social situations. Discuss the use of protective products, finding accessible restrooms, and maintaining a personal hygiene routine.

12. **Set Achievable Goals**:

- Help the patient set realistic and achievable goals for increasing social interactions. Celebrate their successes, no matter how small, to boost their self-confidence.

13. **Safety and Physical Health Assessment**:

- Ensure that the patient's physical health and safety needs are met at home. Address any mobility or self-care challenges that may contribute to their isolation.

14. **Regular Follow-Up**:

- Schedule regular follow-up appointments to monitor the patient's progress, address any obstacles, and adapt the treatment plan as needed.

15. **Empower Decision-Making**:

- Empower the patient to make choices regarding their social interactions and the pace of their reintegration into social life. Encourage them to take control of their journey toward reconnecting with others.

Helping patients overcome their fear of social isolation related to incontinence requires a patient-centered and compassionate approach. Collaboration between healthcare professionals and, if appropriate, mental health specialists, can significantly contribute to the patient's progress and well-being.

8. Q：How can physicians help patients when they have sleep disorders due to stress caused by incontinence problems?

A：Physicians can play a crucial role in helping patients who have sleep disorders due to stress caused by incontinence problems. Here are some steps and strategies to assist these patients:

1. **Open Communication**:

- Establish a safe and non-judgmental environment where patients feel comfortable discussing their incontinence-related stress and sleep problems. Encourage them to openly express their concerns.

2. **Evaluate the Incontinence Problem**:

- Conduct a thorough evaluation of the patient's incontinence problem to understand its severity, underlying causes, and any potential treatments or interventions that may help. Addressing the incontinence issue directly can alleviate stress.

3. **Stress Management Techniques**:

- Teach patients stress management techniques, such as relaxation exercises, deep breathing, meditation, or mindfulness. These techniques can help reduce the psychological impact of incontinence.

4. **Sleep Assessment**:

- Evaluate the patient's sleep patterns and quality by asking about their sleep habits, duration, and any specific sleep disturbances. Consider using validated sleep assessment tools or referring the patient to a sleep specialist for a comprehensive sleep study if needed.

5. **Medication Review**:

- Review the patient's current medications to identify any that may contribute to sleep disturbances or worsen incontinence. Adjustments or changes in medication may be necessary.

6. **Address Anxiety and Depression**:

- Assess the patient's mental health, as stress and incontinence can lead to anxiety and depression. If indicated, refer the patient to a mental health specialist for therapy or medication management.

7. **Incontinence Management**:

- Develop a personalized treatment plan to manage the incontinence problem. This may include lifestyle modifications, pelvic floor exercises, medications, or surgical interventions, depending on the underlying cause.

8. **Nocturia Management**:

- Address nocturia (frequent urination at night), which can disrupt sleep. This may involve limiting fluid intake before bedtime, addressing underlying medical conditions, or medication adjustments.

9. **Behavioral Therapy**:

- Cognitive-behavioral therapy (CBT) can be beneficial for patients experiencing stress-related sleep disturbances. CBT techniques can help patients manage anxiety and improve their sleep patterns.

10. **Sleep Hygiene Education**:

- Provide guidance on sleep hygiene practices, such as maintaining a regular sleep schedule, creating a comfortable sleep environment, and avoiding stimulants close to bedtime.

11. **Monitoring and Follow-Up**:

- Regularly monitor the patient's progress in managing both the incontinence and sleep issues. Adjust treatment plans as necessary and continue to provide support and guidance.

12. **Support Groups**:

- Encourage patients to join support groups or therapy groups for individuals dealing with incontinence and related sleep disturbances. Sharing experiences and coping strategies with others can be helpful.

13. **Prescribe Sleep Medications**:

- In some cases, short-term prescription sleep medications may be appropriate to help patients regain a regular sleep pattern while addressing the underlying incontinence issue. These medications should be used under close medical supervision.

14. **Coordinate Care**:

- Collaborate with other healthcare professionals, such as urologists, sleep specialists, and mental health experts, to provide comprehensive care and address all aspects of the patient's health.

15. **Educate on the Connection**:

- Explain to the patient the connection between incontinence-related stress and sleep disturbances. Understanding how these issues are interrelated can motivate the patient to address both simultaneously.

Helping patients with sleep disorders related to stress caused by incontinence problems requires a comprehensive and multidisciplinary approach. By addressing the underlying causes of stress and incontinence while providing support for improved sleep, physicians can significantly enhance the patient's overall well-being.

9. Q：How should physicians recommend alternative treatments to patients when they have persistent depression due to chronic incontinence problems but refuse medication for fear of its side effects?

A： When patients with chronic incontinence problems refuse medication for fear of its side effects but are experiencing persistent depression, physicians can recommend alternative treatments and approaches to address their mental health concerns. Here are some steps to consider:

1. **Engage in Open Dialogue**:

- Initiate a candid and empathetic conversation with the patient to understand their concerns about medication. Ensure they feel heard and validated.

2. **Educate About Depression and Incontinence**:

- Provide the patient with information about the relationship between depression and chronic incontinence, emphasizing that untreated depression can worsen their quality of life.

3. **Discuss the Benefits and Risks of Medication**:

- Review the potential benefits and side effects of medication for depression. Address the patient's specific fears and misconceptions about these side effects. Be honest and transparent.

4. **Offer Psychotherapy**:

- Suggest psychotherapy as an alternative or adjunct to medication. Cognitive-behavioral therapy (CBT) and other evidence-based therapies have been effective in treating depression.

5. **Recommend Support Groups**:

- Encourage the patient to join support groups or therapy groups focused on depression or incontinence. Sharing experiences with others can provide emotional support and coping strategies.

6. **Lifestyle Modifications**:

- Discuss the importance of lifestyle modifications, such as regular exercise, a balanced diet, and sufficient sleep, in managing depression. Offer guidance on how these changes can positively impact their mood.

7. **Mindfulness and Relaxation Techniques**:

- Teach mindfulness and relaxation techniques to help the patient manage symptoms of depression and anxiety. Techniques like deep breathing exercises and mindfulness meditation can be beneficial.

8. **Set Realistic Goals**:

- Collaborate with the patient to set achievable goals for managing their depression and incontinence. Break down these goals into smaller, manageable steps to prevent overwhelm.

9. **Physical Health Assessment**:

- Ensure that the patient's physical health is adequately managed. Address any underlying medical conditions contributing to their depression or incontinence.

10. **Regular Monitoring and Follow-Up**:

- Schedule regular follow-up appointments to monitor the patient's progress. Adjust treatment plans and interventions based on their response and needs.

11. **Involve a Mental Health Specialist**:

- Consider involving a mental health specialist, such as a psychiatrist or psychologist, to provide specialized treatment for depression. They can offer various therapeutic approaches and support.

12. **Psychoeducation**:

- Provide psychoeducation to help the patient understand depression as a medical condition that can be effectively treated. Knowledge about the condition can reduce stigma and fear.

13. **Respect Patient Autonomy**:

- Respect the patient's autonomy and their right to make decisions about their treatment. Collaboratively explore treatment options and respect their choices.

14. **Address Incontinence**:

- Continue to address the incontinence issue with non-pharmacological interventions, such as lifestyle modifications, pelvic floor exercises, or assistive devices. Successful management of incontinence can improve the patient's overall well-being.

15. **Family and Social Support**:

- Encourage the patient to involve trusted friends or family members in their support network. These individuals can provide emotional support and encouragement.

By offering a range of alternative treatments, addressing the patient's specific concerns about medication, and respecting their autonomy in treatment decisions, physicians can work with patients to find effective ways to manage depression in the context of chronic incontinence problems. Collaboration with mental health professionals can also be valuable in providing comprehensive care.

10. Q：When patients choose to remain silent for fear of being ridiculed or criticized by family or friends for speaking out about their incontinence, how can physicians reassure and encourage them?

A: Patients who choose to remain silent about their incontinence due to fear of ridicule or criticism from family or friends may require additional reassurance and encouragement from physicians to open up about their condition. Here are some strategies to help patients overcome this fear:

1. **Create a Safe and Non-Judgmental Environment**:

- Establish trust by ensuring the patient that their concerns and experiences will be kept confidential and treated with sensitivity. Emphasize that the healthcare setting is a safe space for discussing personal issues.

2. **Normalize the Condition**:

- Educate the patient about incontinence as a common medical issue that affects many people, including those close to them. Share statistics and stories that illustrate the prevalence of incontinence to reduce stigma.

3. **Empathetic Listening**:

- Actively listen to the patient's fears and concerns. Show empathy and understanding for the emotions they are experiencing. Use statements like, "I understand this can be difficult to talk about."

4. **Provide Information and Education**:

- Offer comprehensive information about incontinence, its causes, and available treatments. Knowledge can empower the patient to have informed conversations with their loved ones.

5. **Discuss the Consequences of Silence**:

- Gently explain to the patient that keeping their condition a secret may have negative consequences for their emotional well-being and relationships. Highlight the potential benefits of opening up.

6. **Role-Play Conversations**:

- Help the patient prepare for conversations with family and friends by engaging in role-play exercises. This can boost their confidence and provide strategies for addressing potential questions or reactions.

7. **Encourage Gradual Disclosure**:

- Suggest that the patient start by confiding in a trusted individual, such as a close friend or family member, who is likely to offer understanding and support. Gradual disclosure may be less intimidating.

8. **Provide Resources**:

- Offer educational materials or recommend reputable sources where the patient can find additional information about incontinence. This can serve as a resource for them and their loved ones.

9. **Support Groups**:

- Recommend joining support groups or therapy groups for individuals dealing with incontinence-related issues. Connecting with others who share similar experiences can help patients feel less alone.

10. **Family Involvement**:

- If appropriate and with the patient's consent, involve family members in discussions about incontinence. Encourage an open and non-judgmental dialogue to promote understanding and support.

11. **Highlight Positive Outcomes**:

- Share stories or anecdotes of patients who have opened up about their incontinence and received positive responses from loved ones. Positive outcomes can provide hope and motivation.

12. **Reiterate Confidentiality**:

- Reinforce the importance of patient-doctor confidentiality to reassure the patient that their privacy will be protected.

13. **Set Realistic Expectations**:

- Help the patient set realistic expectations for how their loved ones might react. Explain that reactions can vary, but the goal is to promote understanding and support over time.

14. **Offer Continued Support**:

- Assure the patient that you are available to provide ongoing support and guidance throughout their journey, both in managing their incontinence and in addressing communication challenges with family and friends.

By using these strategies, physicians can help patients build the confidence and resilience needed to discuss their incontinence with loved ones and ultimately receive the support and understanding they deserve.

11. Q：How can physicians reassure and convince male patients when they refuse to mention their erectile dysfunction symptoms because of embarrassment and shame?

A: Reassuring and convincing male patients to discuss their erectile dysfunction (ED) symptoms when they are embarrassed and ashamed can be a delicate process. Here are some strategies for physicians to approach this situation sensitively:

1. **Create a Non-Judgmental Environment**:

- Establish trust and rapport by creating a safe, confidential, and non-judgmental space for the patient. Ensure they feel comfortable discussing personal issues.

2. **Normalize the Conversation**:

- Normalize discussions about sexual health by explaining that ED is a common medical condition that affects many men at some point in their lives. Let the patient know they are not alone in experiencing this.

3. **Use Empathetic and Non-Stigmatizing Language**:

- Use language that is neutral, empathetic, and non-stigmatizing when discussing ED. Avoid judgmental or derogatory terms.

4. **Initiate the Conversation**:

- Take the initiative to gently bring up the topic of sexual health and ask if the patient has any concerns or changes in their sexual function. Use open-ended questions to encourage them to share.

5. **Educate About ED**:

- Provide information about ED, its potential causes (both physical and psychological), and available treatments. Knowledge can alleviate some of the patient's concerns.

6. **Privacy and Confidentiality**:

- Reiterate the importance of patient-doctor confidentiality, emphasizing that their medical information will remain private.

7. **Empathize with Their Emotions**:

- Recognize the emotional impact of ED, including embarrassment and shame. Let the patient know that these feelings are understandable and common.

8. **Listen Actively**:

- Actively listen to the patient's concerns, fears, and feelings related to ED. Give them space to express themselves without interruption.

9. **Highlight Treatment Options**:

- Discuss the various treatment options available for ED, including lifestyle changes, medications, vacuum erection devices, injections, and implants. Tailor the discussion to the patient's preferences and needs.

10. **Share Success Stories**:

- Share stories of other patients who have sought treatment for ED and experienced improvements in their sexual function and overall well-being. Hearing positive outcomes can provide hope.

11. **Involve Partners**:

- If the patient is comfortable with it, encourage them to involve their partner in the conversation. Discussing ED as a couple can be beneficial and reduce the patient's feelings of isolation.

12. **Referral to Specialists**:

- If needed, consider referring the patient to a urologist or sexual health specialist who has expertise in treating ED. These specialists can provide additional support and guidance.

13. **Counseling and Therapy**:

- Suggest counseling or therapy for the patient, either individually or as a couple. Therapy can address the emotional aspects of ED and provide strategies for coping.

14. **Offer Follow-Up**:

- Schedule follow-up appointments to monitor the patient's progress, adjust treatment plans, and provide ongoing support. This shows the patient that you are committed to helping them improve their sexual health.

15. **Respect Their Pace**:

- Respect the patient's pace in discussing ED and seeking treatment. Let them know that they have control over their healthcare decisions and can take steps when they feel ready.

Reassuring and convincing male patients to discuss their ED symptoms requires patience, empathy, and a non-judgmental approach. By creating a supportive environment and providing information and treatment options, physicians can help patients overcome their embarrassment and take steps toward improving their sexual health.

12. Q：When male patients avoid having sex with their partners because of anxiety about sexual performance, how can physicians encourage them to confront this issue?

A： When male patients avoid having sex with their partners due to anxiety about sexual performance, physicians can play a role in encouraging them to confront and address this issue. Here are some steps to help patients in this situation:

1. **Create a Safe and Supportive Environment**:

- Establish a trusting and non-judgmental atmosphere in which the patient feels comfortable discussing their concerns. Emphasize that you are there to help and support them.

2. **Initiate a Conversation**:

- Gently initiate a conversation about the patient's sexual health and inquire about any changes or difficulties they have experienced. Use open-ended questions to encourage them to share their feelings and concerns.

3. **Normalize Performance Anxiety**:

- Explain that performance anxiety is a common issue that many people experience at some point. Normalize the fact that anxiety can affect sexual performance.

4. **Educate About Sexual Health**:

- Provide information about sexual health, the factors that can contribute to performance anxiety, and the importance of maintaining a healthy sexual relationship. Knowledge can help reduce anxiety.

5. **Discuss the Emotional Impact**:

- Explore the emotional impact of performance anxiety on the patient's well-being and their relationship with their partner. Encourage open and honest expression of feelings.

6. **Identify Triggers and Patterns**:

- Help the patient identify specific triggers or patterns that exacerbate their anxiety. Understanding these triggers can be a first step in addressing the issue.

7. **Offer Psychological Support**:

- Recommend therapy or counseling, either individually or as a couple, to address performance anxiety and related concerns. Cognitive-behavioral therapy (CBT) and sex therapy can be beneficial.

8. **Address Medical Causes**:

- Assess whether there are any underlying medical conditions contributing to the patient's sexual difficulties. If needed, recommend appropriate medical evaluations and treatments.

9. **Encourage Open Communication**:

- Stress the importance of open communication with their partner about their feelings and concerns. Encourage the patient to have an honest conversation with their partner about their anxiety.

10. **Couple's Counseling**:

- Suggest couple's counseling or therapy to facilitate open and constructive communication between the patient and their partner. This can help both parties understand each other's needs and concerns.

11. **Behavioral Techniques**:

- Teach relaxation techniques and mindfulness exercises to help the patient manage anxiety and stay focused on the present moment during sexual activity.

12. **Prescribe Medication**:

- In some cases, medication such as selective serotonin reuptake inhibitors (SSRIs) or phosphodiesterase type 5 (PDE5) inhibitors may be prescribed to address both performance anxiety and erectile dysfunction if present.

13. **Gradual Reintroduction to Intimacy**:

- Encourage the patient to gradually reintroduce intimacy with their partner at their own pace. Start with non-sexual forms of intimacy, such as cuddling or kissing, to rebuild trust and comfort.

14. **Set Realistic Expectations**:

- Help the patient set realistic expectations for sexual performance. Emphasize that intimacy is about connection and shared experiences, not just physical performance.

15. **Follow-Up and Support**:

- Schedule regular follow-up appointments to monitor the patient's progress and provide ongoing support. Adjust treatment plans as needed based on their experiences and feedback.

Encouraging patients to confront performance anxiety requires a patient-centered and empathetic approach. By addressing both the psychological and emotional aspects of this issue, physicians can help patients regain their confidence and improve their sexual well-being.

13. Q：When male patients are afraid of the possible side effects of sex therapy or medication, and thus refuse the relevant treatment, what should physicians do to persuade them?

A： When male patients are afraid of the possible side effects of sex therapy or medication and refuse the relevant treatment, physicians should employ a patient-centered and informative approach to address their concerns and potentially persuade them. Here are some steps to consider:

1. **Open and Non-Judgmental Communication**:

- Initiate an open and non-judgmental conversation to understand the patient's specific fears and reservations regarding sex therapy or medication. Create a safe space for them to express their concerns.

2. **Educate About Treatment Options**:

- Provide detailed information about the proposed sex therapy or medication, including how it works, potential benefits, and potential side effects. Emphasize that treatment decisions are collaborative.

3. **Discuss the Likelihood of Side Effects**:

- Discuss the likelihood and severity of potential side effects associated with the recommended treatment. Many side effects are rare or manageable.

4. **Individualized Risk-Benefit Analysis**:

- Engage the patient in an individualized risk-benefit analysis. Help them weigh the potential benefits of treatment against the likelihood and severity of side effects in their specific case.

5. **Alternative Treatment Options**:

- Explore alternative treatment options that may have fewer side effects or address the patient's concerns differently. Discuss the pros and cons of each option.

6. **Gradual Approach**:

- If the patient is hesitant about starting with medication, propose a gradual approach. For example, they could begin with sex therapy or non-pharmacological interventions and reconsider medication later if needed.

7. **Address Misconceptions**:

- Clarify any misconceptions or misinformation the patient may have about the proposed treatment. Provide evidence-based information to correct inaccuracies.

8. **Real-Life Success Stories**:

- Share success stories of other patients who have undergone similar treatment and have experienced positive outcomes. Hearing about others' experiences can provide reassurance.

9. **Involvement of Partner**:

- If applicable, involve the patient's partner in the discussion. Their support and understanding can be influential in the decision-making process.

10. **Consultation with Specialists**:

- If the patient remains hesitant, consider referring them to a specialist, such as a sex therapist or a urologist with expertise in sexual health. These specialists can provide more targeted information and address concerns.

11. **Shared Decision-Making**:

- Emphasize the importance of shared decision-making, where the patient plays an active role in choosing the treatment that aligns with their values and preferences.

12. **Regular Follow-Up and Monitoring**:

- Offer regular follow-up appointments to monitor the patient's progress and address any side effects or concerns that may arise during treatment. Knowing they will have ongoing support can provide reassurance.

13. **Respect Autonomy**:

- Ultimately, respect the patient's autonomy and their right to make decisions about their own healthcare. Avoid pressuring or coercing them into treatment.

14. **Time for Consideration**:

- If the patient remains uncertain, allow them time to consider their options. Encourage them to reach out with any questions or concerns that may arise after the initial discussion.

15. **Document Discussions**:

- Document the patient's concerns and the details of your discussions in their medical records. This helps maintain transparency and continuity of care.

Persuading patients to consider treatment for sexual issues when they are concerned about side effects requires patience, empathy, and a collaborative approach. By addressing their fears and providing thorough information, physicians can support patients in making informed decisions about their sexual health.

14. Q：When male patients are frustrated with their bodies because of erectile dysfunction or premature ejaculation and therefore refuse to undergo the necessary physical examination, how should physicians pacify and persuade them?

A：When male patients are frustrated with their bodies due to issues like erectile dysfunction (ED) or premature ejaculation and refuse to undergo the necessary physical examination, physicians should approach the situation with empathy and sensitivity. Here's how to pacify and persuade them:

1. **Establish Trust and Rapport**:

- Build trust and rapport with the patient by creating a supportive and non-judgmental environment. Assure them that their concerns will be treated with confidentiality and respect.

2. **Acknowledge Their Frustration**:

- Validate the patient's frustration and empathize with their feelings. Let them know that you understand their distress and that you are there to help.

3. **Normalize the Condition**:

- Explain that both ED and premature ejaculation are common medical issues that affect many men at some point in their lives. Normalize the discussion by emphasizing that seeking help is a proactive step.

4. **Educate About the Examination**:

- Clearly explain the purpose and procedure of the physical examination, assuring the patient that it will be conducted in a respectful and professional manner. Address any specific concerns they may have.

5. **Highlight the Importance of Diagnosis**:

- Emphasize the importance of a proper diagnosis to identify the underlying causes of their condition. Explain that a diagnosis is the first step toward effective treatment.

6. **Discuss Treatment Options**:

- Briefly discuss potential treatment options for ED or premature ejaculation, ranging from lifestyle changes and behavioral techniques to medication or therapy. Let the patient know that treatment is available.

7. **Gradual Exposure**:

- If the patient is hesitant about a full examination, suggest starting with less invasive components and gradually progressing to a complete examination as they become more comfortable.

8. **Involve a Specialist**:

- Consider referring the patient to a urologist or a specialist in sexual health if they continue to refuse the examination. Specialists often have more experience in addressing these concerns.

9. **Patient Autonomy**:

- Respect the patient's autonomy and their right to make decisions about their healthcare. Let them know that you are there to provide information and support their choices.

10. **Address Fear of Embarrassment**:

- Discuss the patient's fear of embarrassment during the examination. Explain that healthcare professionals are trained to handle sensitive issues with discretion and empathy.

11. **Offer Emotional Support**:

- Encourage the patient to involve a trusted friend or family member in their support network. Emotional support can be invaluable in overcoming fears.

12. **Regular Follow-Up**:

- Schedule follow-up appointments to monitor the patient's progress and address any concerns or questions they may have. Provide ongoing support throughout their healthcare journey.

13. **Counseling or Therapy**:

- Suggest counseling or therapy for the patient, either individually or as a couple, to address any emotional or psychological factors contributing to their condition and anxiety.

14. **Share Success Stories**:

- Share stories of other patients who initially had reservations about examinations but ultimately found them to be a positive and helpful experience.

15. **Document Discussions**:

- Document the patient's concerns, discussions, and the patient's decisions in their medical records. This helps maintain a record of their healthcare choices.

Patient education, empathy, and gradual exposure can help reassure and persuade patients to undergo necessary physical examinations when they are frustrated with their bodies due to sexual health issues. Always prioritize the patient's comfort and emotional well-being throughout the process.

15. Q：When male patients worry that their sexual dysfunction will affect their partner's satisfaction and thus develop low self-esteem or avoidance, how can physicians encourage and support them?

When male patients worry that their sexual dysfunction will affect their partner's satisfaction and develop low self-esteem or avoidance as a result, physicians can provide encouragement and support to help them address these concerns. Here are some strategies:

1. **Create a Safe and Supportive Environment**:

- Establish a safe and non-judgmental space for the patient to discuss their concerns. Make it clear that you are there to listen and offer support.

2. **Acknowledge Their Feelings**:

- Validate the patient's feelings of worry, low self-esteem, and avoidance. Let them know that these emotional responses are common but can be addressed.

3. **Normalize the Impact on Partners**:

- Explain that sexual dysfunction can affect both partners in a relationship and that it's important to work together to find solutions. Normalize the idea that open communication is key.

4. **Educate About Sexual Dysfunction**:

- Provide information about the specific sexual dysfunction the patient is experiencing (e.g., ED or premature ejaculation) and its potential causes. Knowledge can reduce anxiety.

5. **Emphasize Open Communication**:

- Stress the importance of open and honest communication with their partner. Encourage the patient to talk to their partner about their concerns and work together to find solutions.

6. **Couples Therapy**:

- Recommend couples therapy or counseling to facilitate constructive communication between the patient and their partner. A therapist can help address relationship issues and improve intimacy.

7. **Therapy for Self-Esteem**:

- If low self-esteem is a significant concern, suggest individual therapy or counseling to help the patient build self-confidence and address any negative self-perceptions.

8. **Behavioral Techniques**:

- Provide information about behavioral techniques and exercises that can be used to manage and improve sexual function. These may include mindfulness, sensate focus, and Kegel exercises.

9. **Treatment Options**:

- Discuss the various treatment options available for the patient's specific sexual dysfunction. Explain that treatment can be effective in improving sexual function and overall well-being.

10. **Shared Decision-Making**:

- Involve the patient in shared decision-making regarding their treatment. Collaborate on treatment plans that align with their values, preferences, and comfort level.

11. **Set Realistic Expectations**:

- Help the patient set realistic expectations for sexual function and satisfaction. Emphasize that sexual intimacy is about more than just physical performance.

12. **Couple's Sexual Health Education**:

- Educate both the patient and their partner about sexual health and function. Encourage both to learn together to foster understanding and empathy.

13. **Encourage Gradual Reintroduction to Intimacy**:

- If avoidance is an issue, suggest gradual reintroduction to intimacy and sexual activity, taking small steps at a pace that is comfortable for both partners.

14. **Follow-Up and Support**:

- Schedule regular follow-up appointments to monitor the patient's progress and provide ongoing support. Adjust treatment plans as needed based on their experiences and feedback.

15. **Refer to Specialists**:

- Consider referring the patient and/or their partner to a specialist in sexual health or relationship therapy for more targeted guidance and support.

By addressing emotional concerns, encouraging open communication, and offering guidance on treatment options, physicians can help male patients and their partners navigate the challenges of sexual dysfunction and work toward improved satisfaction and well-being.

16. Q：When male patients are reluctant to talk about their sexual function for fear of being ridiculed or criticized by their partners, family members or society, how should physicians reassure and encourage them?

When male patients are reluctant to talk about their sexual function due to fear of ridicule or criticism from their partners, family members, or society, physicians can take several steps to reassure and encourage them:

1. **Create a Safe and Non-Judgmental Space**:

- Establish trust and rapport with the patient by creating a safe and non-judgmental environment. Assure them that their concerns will be kept confidential.

2. **Normalize the Conversation**:

- Begin by normalizing discussions about sexual health. Explain that sexual dysfunction is a common issue and that many people experience it at some point in their lives.

3. **Acknowledge Their Fears**:

- Recognize the patient's fears of being ridiculed or criticized and assure them that their feelings are valid. Let them know that you are there to listen and support them.

4. **Educate About the Importance of Communication**:

- Explain the importance of open communication in addressing sexual health concerns and maintaining healthy relationships. Stress that honest conversations can lead to understanding and solutions.

5. **Emphasize Confidentiality**:

- Reiterate the importance of patient-doctor confidentiality. Assure the patient that their privacy will be protected, and their information will not be disclosed without their consent.

6. **Involve a Partner if Appropriate**:

- If the patient is comfortable, suggest involving their partner in the conversation. Offer to facilitate a joint discussion to address concerns and improve mutual understanding.

7. **Educate About Sexual Dysfunction**:

- Provide information about the specific sexual dysfunction the patient is experiencing (e.g., ED or premature ejaculation), its potential causes, and available treatments. Knowledge can empower them.

8. **Discuss Potential Impact on Relationships**:

- Talk about how unaddressed sexual dysfunction can affect relationships negatively. Highlight that addressing the issue can lead to improved intimacy and overall relationship satisfaction.

9. **Encourage Small Steps**:

- If the patient is still hesitant, suggest starting with small, gradual steps toward open communication. They can begin by sharing their concerns with a trusted friend or family member.

10. **Offer Resources**:

- Provide the patient with educational materials or recommend reputable sources where they can find more information about sexual health. Offer resources for both the patient and their partner.

11. **Referral to Specialists**:

- Consider referring the patient to a specialist in sexual health or relationship therapy who can provide more targeted support and guidance.

12. **Highlight the Benefits of Seeking Help**:

- Discuss the potential benefits of seeking help for sexual dysfunction, such as improved self-esteem, better relationships, and enhanced overall well-being.

13. **Regular Follow-Up and Support**:

- Schedule follow-up appointments to monitor the patient's progress and provide ongoing support. Let them know that you are committed to their care and well-being.

14. **Respect Autonomy**:

- Always respect the patient's autonomy and their right to make decisions about their healthcare. Encourage them to take the steps they are comfortable with.

Reassuring and encouraging male patients to talk about their sexual function is a crucial step in addressing their concerns and improving their overall well-being. By approaching the issue with empathy and understanding, physicians can help patients overcome their fears and seek the help they need.

17. Q：When male patients worry that their sexual dysfunction problems will lead to their partners leaving or being unfaithful, how should physicians advise and help them deal with this concern?

When male patients worry that their sexual dysfunction problems may lead to their partners leaving or being unfaithful, physicians can offer advice and support to help them address this concern. Here's how physicians can help:

1. **Listen Actively and Empathize**:

- Begin by actively listening to the patient's concerns and acknowledging their fears. Show empathy and understanding for the emotional distress they are experiencing.

2. **Normalize Relationship Concerns**:

- Explain that it's natural for individuals with sexual dysfunction to worry about the impact on their relationships. Normalize these concerns by emphasizing that they are not alone in feeling this way.

3. **Provide Education About Sexual Dysfunction**:

- Offer detailed information about the specific sexual dysfunction the patient is experiencing, its potential causes (both physical and psychological), and available treatments. Knowledge can help alleviate anxiety.

4. **Emphasize the Complexity of Relationships**:

- Discuss the complexity of relationships and the fact that they are built on a foundation of emotional connection, trust, and communication. Highlight that sexual intimacy is just one aspect of a relationship.

5. **Encourage Open Communication**:

- Stress the importance of open and honest communication with their partner. Encourage the patient to have a frank discussion with their partner about their concerns and fears.

6. **Couples Therapy or Counseling**:

- Recommend couples therapy or counseling to facilitate constructive communication between the patient and their partner. A trained therapist can help address relationship issues and improve understanding.

7. **Address Relationship Insecurities**:

- If the patient's fears are rooted in broader relationship insecurities, encourage them to explore these issues in therapy, either individually or as a couple.

8. **Reiterate the Importance of Emotional Connection**:

- Reinforce the significance of emotional connection, trust, and intimacy in relationships. Explain that partners often value emotional closeness more than physical performance.

9. **Discuss Coping Strategies**:

- Provide strategies for coping with sexual dysfunction together as a couple. This may include trying new forms of intimacy, exploring non-sexual ways to connect, or seeking treatment.

10. **Highlight Success Stories**:

- Share stories of other patients who have faced similar concerns and worked through them with their partners. Hearing about positive outcomes can provide hope.

11. **Discuss the Unpredictability of Relationships**:

- Explain that while sexual dysfunction can be challenging, relationships are influenced by many factors. Partners often stay committed and work together to overcome obstacles.

12. **Address Self-Esteem and Confidence**:

- If the patient's concerns are affecting their self-esteem and confidence, recommend therapy or counseling to help them build self-assurance and address negative self-perceptions.

13. **Offer Resources for Partners**:

- Provide educational resources and support for the patient's partner to help them understand and navigate the challenges of sexual dysfunction as well.

14. **Regular Follow-Up and Support**:

- Schedule regular follow-up appointments to monitor the patient's progress and provide ongoing support. Be available to address any new concerns or questions that may arise.

15. **Respect Patient Autonomy**:

- Always respect the patient's autonomy and their right to make decisions about their relationship and healthcare. Encourage them to take proactive steps that align with their values and needs.

Helping patients address concerns about their relationship due to sexual dysfunction involves a patient-centered and empathetic approach. By providing guidance and encouraging open communication, physicians can support patients in maintaining and improving their relationships despite these challenges.

18. Q：How can physicians convince male patients to seek help when they are experiencing intense anxiety and depression due to sexual dysfunction but fear that psychotherapy will be perceived as "weak"?

Convincing male patients to seek help for anxiety and depression related to sexual dysfunction when they fear that psychotherapy will be perceived as "weak" can be challenging but essential for their well-being. Physicians can employ several strategies to encourage them to seek help:

1. **Normalize Mental Health Concerns**:

- Begin by normalizing the idea that experiencing anxiety and depression related to sexual dysfunction is not a sign of weakness. Explain that mental health concerns can affect anyone and are a valid reason to seek help.

2. **Provide Information About Psychotherapy**:

- Educate the patient about what psychotherapy entails, emphasizing that it is a evidence-based treatment that can help individuals cope with emotional challenges, including anxiety and depression.

3. **Highlight the Mind-Body Connection**:

- Explain the strong connection between mental and physical health, emphasizing that addressing emotional well-being can positively impact physical health and sexual function.

4. **Share Statistics and Stories**:

- Share statistics and success stories about individuals, including men, who have benefited from psychotherapy for sexual dysfunction-related anxiety and depression. Real-life examples can reduce stigma.

5. **Emphasize Confidentiality**:

- Reiterate that psychotherapy is confidential, and what is discussed in therapy remains private. Assure the patient that their personal information will not be disclosed without their consent.

6. **Discuss the Impact on Quality of Life**:

- Talk about how untreated anxiety and depression can significantly impact one's overall quality of life, including relationships, work, and daily functioning.

7. **Encourage Self-Compassion**:

- Encourage the patient to practice self-compassion and remind them that seeking help is a courageous and self-caring act, not a sign of weakness.

8. **Involve a Support System**:

- Suggest involving a trusted friend or family member who can provide emotional support and accompany the patient to therapy sessions if they are comfortable with the idea.

9. **Emphasize the Importance of Treatment for Sexual Function**:

- Explain that addressing anxiety and depression through psychotherapy can also improve sexual function, as mental well-being is closely linked to sexual health.

10. **Offer a Choice of Therapists**:

- Provide options for therapists who specialize in sexual health or men's mental health. Let the patient choose a therapist who they feel comfortable with.

11. **Set Realistic Expectations**:

- Help the patient set realistic expectations for psychotherapy. Explain that it may take time and effort but can lead to significant improvements in mental health.

12. **Regular Follow-Up and Support**:

- Schedule regular follow-up appointments to check on the patient's progress and discuss their experiences with therapy. Show ongoing support and interest in their well-being.

13. **Address Any Misconceptions**:

- Address any misconceptions or myths the patient may have about psychotherapy, and provide evidence-based information to dispel these beliefs.

14. **Discuss Medication Options**:

- If necessary, discuss the potential benefits of medication in combination with psychotherapy to manage severe anxiety and depression. Explain that it can be a temporary solution.

15. **Respect Autonomy and Choice**:

- Ultimately, respect the patient's autonomy and their right to make decisions about their healthcare. Encourage them to take steps toward help at their own pace.

By using a combination of education, empathy, reassurance, and encouragement, physicians can help male patients understand the importance of seeking help for anxiety and depression related to sexual dysfunction and overcome any concerns about appearing "weak."

19. Q：When male patients begin to avoid all forms of intimacy, even simple physical touch, because of concerns about their sexuality, how should physicians encourage them to rebuild intimacy with their partners?

A： When male patients begin to avoid all forms of intimacy, including simple physical touch, due to concerns about their sexuality, physicians can play a crucial role in encouraging them to rebuild intimacy with their partners. Here are some strategies to help these patients:

1. **Create a Safe and Non-Judgmental Environment**:

- Establish trust and rapport with the patient by creating a safe and non-judgmental space for discussions. Assure them that you are there to support and understand their concerns.

2. **Acknowledge Their Concerns**:

- Validate the patient's concerns and feelings about their sexuality. Let them know that it's normal to have doubts and fears, and they are not alone in experiencing them.

3. **Normalize Intimacy**:

- Normalize the importance of intimacy in a healthy relationship and emphasize that it encompasses more than just sexual activity. Explain that intimacy includes emotional connection and physical closeness.

4. **Educate About Sexuality**:

- Provide education about sexuality, emphasizing that it is a complex and multifaceted aspect of human identity. Discuss the diversity of sexual orientations and expressions.

5. **Explore the Underlying Concerns**:

- Explore the specific concerns and fears that are driving the patient's avoidance of intimacy. Are they related to self-image, performance anxiety, or other factors? Understanding the root causes is essential.

6. **Counseling or Therapy**:

- Recommend counseling or therapy, either individually or as a couple, to address the patient's concerns about their sexuality. Therapists can help patients explore their feelings and develop strategies to rebuild intimacy.

7. **Encourage Gradual Steps**:

- Encourage the patient to take gradual steps toward rebuilding intimacy. Start with simple physical touch, such as holding hands or hugging, and progress at a pace that feels comfortable.

8. **Set Realistic Expectations**:

- Help the patient set realistic expectations for themselves and their partner regarding the process of rebuilding intimacy. Explain that it may take time and patience.

9. **Communication Exercises**:

- Suggest communication exercises that can help the patient and their partner express their feelings, desires, and concerns openly and without judgment.

10. **Emphasize Emotional Connection**:

- Stress the importance of emotional connection and trust in a relationship. Explain that fostering emotional intimacy can lay the foundation for physical intimacy.

11. **Involve a Partner if Appropriate**:

- If the patient is in a relationship, involve their partner in the conversation and encourage open communication. A supportive partner can play a vital role in rebuilding intimacy.

12. **Provide Resources**:

- Offer resources such as books, articles, or workshops on sexual health and intimacy that the patient and their partner can explore together.

13. **Regular Follow-Up and Support**:

- Schedule regular follow-up appointments to monitor the patient's progress and provide ongoing support. Be available to address any concerns or obstacles they may encounter.

14. **Address Self-Esteem and Self-Image**:

- If concerns about self-esteem and self-image are contributing to the patient's avoidance of intimacy, suggest therapy or counseling to help them build self-confidence.

15. **Respect Autonomy**:

- Respect the patient's autonomy and their right to make decisions about their relationships and healthcare. Encourage them to take positive steps at their own pace.

Encouraging patients to rebuild intimacy in the face of concerns about their sexuality requires a compassionate and patient-centered approach. By providing guidance and support, physicians can help patients work through their fears and move toward healthier, more fulfilling relationships.

20. Q：When male patients are afraid to discuss their sexual functioning with their doctors for fear of being misinterpreted or judged, what can physicians do to reassure and build their trust?

A：When male patients are afraid to discuss their sexual functioning with their doctors due to fear of being misinterpreted or judged, physicians can take specific steps to reassure them and build trust. Here are some strategies:

1. **Create a Non-Judgmental Environment**:

- Establish a safe and non-judgmental space where patients feel comfortable discussing their concerns. Use empathetic language and body language to convey understanding.

2. **Normalize the Conversation**:

- Normalize discussions about sexual health by explaining that sexual issues are common and affect many individuals. Encourage patients to view it as a normal aspect of healthcare.

3. **Encourage Open Dialogue**:

- Encourage patients to ask questions and express their concerns openly. Let them know that their thoughts and feelings are important and will be taken seriously.

4. **Use Inclusive Language**:

- Use inclusive and gender-neutral language when discussing sexual health to create a welcoming environment for patients of all genders and sexual orientations.

5. **Emphasize Patient-Centered Care**:

- Explain that healthcare is patient-centered, meaning that the patient's well-being and comfort are top priorities. Assure them that their concerns will be handled with care and confidentiality.

6. **Provide Information and Education**:

- Offer information about common sexual health issues, such as erectile dysfunction, premature ejaculation, or low libido. Knowledge can help patients understand their concerns better.

7. **Highlight the Importance of Sexual Health**:

- Stress the significance of sexual health as an integral part of overall well-being and quality of life. Explain that addressing sexual concerns can lead to improved overall health.

8. **Discuss Confidentiality**:

- Reiterate the importance of doctor-patient confidentiality. Assure patients that their information will not be shared without their consent, except when necessary for their care.

9. **Ask Open-Ended Questions**:

- Use open-ended questions to encourage patients to share their experiences and concerns in their own words. This can lead to more comprehensive discussions.

10. **Provide Reassurance**:

- Reassure patients that their feelings and experiences are valid and that they are not alone in facing sexual health challenges. Share that many effective treatments are available.

11. **Offer Supportive Resources**:

- Provide information about resources, support groups, or reputable websites where patients can find additional information and connect with others facing similar issues.

12. **Use Visual Aids**:

- Consider using visual aids, diagrams, or educational materials to facilitate discussions and enhance patient understanding.

13. **Involve a Trusted Partner**:

- If a patient is comfortable, suggest involving a trusted partner in discussions about sexual health. Partner support can be beneficial.

14. **Follow-Up and Continuity of Care**:

- Schedule regular follow-up appointments to monitor the patient's progress and provide ongoing support. Continuity of care can help build trust over time.

15. **Respect Autonomy and Choice**:

- Respect the patient's autonomy and their right to make decisions about their healthcare. Encourage them to take an active role in their sexual health.

Building trust and reassurance in discussions about sexual functioning is essential for patients to feel comfortable seeking the care they need. By taking these steps, physicians can foster an environment where patients are more likely to open up about their concerns and receive appropriate support and treatment.

21. How should physicians reassure and convince patients when they are reluctant to undergo the necessary rehabilitation for fear of post-surgical pain?

A：When patients are reluctant to undergo necessary rehabilitation after surgery due to fear of post-surgical pain, physicians can play a crucial role in reassuring and convincing them to proceed with rehabilitation. Here are some strategies to help address their concerns:

1. **Open and Honest Communication**:

- Initiate a candid and empathetic conversation with the patient. Encourage them to express their fears and concerns about post-surgical pain.

2. **Explain the Importance of Rehabilitation**:

- Clearly articulate the importance of rehabilitation in the patient's recovery process. Explain that it can enhance their overall outcomes, reduce complications, and improve their quality of life.

3. **Set Realistic Expectations**:

- Provide a realistic outlook on post-surgical pain. Explain that pain management is a key part of rehabilitation, and measures will be taken to minimize discomfort.

4. **Educate About Pain Management**:

- Offer information about pain management strategies, including medication, physical therapy techniques, and lifestyle adjustments. Highlight the effectiveness of these interventions in controlling pain.

5. **Emphasize Individualized Care**:

- Stress that rehabilitation programs are tailored to the patient's unique needs and pain tolerance. The healthcare team will work closely with the patient to ensure comfort and progress.

6. **Discuss Pain Control Measures**:

- Discuss specific pain control measures that will be in place during rehabilitation, such as medication options, non-pharmacological pain management techniques, and the use of assistive devices.

7. **Share Success Stories**:

- Share success stories of other patients who initially had concerns about post-surgical pain but benefited greatly from rehabilitation. Hearing about positive outcomes can provide reassurance.

8. **Involve the Rehabilitation Team**:

- Introduce the patient to the rehabilitation team, including physical therapists, occupational therapists, and pain management specialists. Let them know they will have a supportive and skilled team guiding their recovery.

9. **Offer Choices and Control**:

- Empower the patient by involving them in decision-making about their rehabilitation plan. Allow them to make choices and express preferences within the treatment options available.

10. **Address Alternative Treatment Options**:

- Discuss alternative or modified rehabilitation approaches that may be gentler or less painful but still effective. Tailor the plan to accommodate the patient's comfort level.

11. **Regular Follow-Up and Support**:

- Schedule regular follow-up appointments to monitor the patient's progress and adjust the rehabilitation plan as needed. Show ongoing support and availability for addressing concerns.

12. **Psychological Support**:

- If fear and anxiety about pain persist, consider involving a psychologist or counselor to help the patient manage their emotional responses and develop coping strategies.

13. **Peer Support**:

- Connect the patient with support groups or peer networks of individuals who have undergone similar surgeries and rehabilitation. Hearing from others who have faced similar challenges can be reassuring.

14. **Respect Autonomy and Consent**:

- Respect the patient's autonomy and their right to make decisions about their healthcare. Encourage them to voice their preferences and concerns throughout the process.

Reassuring and convincing patients to undergo necessary rehabilitation after surgery requires a patient-centered approach, clear communication, and a focus on addressing their specific fears and needs. By providing information, support, and empathy, physicians can help patients make informed decisions about their recovery.

22. What should physicians do to encourage patients when they are embarrassed by the physical changes caused by urinary diversion and refuse to change clothes or display their bodies in public?

A： When patients are embarrassed by the physical changes caused by urinary diversion surgery and refuse to change clothes or display their bodies in public, physicians can take several steps to encourage and support them:

1. **Empathetic Communication**:

- Begin by having an empathetic and non-judgmental conversation with the patient. Let them know that you understand their concerns and are there to help.

2. **Normalize Feelings**:

- Normalize the patient's feelings of embarrassment and body image concerns. Explain that it's common to have such emotions after surgery and that they are valid.

3. **Provide Information**:

- Offer detailed information about the urinary diversion procedure, its purpose, and its potential physical effects. Knowledge can help patients better understand and cope with their situation.

4. **Discuss Expected Changes**:

- Explain the expected physical changes that may occur after urinary diversion surgery, including changes in appearance and body function. Let the patient know that these changes are a part of their healing process.

5. **Reassure About Privacy**:

- Assure the patient that their privacy and dignity will always be respected. Explain the measures in place to maintain their confidentiality and protect their comfort during medical appointments.

6. **Share Success Stories**:

- Share success stories of other patients who have gone through similar experiences and eventually adapted to the changes. Hearing about positive outcomes can provide hope and encouragement.

7. **Involve a Support System**:

- Encourage the patient to involve a trusted friend or family member in their support network. Having someone they trust by their side can provide emotional support and encouragement.

8. **Suggest Professional Counseling**:

- Recommend professional counseling or therapy to address body image concerns and emotional distress. A therapist can provide coping strategies and emotional support.

9. **Provide Resources**:

- Offer resources, such as books, articles, or support groups, that focus on body image, self-acceptance, and adapting to life after surgery.

10. **Gradual Exposure**:

- Suggest a gradual exposure approach to help the patient become more comfortable with their changed body. Encourage them to start in a safe and private setting, such as at home, before venturing out in public.

11. **Collaborative Decision-Making**:

- Collaborate with the patient to make decisions about clothing choices and when and how they feel ready to display their bodies in public. Let them take an active role in regaining their confidence.

12. **Regular Follow-Up and Support**:

- Schedule regular follow-up appointments to monitor the patient's physical and emotional progress. Provide ongoing support and address any new concerns that may arise.

13. **Respect Autonomy**:

- Always respect the patient's autonomy and their right to make decisions about their body and healthcare. Encourage them to take steps at their own pace.

Encouraging patients who are embarrassed by physical changes after urinary diversion surgery requires sensitivity, understanding, and a patient-centered approach. By offering support, resources, and options for gradual adjustment, physicians can help patients regain their confidence and adapt to their new circumstances.

23. What can physicians do to encourage and support patients when they are concerned that their urinary flow pattern after diversion will cause them to avoid socializing by being looked at differently in public places?

A： Physicians can play a vital role in encouraging and supporting patients who are concerned that their urinary flow pattern after diversion surgery will cause them to avoid socializing due to fear of being looked at differently in public places. Here are some strategies to help address these concerns:

1. **Open and Non-Judgmental Communication**:

- Initiate an open and non-judgmental conversation with the patient to understand their concerns fully. Encourage them to express their fears and anxieties.

2. **Normalize Feelings**:

- Normalize the patient's feelings of concern and fear about urinary flow patterns. Let them know that these feelings are common, especially during the adjustment period.

3. **Provide Education**:

- Offer comprehensive education about the urinary diversion procedure and its effects on urinary flow. Explain the rationale behind the surgery and how it can improve their health.

4. **Discuss Expected Flow Patterns**:

- Explain the expected urinary flow patterns after the surgery, emphasizing that they are a part of the healing process. Address any misconceptions or concerns the patient may have.

5. **Reassure About Privacy**:

- Assure the patient that their privacy and dignity will be respected during medical appointments and in public spaces. Discuss strategies to maintain their comfort.

6. **Share Success Stories**:

- Share stories of other patients who have successfully adjusted to their urinary flow patterns and continued to lead active and fulfilling lives. Hearing about positive experiences can provide hope.

7. **Involve a Support System**:

- Encourage the patient to involve a trusted friend, family member, or caregiver in their support network. Having someone they trust by their side can provide emotional support and reassurance.

8. **Suggest Professional Counseling**:

- Recommend professional counseling or therapy to address anxiety, body image concerns, and social adjustment issues. A therapist can provide coping strategies and emotional support.

9. **Practice at Home**:

- Encourage the patient to practice managing their urinary flow patterns at home in a comfortable and private environment. This can help them gain confidence and control.

10. **Offer Adaptive Clothing and Supplies**:

- Provide information about adaptive clothing and supplies that can help manage urinary flow discreetly and comfortably in public. Discuss options that suit the patient's preferences.

11. **Gradual Exposure**:

- Suggest a gradual exposure approach to social situations. Encourage the patient to start with low-pressure situations and gradually work up to more challenging ones as they become more comfortable.

12. **Peer Support and Support Groups**:

- Connect the patient with support groups or peer networks of individuals who have undergone similar surgeries and adjustments. Peer support can be invaluable in sharing experiences and advice.

13. **Regular Follow-Up and Support**:

- Schedule regular follow-up appointments to monitor the patient's progress and provide ongoing support. Show empathy and a willingness to address evolving concerns.

14. **Respect Autonomy and Choice**:

- Always respect the patient's autonomy and their right to make decisions about their social interactions and healthcare. Encourage them to take steps at their own pace.

Encouraging and supporting patients concerned about their urinary flow patterns and social interactions requires a patient-centered approach, empathy, and sensitivity. By providing education, resources, and emotional support, physicians can help patients regain their confidence and successfully navigate public spaces.

24. When patients avoid intimacy with their partners because they are concerned about their sexual attractiveness due to physical changes after surgery, what should physicians do to help them build their self-confidence?

A： When patients avoid intimacy with their partners due to concerns about their sexual attractiveness following surgery, physicians can play a pivotal role in helping them build self-confidence and address these concerns. Here are some strategies to support patients in regaining their self-confidence:

1. **Open and Empathetic Communication**:

- Initiate a sensitive and empathetic conversation with the patient to understand their concerns fully. Encourage them to express their feelings and anxieties.

2. **Normalize Feelings**:

- Normalize the patient's feelings of insecurity about their physical appearance after surgery. Explain that such concerns are common, especially during the recovery and adjustment period.

3. **Provide Education**:

- Offer comprehensive education about the surgical procedure and its effects on physical appearance. Discuss the expected changes and reassure the patient that they are part of the healing process.

4. **Discuss Body Image Concerns**:

- Explore the specific body image concerns the patient is facing. Are there specific aspects of their appearance causing distress? Understanding their concerns is essential.

5. **Reassure About Partners' Support**:

- Emphasize that supportive and loving partners value emotional connection and intimacy beyond physical appearance. Share the importance of open communication with their partner.

6. **Recommend Professional Counseling**:

- Suggest professional counseling or therapy to address body image issues and self-confidence. A therapist can provide strategies to improve self-esteem and cope with insecurities.

7. **Self-Compassion and Self-Care**:

- Encourage the patient to practice self-compassion and self-care. Discuss self-affirmation techniques and activities that promote self-esteem and body positivity.

8. **Involve the Partner**:

- If the patient is comfortable, involve their partner in the conversation. Facilitate open and non-judgmental communication between the patient and their partner regarding intimacy and body image.

9. **Supportive Resources**:

- Provide information about resources, books, articles, or support groups focused on body positivity and rebuilding self-confidence.

10. **Suggest Physical Therapy or Exercise**:

- Recommend physical therapy or exercise programs that are appropriate for the patient's condition and can help improve their physical strength and body image.

11. **Gradual Reintroduction of Intimacy**:

- Encourage the patient to reintroduce intimacy with their partner gradually, starting with non-sexual forms of affection and intimacy. Let them progress at their own pace.

12. **Share Success Stories**:

- Share stories of other patients who have successfully regained their self-confidence and intimacy with their partners after surgery. Hearing about positive experiences can be motivating.

13. **Regular Follow-Up and Support**:

- Schedule regular follow-up appointments to monitor the patient's progress and provide ongoing support. Show empathy and be available to address evolving concerns.

14. **Respect Autonomy and Choice**:

- Always respect the patient's autonomy and their right to make decisions about their intimate relationships and healthcare. Encourage them to take steps that feel comfortable for them.

Helping patients rebuild self-confidence and navigate intimacy concerns after surgery requires a patient-centered approach, sensitivity, and understanding. By offering emotional support, resources, and a collaborative approach, physicians can assist patients in regaining their self-assurance and improving their intimate relationships.

25. How should physicians advise and encourage patients when they refuse to leave their homes because they fear that a new urinary flow pattern may lead to leaks or accidents?

A： When patients refuse to leave their homes due to fear that a new urinary flow pattern may lead to leaks or accidents, physicians can offer advice and encouragement to help them regain confidence and manage their concerns. Here are some strategies:

1. **Empathetic Communication**:

- Initiate a compassionate and understanding conversation with the patient to explore their specific fears and anxieties. Encourage them to express their concerns openly.

2. **Normalize Concerns**:

- Normalize the patient's concerns about urinary flow patterns and the fear of leaks or accidents. Explain that it's common for individuals to have these worries after changes in urinary function.

3. **Provide Education**:

- Offer comprehensive education about the new urinary flow pattern, how it functions, and the potential for leaks or accidents. Explain that adjustments take time.

4. **Reassure About Management Techniques**:

- Discuss various techniques and strategies for managing urinary flow patterns and preventing leaks. This may include recommended bathroom schedules, use of protective clothing or pads, and pelvic floor exercises.

5. **Discuss Fluid Intake and Diet**:

- Provide guidance on fluid intake and dietary choices that can help manage urinary function. Some patients may benefit from dietary modifications to reduce the risk of accidents.

6. **Recommend Pelvic Floor Exercises**:

- Encourage the patient to engage in pelvic floor exercises or physical therapy to strengthen pelvic muscles and improve bladder control.

7. **Gradual Exposure and Confidence Building**:

- Suggest a gradual exposure approach to leaving the home. Encourage the patient to start with short outings to familiar places and gradually increase the duration and distance over time.

8. **Offer Behavioral Therapy**:

- Consider referring the patient to a therapist who specializes in behavioral therapy for managing anxiety related to urinary issues. Therapists can teach coping strategies and relaxation techniques.

9. **Support Groups and Peer Networks**:

- Connect the patient with support groups or peer networks of individuals who have experienced similar concerns. Sharing experiences with others can be reassuring.

10. **Medication or Medical Devices**:

- If appropriate, discuss the potential use of medications or medical devices that can help manage urinary symptoms. Consult with a urologist or specialist as needed.

11. **Regular Follow-Up and Support**:

- Schedule regular follow-up appointments to monitor the patient's progress and provide ongoing support. Show empathy and be available to address evolving concerns.

12. **Respect Autonomy and Choice**:

- Always respect the patient's autonomy and their right to make decisions about their activities and healthcare. Encourage them to take steps at their own pace.

13. **Involve a Supportive Network**:

- Encourage the patient to involve a supportive network of friends or family who can provide emotional support and encouragement during outings.

14. **Set Realistic Goals**:

- Work with the patient to set realistic and achievable goals for gradually expanding their activities outside the home. Celebrate each milestone achieved.

15. **Provide Reassurance and Hope**:

- Reassure the patient that, with time and proper management techniques, their confidence can improve, and they can regain a sense of normalcy in their daily life.

Helping patients overcome the fear of leaving their homes due to urinary concerns requires a patient-centered and empathetic approach. By offering education, practical strategies, emotional support, and gradual exposure, physicians can assist patients in regaining their confidence and resuming their daily activities.

26. How should physicians reassure and persuade patients when they are anxious or stressed about learning the necessary self-care skills because they are concerned about the need for post-operative care after diversion?

A： When patients are anxious or stressed about learning the necessary self-care skills because they are concerned about the need for post-operative care after diversion surgery, physicians can take several steps to reassure and persuade them:

1. **Open and Supportive Communication**:

- Initiate an open and non-judgmental conversation with the patient. Let them express their fears and concerns about post-operative care.

2. **Normalize Anxiety**:

- Normalize the patient's anxiety and stress about post-operative care. Explain that it's common to have these feelings before a significant medical procedure.

3. **Provide Comprehensive Education**:

- Offer detailed information about the surgical procedure, the specific self-care tasks required, and the expected recovery process. Clear and honest communication can alleviate anxiety.

4. **Break Down the Process**:

- Break down the self-care tasks into manageable steps and provide a step-by-step plan for post-operative care. Ensure the patient understands each task and its importance.

5. **Demonstration and Practice**:

- If applicable, demonstrate self-care tasks, such as changing dressings or managing catheters, and allow the patient to practice under supervision. Hands-on experience can boost confidence.

6. **Reiterate Support**:

- Assure the patient that they won't be alone in their post-operative care journey. Emphasize the availability of healthcare professionals, caregivers, and support networks.

7. **Provide Written Materials**:

- Offer written instructions or educational materials that patients can refer to at home. Having written guidance can reduce anxiety about forgetting essential steps.

8. **Involve Caregivers or Family Members**:

- Encourage patients to involve a trusted caregiver or family member who can assist with post-operative care tasks. Knowing they have support can alleviate stress.

9. **Support Groups and Peer Networks**:

- Connect the patient with support groups or peer networks of individuals who have undergone similar surgeries and post-operative care. Sharing experiences can provide reassurance.

10. **Regular Follow-Up Appointments**:

- Schedule regular follow-up appointments to monitor the patient's progress and provide additional guidance and support. Show that you are committed to their well-being.

11. **Stress Coping Strategies**:

- Offer stress management techniques, such as deep breathing exercises, mindfulness, or meditation, to help the patient cope with anxiety and stress.

12. **Behavioral Therapy**:

- Recommend therapy or counseling for patients with severe anxiety or stress. A therapist can provide strategies for managing these emotions effectively.

13. **Highlight the Importance of Self-Care**:

- Explain that learning self-care skills is essential for a successful recovery and can lead to better long-term outcomes. Reinforce the benefits of self-care.

14. **Set Realistic Expectations**:

- Help the patient set realistic expectations for their recovery process and the time it takes to become proficient in self-care. Patience is crucial.

15. **Respect Autonomy and Choice**:

- Respect the patient's autonomy and their right to make decisions about their healthcare and self-care tasks. Encourage them to take an active role in their recovery.

Reassuring and persuading patients to overcome anxiety about learning self-care skills for post-operative care requires a patient-centered approach, empathy, and clear communication. By offering support, education, and practical guidance, physicians can help patients feel more confident and capable in managing their post-operative needs.

27. What should physicians do to encourage patients to return to normal life when they develop strong feelings of denial about their daily life after diversion and thus refuse to participate in family or work activities?

A： When patients develop strong feelings of denial about their daily life after urinary diversion surgery and refuse to participate in family or work activities, physicians can take several steps to encourage them to return to normal life. It's essential to approach this situation with sensitivity and empathy. Here are some strategies:

1. **Open and Supportive Communication**:

- Initiate a compassionate and non-judgmental conversation with the patient. Let them express their feelings of denial and concerns about returning to normal life.

2. **Normalize Feelings**:

- Normalize the patient's feelings of denial, acknowledging that it's common to experience such emotions after a significant life change like surgery.

3. **Provide Comprehensive Education**:

- Offer detailed information about the urinary diversion procedure, its benefits, and the expected recovery process. Ensure the patient understands the surgery's purpose and its impact on their daily life.

4. **Set Realistic Expectations**:

- Help the patient set realistic expectations for their recovery and return to normal life. Explain that it's a gradual process, and it's okay to take small steps.

5. **Highlight the Importance of Normalcy**:

- Emphasize the importance of resuming family and work activities for overall well-being and quality of life. Explain that participation can lead to improved physical and emotional health.

6. **Involve Family and Loved Ones**:

- Encourage the patient's family and loved ones to provide emotional support and understanding. Their involvement can help the patient overcome denial and re-engage in daily life.

7. **Set Achievable Goals**:

- Collaborate with the patient to set achievable goals for resuming activities. Start with small, manageable tasks and gradually increase their level of involvement.

8. **Therapeutic Counseling**:

- Suggest therapeutic counseling or support groups that specialize in helping patients cope with life changes and adjustments. Therapy can provide coping strategies and emotional support.

9. **Peer Support**:

- Connect the patient with peer networks or support groups of individuals who have undergone similar surgeries and faced similar challenges. Hearing from others can be reassuring.

10. **Stress Coping Strategies**:

- Teach stress management techniques, such as relaxation exercises, mindfulness, or meditation, to help the patient cope with denial and anxiety.

11. **Regular Follow-Up Appointments**:

- Schedule regular follow-up appointments to monitor the patient's progress and provide ongoing guidance and support. Show that you are committed to their recovery.

12. **Respect Autonomy and Choice**:

- Always respect the patient's autonomy and their right to make decisions about their daily activities. Encourage them to take steps at their own pace.

13. **Address Psychological Concerns**:

- If the patient's feelings of denial persist and significantly impact their daily life, consider involving a mental health professional for specialized care.

14. **Reinforce Positive Changes**:

- Celebrate and reinforce any positive changes and milestones achieved by the patient as they gradually return to normal life. Positive reinforcement can be motivating.

Helping patients overcome feelings of denial and re-engage in daily life after urinary diversion surgery requires patience, empathy, and a collaborative approach. By offering support, education, and a step-by-step plan, physicians can assist patients in regaining a sense of normalcy and well-being.

28. How should physicians reassure and advise patients to seek help when they are depressed because of physical changes after surgery, but fear that they will be seen as "weak" if they talk to others?

A： When patients are depressed because of physical changes after surgery but fear that seeking help will make them appear "weak," physicians can provide reassurance and guidance to encourage them to seek the support they need. Here are some strategies to approach this situation:

1. **Private and Respectful Conversation**:

- Initiate a private and respectful conversation with the patient. Let them know that you are there to listen and that their feelings are important.

2. **Normalize Seeking Help**:

- Normalize the act of seeking help for emotional challenges. Explain that it is common and courageous to seek assistance when facing difficult emotions, especially after a major life event like surgery.

3. **Educate About Mental Health**:

- Offer education about mental health, emphasizing that it is just as crucial as physical health. Explain that emotional well-being is an integral part of the healing process.

4. **Assure Confidentiality**:

- Reiterate the importance of doctor-patient confidentiality. Assure the patient that their information will not be shared without their consent, except when necessary for their care.

5. **Encourage Supportive Relationships**:

- Discuss the importance of sharing feelings with trusted friends or family members. Suggest that opening up to loved ones can provide valuable emotional support.

6. **Mention Professional Help**:

- Recommend professional help from a therapist, counselor, or mental health specialist who can provide guidance and coping strategies. Emphasize the expertise these professionals offer.

7. **Discuss Treatment Options**:

- Explain the various treatment options available for depression, including therapy and, if necessary, medication. Tailor recommendations to the patient's specific needs.

8. **Offer Support Groups**:

- Suggest joining a support group or therapy group specifically focused on individuals who have undergone similar surgeries or experienced similar physical changes. Sharing experiences can be comforting.

9. **Highlight Resilience**:

- Share stories of patients who have successfully managed their emotional challenges and achieved positive outcomes after seeking help. Highlight the strength it takes to seek support.

10. **Address Self-Stigma**:

- Discuss the concept of self-stigma, which can lead to feelings of weakness. Explain that seeking help is a sign of self-awareness and self-compassion, not weakness.

11. **Set Realistic Goals**:

- Collaborate with the patient to set realistic goals for their emotional well-being. Encourage them to take small steps toward seeking help, if that feels less daunting.

12. **Regular Follow-Up and Support**:

- Schedule regular follow-up appointments to check on the patient's emotional well-being and progress. Show ongoing support and empathy.

13. **Respect Autonomy and Choice**:

- Always respect the patient's autonomy and their right to make decisions about their mental health. Encourage them to take the steps they are comfortable with.

14. **Provide Helpline Information**:

- Offer information about mental health helplines and crisis hotlines where patients can speak confidentially with trained professionals.

By offering understanding, education, and a supportive approach, physicians can help patients overcome the fear of seeking help for depression after surgery. Encouraging them to address their emotional well-being can lead to improved mental health and a better overall recovery experience.

29. When patients are afraid to discuss their post-surgical quality of life or psychological problems with their physicians or health-care providers, how can physicians build their trust and encourage them to open up their communication?

A： Building trust and encouraging open communication between patients and healthcare providers is essential when patients are afraid to discuss their post-surgical quality of life or psychological problems. Here are some strategies to help physicians foster trust and create an environment where patients feel comfortable opening up:

1. **Establish a Patient-Centered Approach**:

- Demonstrate genuine care and empathy. Show patients that their well-being is your top priority, and their concerns are valued.

2. **Active Listening**:

- Practice active listening by giving patients your full attention when they speak. Allow them to express themselves without interruption or judgment.

3. **Create a Safe and Non-Judgmental Space**:

- Assure patients that the conversation is confidential and that you are there to provide support, not to judge. Create a safe and non-threatening environment.

4. **Normalize Open Communication**:

- Normalize the act of discussing post-surgical quality of life and psychological issues. Let patients know it's common to have concerns and that they are not alone.

5. **Build Rapport Over Time**:

- Foster an ongoing relationship with the patient. Over time, trust often grows as patients become more familiar with their healthcare providers.

6. **Ask Open-Ended Questions**:

- Use open-ended questions to encourage patients to share their thoughts and feelings. Questions like "Can you tell me more about how you've been feeling?" invite deeper conversations.

7. **Empower Patient Autonomy**:

- Respect and support the patient's autonomy and their right to make decisions about their healthcare. Encourage them to actively participate in their treatment plan.

8. **Use Plain Language**:

- Avoid medical jargon and use plain, understandable language. Patients may be more willing to discuss their concerns when they fully grasp the information.

9. **Demonstrate Empathy and Validation**:

- Show empathy and validate the patient's feelings. Use statements like "I understand this must be difficult for you" to convey your understanding.

10. **Offer Information and Education**:

- Provide information about the potential psychological challenges and quality-of-life issues that can arise after surgery. Knowledge can empower patients to discuss their concerns.

11. **Involve a Support System**:

- Encourage patients to involve a trusted family member or friend in their healthcare discussions. Having someone they trust present can provide emotional support.

12. **Refer to Specialists**:

- If needed, refer patients to specialists or therapists who can address their psychological and quality-of-life concerns. Collaborate with other healthcare professionals when appropriate.

13. **Follow Up Regularly**:

- Schedule regular follow-up appointments to check on the patient's well-being and address any concerns that may arise over time. Show ongoing support.

14. **Use Telehealth Options**:

- Offer telehealth or remote consultation options, which may make some patients feel more comfortable discussing sensitive topics from the privacy of their own space.

15. **Cultural Sensitivity**:

- Be sensitive to cultural differences and beliefs that may impact a patient's willingness to discuss certain topics. Respect cultural diversity and preferences.

16. **Provide Written Resources**:

- Offer written materials or resources that patients can review in their own time. These materials can help patients prepare for discussions about their concerns.

Building trust and encouraging open communication is an ongoing process that requires patience and sensitivity. By creating a supportive and empathetic healthcare environment, physicians can help patients feel more comfortable discussing their post-surgical quality of life and psychological issues.

30. How should physicians reassure and persuade patients when they refuse to undergo necessary medical examinations or treatments due to fear of re-operation or treatment that may be required after surgery?

A： When patients refuse to undergo necessary medical examinations or treatments due to fear of re-operation or additional treatments after surgery, physicians should employ a compassionate and reassuring approach to persuade them. Here are some strategies to address this situation:

1. **Open and Empathetic Communication**:

- Initiate a sensitive and understanding conversation with the patient. Allow them to express their fears and concerns without judgment.

2. **Normalize Concerns**:

- Normalize the patient's concerns about the need for re-operation or additional treatment. Explain that such considerations are part of the healthcare process and are taken seriously.

3. **Provide Comprehensive Information**:

- Offer detailed information about the medical examination or treatment in question. Explain the purpose, benefits, and potential outcomes. Ensure the patient fully understands the necessity.

4. **Clarify Risks and Benefits**:

- Discuss the potential risks and benefits of the examination or treatment. Highlight the benefits of early detection and intervention, which can lead to better outcomes.

5. **Highlight Success Stories**:

- Share success stories of patients who have undergone similar examinations or treatments and had positive results. Hearing about others' experiences can provide reassurance.

6. **Involve a Second Opinion**:

- Offer the option of seeking a second opinion from another trusted healthcare provider. Sometimes, a second perspective can alleviate concerns and provide clarity.

7. **Discuss Alternatives**:

- If applicable, explore alternative examinations or treatments that may be less invasive or have fewer associated risks. Collaborate with the patient to find the best approach.

8. **Address Fear of Re-operation**:

- If the patient fears re-operation, explain that it is a last resort and that many issues can be managed with less invasive measures. Reiterate that the healthcare team's goal is to avoid re-operation when possible.

9. **Emphasize Patient Autonomy**:

- Respect the patient's autonomy and their right to make decisions about their healthcare. Encourage them to actively participate in the decision-making process.

10. **Provide Emotional Support**:

- Offer emotional support and reassurance throughout the decision-making process. Let the patient know you are there to support them at every step.

11. **Consult a Specialist**:

- If the patient's concerns are particularly complex or related to a specific aspect of their surgery, consider involving a specialist or surgeon with expertise in that area.

12. **Address Misconceptions**:

- Clarify any misconceptions or misunderstandings the patient may have about the examination or treatment. Ensure they have accurate information.

13. **Offer Time for Reflection**:

- If the patient remains hesitant, offer them time to reflect on the decision. Sometimes, patients may need additional time to process their feelings and make informed choices.

14. **Regular Follow-Up**:

- Schedule regular follow-up appointments to monitor the patient's condition and discuss their decision. Show ongoing support and flexibility.

15. **Engage Family Support**:

- Encourage the involvement of trusted family members or friends who can provide emotional support and offer insights.

16. **Telehealth Options**:

- Offer telehealth or remote consultation options, which may make some patients feel more comfortable discussing their concerns from the privacy of their own space.

It's essential to respect the patient's autonomy and collaborate with them to address their fears and concerns. By providing information, emotional support, and options, physicians can help patients make informed decisions about necessary medical examinations and treatments.
